# Paving the Way to the Fuel of the Future—Nanostructured Complex Hydrides

**DOI:** 10.3390/ijms24010143

**Published:** 2022-12-21

**Authors:** Cezar Comanescu

**Affiliations:** 1 National Institute of Materials Physics, 405A Atomiștilor Str., 77125 Magurele, Romania; cezar.comanescu@infim.ro; 2 Faculty of Physics, University of Bucharest, 405, Atomiștilor Str., 77125 Magurele, Romania

**Keywords:** hydrogen, complex hydride, energy, nanoconfinement, destabilization, nanomaterials, MOF, additives, reversibility

## Abstract

Hydrides have emerged as strong candidates for energy storage applications and their study has attracted wide interest in both the academic and industry sectors. With clear advantages due to the solid-state storage of hydrogen, hydrides and in particular complex hydrides have the ability to tackle environmental pollution by offering the alternative of a clean energy source: hydrogen. However, several drawbacks have detracted this material from going mainstream, and some of these shortcomings have been addressed by nanostructuring/nanoconfinement strategies. With the enhancement of thermodynamic and/or kinetic behavior, nanosized complex hydrides (borohydrides and alanates) have recently conquered new estate in the hydrogen storage field. The current review aims to present the most recent results, many of which illustrate the feasibility of using complex hydrides for the generation of molecular hydrogen in conditions suitable for vehicular and stationary applications. Nanostructuring strategies, either in the pristine or nanoconfined state, coupled with a proper catalyst and the choice of host material can potentially yield a robust nanocomposite to reliably produce H_2_ in a reversible manner. The key element to tackle for current and future research efforts remains the reproducible means to store H_2_, which will build up towards a viable hydrogen economy goal. The most recent trends and future prospects will be presented herein.

## 1. Introduction

The EU’s ambition to be the first continent that completely replaces fossil fuels with alternative, renewable energy sources appears to face many difficulties. The related cost and scarcity of resources add to the carbon-neutral goal, making it even harder to identify a sustainable means to produce this energy. Renewable energy sources may be tracked to the action of the sun or wind, producing electricity that can be stored (batteries) or utilized to split H_2_O in oxygen and hydrogen. Out of the possible alternatives, hydrogen (H_2_) has emerged as the source capable to produce energy in an environmentally friendly way, without releasing CO_2_ or NO_x_ gases that are held responsible for the air pollution, smog and greenhouse effect worldwide. Modern trends led to a differentiation of H_2_ systems by their source, and nine color codes are actively used today in this purpose: white (naturally occurring hydrogen), red (high temperature, catalytic water splitting using electricity from nuclear plant), pink (electrolysis of water using energy from a nuclear plant), purple (combined chemo thermal electrolysis of water using nuclear power), turquoise (thermal splitting of methane via pyrolysis, C is removed in solid form), brown/black (hydrogen produced from coal: bituminous—black color, and lignite—grey color; both CO and CO_2_ gases are released in the atmosphere adding up to current pollution), gray (fossil fuels/steam methane reforming; CO_2_ is released in atmosphere), blue (fossil fuel; CO_2_ is captured and stored underground, hence blue stands for a carbon-neutral process to produce H_2_) and green (water electrolysis, using renewable electricity; no CO_2_ is released, water splitting produces only H_2_ and O_2_, being compliant with an anticipated zero-carbon policy). Hydrogen is the lightest element known (Z = 1) and the most abundant element in the universe, exhibiting unique properties like high diffusivity, low density, low boiling point (−252.8 °C) and high gravimetric energy density—roughly three times higher than that of gasoline. 

**Figure 1:**
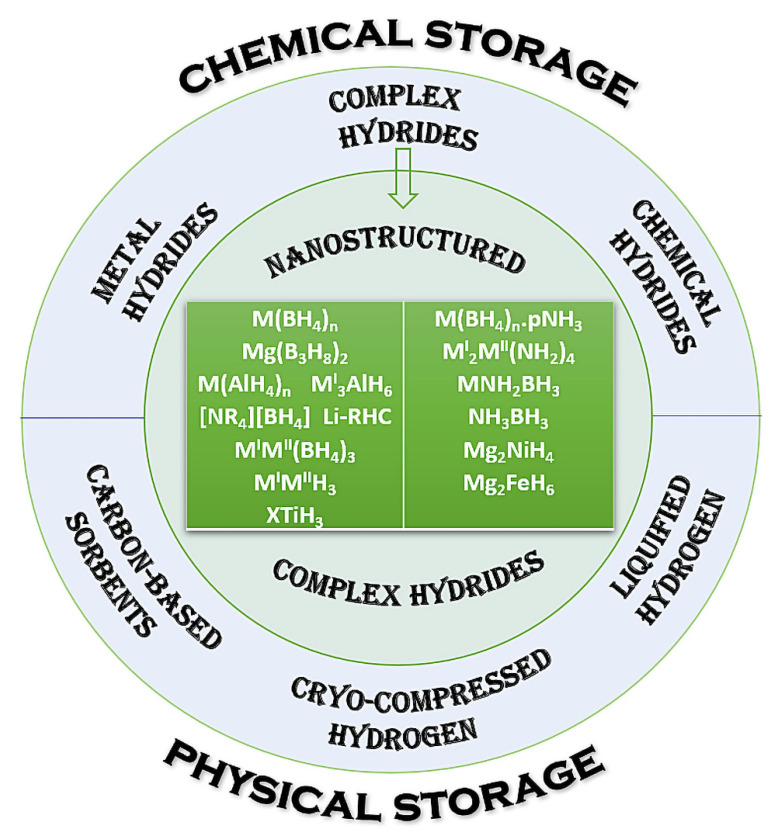
Nanostructuring technique and smart synergies are at the heart of physical and chemical properties improvements in complex hydride materials for solid-state hydrogen storage.

This review aims to cover the most significant milestones and recent advances in the field of nano-sized complex hydrides, which regained momentum due to the wide interest in shifting towards a carbon-neutral, green alternative fuel: hydrogen [1,2,3,4,5,6,7,8,9,10,11,12,13,14,15,16,17,18,19,20,21,22,23,24,25,26,27,28,29,30,31,32,33,34]. Complex hydrides have many added benefits compared to other means of storing hydrogen: solid-state storage, high gravimetric and volumetric density, potentially reversible behavior and competent structural and morphology-tuning via nanostructuring (Figure 1). 

**Figure 2:**
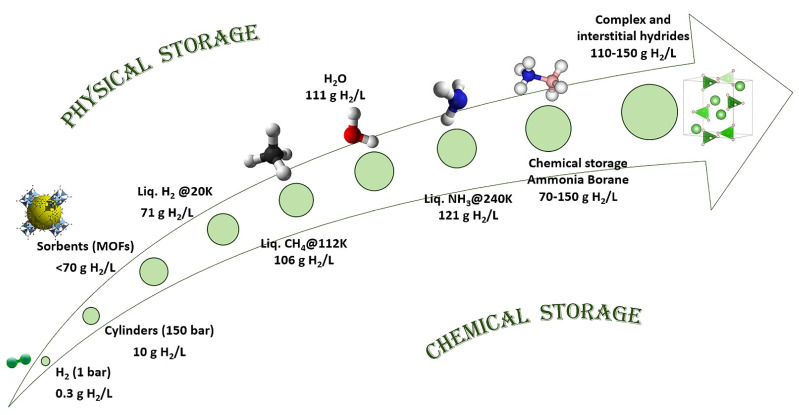
Compressed versus material-based hydrogen storage, arranged by increasing gravimetric content.

Various methods have been employed to store hydrogen throughout the years, which may be divided into physical (carbon-based sorbents, cryo-compressed and liquified hydrogen) and chemical storage (metal hydrides, complex hydrides and chemical hydrides) (Figure 1). Storing H_2_ in its molecular state presents a number of precautions and is generally regarded as posing safety risks (extreme temperature and high pressure) in addition to the challengingly-high overall cost, hence the solid-state storage options appear especially appealing by comparison. Hydrogen storage density is crucial for vehicular applications and has become a key requirement of US Department of Energy DOE [35]. The current goal of DOE is to obtain a fuel able to store 5.5 wt% H_2_ (1.8 kWh/kg, 1.3 kWh/L, costing $9/kWh) by 2025, and ultimately to achieve a hydrogen storage target of 6.5 wt% (2.2 kWh/kg, 1.7 kWh/L, at a cost of $8/kWh). A sustainable hydrogen energy can indeed be built using hydrogen as an energy carrier, but its storage in the solid state constitutes an obstacle (Figure 2) even today. A comparative overview shown in Figure 2 highlights the evolution of gravimetric hydrogen content from various sources H_2_ (1 bar, 0.3 g H_2_/L). These include lab cylinders (150 bar, 10 g H_2_/L), sorbents (MOFs, <70 g H_2_/L), liquid H_2_@20K (71 g H_2_/L), liquid methane CH_4_@112K (106 g H_2_/L), water H_2_O (111 g H_2_/L), liquid ammonia NH_3_@240K (121 g H_2_/L), ammonia borane NH_3_BH_3_ (70–150 g H_2_/L) and complex and interstitial hydrides (110–150 g H_2_/L). With gravimetric capacities of up to 150 g H_2_/L, complex hydrides surpass the capacity offered by water (111 g H_2_/L) and still represent the materials with the highest potential hydrogen storing capacity today. 

Moreover, even some of the most promising materials today, complex hydrides, have yet to overcome poor reversibility, sluggish kinetics and high desorption temperatures, in order to become mainstream energy carriers. With high storage capacities (Be(BH_4_)_2_: 20.7 wt% hydrogen, theoretical), metal borohydrides have emerged as very attractive candidates for solid-state hydrogen storage. Prime examples include LiBH_4_ (18.4 wt%) and Mg(BH_4_)_2_ (14.8 wt%) [4,9]. Still, several factors plague the wide acceptance of complex hydrides as H_2_ carriers: high dehydrogenation enthalpies [36,37], slow kinetics and/or side-reactions possibly leading to loss of boron (as borane B_2_H_6_ or higher boranes) and with it, the concurrent loss of reversibility. In addition, the theoretical hydrogen densities are rarely reached in practice, because complete dehydrogenation almost never occurs, and instead leads to formation of the corresponding metal hydride. Achieving sustained reversibility of complex hydride systems has been at the forefront of research efforts related to hydrogen storage materials and technologies [38,39,40]. Ongoing trends in hydrogen storage materials have expanded their scope for further use as electrolytes for batteries, harnessing the high ion conductivity exhibited by complex borohydrides—a behavior which can be tuned by various chemical strategies, or with temperature [41]. The molecular dynamics of anions and cations in polyborate salts comprise the reorientation of complex anions and translational diffusion of cations and complex anions and are key elements enabling the use of Li^+^ and Na^+^ *closo*-borates as solid electrolytes [42]. In this regard, nanotechnology has played a key role, enabling consistent improvements across the board regarding thermodynamic and kinetic behavior of complex hydrides, and the current review aims to overview the most important achievements in the field of nanostructured and nanoconfined complex hydrides. 

Recent advances in complex hydride chemistry have been supported by NMR studies [42,43], neutron scattering [44], EM electron microscopy (SEM, (HR)TEM) [45], in-situ XRD characterization [46,47] and thermodynamic data predictions brought about by assessing bond energies in B-H systems occurring during decomposition of borohydride species [48]. Further theoretical considerations in BET-isotherms allow for a better description of the porosity features of nano-sized scaffolds used for complex hydride materials [49]. However, not all characterization techniques are truly non-destructive, and certain adjustments of the parameters used are compulsory when dealing with sensitive materials such as hydrides [50]. A note on the importance of rigorous implementation of a hydrogen storage assessment strategy in energy storage compounds is reinforced by the divergent results reported by various groups on similar materials [51]. 

## 2. Classes of Complex Hydride Materials

The elemental abundance and ease of synthesis have polarized research in the area of complex hydrides around light metal borohydrides/tetrahydridoborates and alanates/tetrahydridoaluminates [52]. Moreover, alkali-metal complex hydrides (LiBH_4_, NaBH_4_, KBH_4_) serve as starting points for further borohydride synthesis via metathetic reactions, hence intensive studies have been carried out using these components. The improved kinetic behavior of Mg has motivated research on Mg(BH_4_)_2_ and Mg(AlH_4_)_2_ complex hydrides. As proof, no less than six polymorphs of Mg(BH_4_)_2_ have been reported to date, some with unexpectedly high morphological features (like high porosity, pore volume and surface area, γ-Mg(BH_4_)_2_) [4]. The synthesis of γ-Mg(BH_4_)_2_ by desolvation from the ethereal adduct indicates that the study of neutral molecule-stabilized adducts of borohydrides (with Et_2_O, THF, NH_3_, S(CH_3_)_2_, etc.) are well worth investigation, potentially yielding new, exciting polymorphs of otherwise known complex hydrides. In fact, ammoniates of common borohydrides have been investigated regarding their behavior in a nanoconfined state. Transition metal (TM) borohydrides and mixed (dual cation/anion) borohydrides have provided new tools to lower decomposition temperatures while featuring enhanced reversible behavior in hydrogenation studies. Lastly, many recent studies were devoted to ammonia borane (NH_3_BH_3_, AB) nanoconfinement in a variety of porous hosts, with promising results reported. 

### 2.1. Li-Based Systems

LiBH_4_ and LiAlH_4_ have long been pursued as promising hydrogen storage carriers, therefore the wide research output in the literature contains*—*at least as a starting point or as an intermediate*—*a Li-based system.

#### 2.1.1. LiBH_4_

LiBH_4_, the lightest borohydride known, has a very high theoretical storage capacity (18.4 wt%), and yet a high practical storage capacity (13.8 wt%) (Equation (1)) [53,54,55,56]. There are still thermodynamic and kinetic aspects that need to be overcome, and in this respect nanoconfinement became a proverbial tool providing overall noteworthy improvements of nanoconfined LiBH_4_ compared to neat lithium borohydride [57]. For instance, the onset desorption temperature (reported for bulk LiBH_4_ in the range 370–380 °C) is drastically reduced by ~300 °C through nanoconfinement in CNTs (2 nm; t_onset_ = 75 °C), or in Cu-MOF (0.9 nm; t_onset_ = 75 °C) [57]. Interestingly, ^11^B NMR shifts resulted from first principles calculations could shed more light into mechanistic decomposition pathways of nanoconfined LiBH_4_ [58]. Recent synthetic methods aim towards faster and nanosized-oriented syntheses of complex light borohydrides, either by using a surfactant approach [59], or by room temperature precipitation [60]. The effect of dopants (E = Na, K, Al, F, Cl) on hydrogen release energy profiles from E-doped LiBH_4_ was modelled using DFT and showed that H_2_ release is favored from two neighboring [BH_4_^−^] groups rather than a single borohydride anion, and that tensile strain can weaken interaction B-H, effectively facilitating H_2_ production [61]. On a similar note, ensemble machine learning protocol recently produced a predictive framework EoE with high accuracy (demonstrated on 3LiBH_4_ + AlF_3_ + 0.2TiF_3_ system, whose predictive behavior resembles well the actual curves for 1st and 2nd dehydrogenation), enabling performance prediction of LiBH_4_ with bi-component catalysts [62]. Many research efforts tackle nanoconfined LiBH_4_ from a Li-ion conductivity perspective, with direct application in solid electrolytes for batteries [63,64,65,66,67,68,69,70,71,72,73,74,75]. For instance, de Jongh et al. reported nanoconfinement of LiBH_4_ in ordered mesoporous silica (42 vol.% silica) with exceptionally high lithium mobility, reaching Li^+^ conductivity of 0.1 mS cm^−1^ at room temperature [76]. The thermal decomposition pathway of lithium borohydride entails a phase transition at 108–112 °C from *o*-LiBH_4_ to *h*-LiBH_4_ (with high Li^+^ ion conductivity), melting at 275 °C and a possibly stepwise decomposition (Li_2_B_12_H_12_ as the intermediate) in the range 400–600 °C to finally produce LiH, B and H_2_. [1,3,4]:

(1) $$o-{\mathrm{LiBH}}_{4\;\left(s\right)}\overset{108-112\;{}^{\circ}C}{\to }h-{\mathrm{LiBH}}_{4\;\left(s\right)}\overset{275\;{}^{\circ}C}{\to }{\mathrm{LiBH}}_{4\;\left(l\right)}\overset{400-600\;{}^{\circ}C}{\to }{\;\mathrm{LiH}}_{\left(s\right)}+B_{\left(s\right)}+\frac{3}{2}H_{2}↑$$

Rehydrogenation requires extreme conditions, an aspect which is in part responsible for the decreased hydrogen storage in subsequent release/uptake cycles (Equation (2)).

(2) $${\mathrm{LiH}}_{\left(s\right)}+B_{\left(s\right)}+\frac{3}{2}H_{2}\;\overset{\sim 700\;{}^{\circ}C,\;200{\;\mathrm{atm}\;H}_{2}}{\to }{\mathrm{LiBH}}_{4\;\left(s\right)}$$

Nanoconfinement has led to important achievements in improving reversible behavior of LiBH_4_, and a wide range of hosts have been investigated. Li-insertion was reported by Ngene et al. when destabilization of LiBH_4_ and LiAlH_4_ was achieved due to confinement in HSAG (high surface area graphite) leading to nanostructured complex hydrides able to fully dehydrogenate at 400 °C (Li forms instead of the LiH, which was surprising considering the high thermal stability of LiH which decomposes in bulk at ~900 °C) [77]. The reasoning for such improvement for the Li-intercalation and de-intercalation into turbostratic C nanoscaffolds, forming LiC_x_ intermediates which were identified by ^7^Li-MAS-NMR (MAS-magic angle spinning) [77]. 

Ngene et al. have used high surface area graphite (HSAG) after degassing and reducing (10%H_2_/Ar, 650 °C, 5 h) as a scaffold for the inclusion of LiBH_4_. Desorption was carried out at 400 °C under 1.1 bar H_2_ and showed different decomposition for fully confined LiBH_4_:

(3) $$12{\mathrm{LiBH}}_{4}\;+60C\;↔\;10{\mathrm{LiC}}_{6}+{\mathrm{Li}}_{2}B_{12}H_{12}+18H_{2}$$

In contrast to the nano-sized pathway (3), bulk LiBH_4_ follows the full decomposition pathway (4).

LiBH_4_ ↔ Li + B + 2H_2_ (4)

Rehydrogenation was possible under mild conditions (325 °C, 50 bar H_2_). Additionally, LiC_x_ was identified as a product instead of the usual LiH, due to Li-intercallation in graphitic carbon nanoscaffolds [77].

Using hollow carbon nanospheres, Lai et al. achieved a reduction in plateau pressure upon nanoconfinement of LiBH_4_ by solvent impregnation in a 30 wt% loading LiBH_4_@HCNWs composite (2–15 nm PSD). The dehydrogenation process had an early onset (t_onset,release_ = 50 °C), which peaked at ~100 °C, although the overall reversible hydrogen content was very modest at ~0.3 wt% [78].

A further modification of carbon-based scaffold was engineered by Gasnier et al., who used TM NP–decorated, N–doped graphene–rich aerogels of high textural properties: V_meso,in_ = 0.75 cm^3^/g before decoration and V_meso,f_ = 0.58 cm^3^/g after NP decoration (25 wt%, ~5% pore filling with TM = Ni/Co/NiCo) [79]. Metal confinement is more efficient in bigger mesopores, as confirmed by PSD data. A high gravimetric content of 12.2 wt% (des., 400 °C) was recorded for 30 wt%LiBH_4_-Co:GN nanocomposite. In the second cycle, the capacity decreased in the following order Ni > NiCo > Co regarding H_2_ wt% capacity, which again confirms Ni to be a superior catalyst (Reactions (5) and (6)). Potential physisorption by stacked graphene layers promoted by Ni [0]-desorption starts at ~100 °C [79].

LiBH_4_ + Co ↔ CoB + LiH + 1.5H_2_ (5)

LiBH_4_ + 2Ni ↔ Ni_2_B + LiH + 1.5H_2_ (6)

HNCs (hollow carbon nanospheres) used for LiBH_4_ nanoconfinement afforded a modest ~0.7 wt% partial reversibility, which was achieved at 300 °C and 6 MPa H_2_ [80]. The desorption temperature was as low as 100 °C, and solvent infiltration (partially reversible) was found to be superior to melt infiltration (no reversibility for melt–impregnated M(BH_4_)_n_-HCNs, at 350 °C, 6 MPa H_2_). M(BH_4_)_x_@HCNs nanocomposites had V_pore_ = 0.03 − 0.12 cm^3^/g and S_BET_ = 13.2 − 86.3 m^2^/g, and Ca(BH_4_)_2_-based composite showed the best textural parameters. Nanoconfinement seems to reroute dehydrogenation pathway from general Reaction (1) to (7).

(7) $${\mathrm{LiBH}}_{4\;\left(s\right)}\overset{\Delta }{\to }\frac{5}{6}{\;\mathrm{LiH}}_{\left(s\right)}+\frac{1}{12}{\;\mathrm{Li}}_{2}B_{12}H_{12\;\left(s\right)}+\frac{13}{12}{\;H}_{2}↑\;$$

LiBH_4_ was ground and then melt impregnated into mesoporous carbon hollow spheres (MCHSs) to afford LiBH_4_@MCHSs composite/pellets [81]. Wu et al. achieved physical confinement of lithium borohydride in double layers of the carbon nanobowls (DLCB). The overall decomposition conforms to Reaction (1). The recorded improvement was quantified by a strong decrease in activation energy, from 177.1 kJ/mol (neat LiBH_4_) to 121.4 kJ/mol (LiBH_4_@DLCB-2 nanocomposite), featuring a high volumetric hydrogen density of 82.4 g H_2_/L [81].

On the other hand, a similar reduction of E_a_ was achieved by Zhou et al. who melt-impregnated (MI) LiBH_4_ into activated charcoal (AC), reducing the activation energy for desorption to E_a,des_ = 121.1 kJ/mol for LiBH_4_/AC-MI samples that were able to sustain a reversible 6 wt% reversible hydrogen storage content [82].

Employing a high borohydride loading of 70 wt% in porous hollow carbon nanospheres (PHCNSs), Wang et al. achieved a 4.8 wt% reversible H_2_ storage, owed in part to the LiBH_4_–C surface interactions. The melt infiltration protocol for the most promising sample 60 wt%LiBH_4_-40 wt%PHCNSs consisted of rather typical parameters (300 °C, 30 min, 100 bar H_2_) [83].

When a carbon wrapped Fe_3_O_4_ host was loaded with 60 wt% LiBH_4_, very promising composite materials 6LiBH_4_@4p-Fe_3_O_4_@C were produced, with an early onset of 175 °C (7.8 wt% H_2_, 350 °C, 30 min.) [84]. The activation energy was further reduced compared to previously discussed examples, to E_a,des_ = 106.2 kJ/mol. Interestingly, although a part of the scaffold plays a sacrificial role (as it reacts with the borohydride source according to Reaction (8)), the formation of iron boride species (Fe_2_B, FeB) is regarded as highly beneficial for the reversibility of the system [84].

Fe_3_O_4_ + 4LiBH_4_ → Li_3_BO_3_ + Fe_2_B + FeB + B + 8H_2_↑ (8)

Sitthiwet et al. compacted LiBH_4_ and electrospun nanofibers of polyacrylonitrile (PAN) catalyzed by Ti(O^i^Pr)_4_, under high pressure (868 MPa), achieving ~5.2 wt% storage capacity for the resulting composite (ACNF-Ti) [85]. The high pressure applied during the compaction process produced rupture in ACNF-Ti, and subsequent agglomeration of hydrides during cycling (reduced performance in time); using a lower compaction pressure (434 MPa) led to superior kinetic and reversible behavior [85].

A synergy nanocatalyst-nanoconfinement was reported when LiBH_4_ (5–10 nm) was introduced in Graphene/Ni nanocrystals (2–4 nm) due to in-situ generation of both LiBH_4_ (from the reaction of LiH and C_6_H_15_NBH_3_ (9)), and Ni (from the reduction reaction of (C_5_H_5_)_2_Ni (10)) [86].

(9) $$\mathrm{LiH}+C_{6}H_{15}{\mathrm{NBH}}_{3}\;\to \;{\mathrm{LiBH}}_{4}\cdot C_{6}H_{15}N\;\overset{\Delta ,\mathrm{vacuum}}{\to }{\mathrm{LiBH}}_{4}+C_{6}H_{15}N$$

(C_5_H_5_)_2_Ni + H_2_ → Ni + 2C_5_H_6_ (10)

This strategy led to 9.2 wt% hydrogen storage, reversible under moderate conditions (300 °C), confirmed to be non-degrading even after the 100th cycle [86].

Exploring the role of scaffold porosity, Martínez et al. synthesized mesoporous carbon of various PSD (6, 10, 15, 25 nm) and used them to melt impregnate LiBH_4_ up to 90 vol% (10, 30, 50, 70, and 90 vol %; 300 °C, 60 bar H_2_, 30 min.) in a two-step impregnation procedure. It was observed that smaller pores fill faster, hence the need for a two-step procedure, and that destabilization of LiBH_4_ was achieved in the obtained composite, with LiH playing a destabilizing role. However, the reversibility of the system was rather poor [87].

Attempts to prepare nanosized LiBH_4_ (50–60 nm) by a single-pot solvothermal process were successful and afforded up to 12.1 wt% reversible behavior at 400 °C. Important thermodynamic parameters were also recorded: E_a.des_ = 147 kJ/mol (17% improvement over pristine) and E_a,rehyd_ = 81 kJ/mol (21% reduction relative to pristine) [88].

In a carefully–designed scaffold based on TiO_2_-catalyzed carbon, CNT@PC@TiO_2_ (CNT, carbon nanotubes; PC, porous carbon) and a high loading of LiBH_4_ (60 wt%) produced composite LiBH_4_:CNT@PC@TiO_2_, releasing 17.7 wt% hydrogen (500 °C). The CNTs facilitate the heat transfer, while the high release of H_2_ can be associated with a synergetic effect nanoconfinement—catalysis—surface interaction—thermal transfer [89].

Although moisture sensitive, LiBH_4_ was produced by a freeze-drying technique as a monohydride LiBH_4_·H_2_O that released 10 wt% from 50 °C to 70 °C. The very low onset and dehydrogenation peak were probably due to the strong affinity of H^+^ in the H_2_O ligand and H^−^ in the BH_4_ group [90]. However, the system LiBH_4_.H_2_O provides one-way desorption, as reversibility was not achieved (even under 350 bar H_2_) [90].

Various other attempts at catalyzing LiBH_4_ decomposition have been made. For instance, TiO-catalyzed (using Ti(OEt)_4_ precatalyst) lithium borohydride led to 9 wt% reversible hydrogen storage with enhanced kinetics and reversibility (74.4% H_2_ storage capacity retention, 10th cycle). The catalyst seems to also inhibit Li_2_B_12_H_12_ formation [91].

CoNiB-NPs loaded in carbon aerogels (CA) with pore size ~10 nm provided unexpected thermodynamic enhancements by a synergy nanoconfinement-catalysis, affording LiH desorption at 400 °C vs. 950 °C in bulk (14.5 wt% H_2_ released vs. 13.8 maximum for reaction step (1)). Metallic Li(0) was confirmed in desorption material (400 °C) by a 54.1 eV peak in XPS spectrum [92].

Suwarno et al. conducted a thermodynamic investigation of LiBH_4_ in nanoporous silica and carbon scaffolds by melt infiltration (300 °C, 5°/min, 25 min), after 10 min pre-mixing LiBH_4_ with scaffold for proper contact. Two types of LiBH_4_ mobile phases by quasi-elastic neutron scattering were observed, and the interfacial layer thickness was concluded to be an essential factor in hydrogen mobility. The interface interaction borohydride-scaffold was also quantified: 0.053 J/m^2^ (with SiO_2_) and 0.033 J/m^2^ (with C) [93].

Other scaffolds have been explored as well. Wang el al. employed Ce_2_S_3_ as additive for lithium borohydride, with LiBH_4_ + 20 wt% Ce_3_S_3_ showing favorable rehydrogenation (up to the fourth cycle; E_a_ was reduced from 181.80 kJ/mol for pristine LiBH_4_ to 151.82 kJ/mol for LiBH_4_ + 20 wt% Ce_3_S_3_) [94]. The reactivity of Ce_2_S_3_ with LiBH_4_, quantifiable via Reaction (11), afforded Li_2_S and CeB_6_ with co-catalytic dehydrogenation effects.

2LiBH_4_ + Ce_2_S_3_ → Li_2_S + 2CeS + 2B + 4H_2_ (11)

Activated carbon nanofibers (ACNF) with very high surface areas (S_BET_ = 2752 m^2^/g) were used by Plerdsranoy et al. to melt impregnate LiBH_4_ (pre-milled in SPEX Mill, 1 h, ball-to-powder BPR 30:1) [95]. The impregnation occurred in stages, hence prolonged time or multiple impregnations could lead to better results, an observation that could be extended to other melt impregnations as well.

The layered structure of 2D Ti_3_C_2_ utilized as hosts can bypass particle growth/agglomeration, providing excellent destabilization of LiBH_4_ [96]. Among the four compositions studied, the activation energy of LiBH_4_@xTi_3_C_2_, corresponding to mass ratios 2:1, 1:1, 1:2 and 1:3, was dependent on the heating profiles: E_a,1_ = 98.27 kJ/mol (12 °C/min) and E_a,2_ = 94.44 kJ/mol (8 °C/min).

Zero-valent metals like Ni(0) have showed many times more favorable activity in hydrogenation studies. A report from Chen et al. describes the synthesis of nanoporous nickel by dealloying Mn_70_Ni_30_ alloy in aq. (NH_4_)_2_SO_4_. LiBH_4_ was introduced by wet impregnation, and rehydrogenation was achieved at 450 °C and 8 MPa H_2_ [97].

When introduced via ball milling in a Fe_3_O_4_@rGO support (one-step hydrothermal route), LiBH_4_ produced nanocomposites LiBH_4_-Fe_3_O_4_@rGO with ~3.7 wt% hydrogen storage, and a reduced activation energy E_a_ = 79.78 kJ/mol [98].

Graphene in a mesoporous resorcinol–formaldehyde matrix was used to melt impregnate LiBH_4_ (30% pore volume filling), and enhanced [BH_4_^−^] mobility under nanoconfined state lowered peak temperatures compared to bulk counterpart. However, the poor reversibility recorded was due to LiH ejection from the pores during cycling [99].

CeF_3_-catalyzed activated carbon produced by the ball milling of components was utilized for loading LiBH_4_ (90 vol% of scaffold porosity) [100]. The wt% H_2_ released by the composite LiBH_4_-AC-CeF_3_ at 350 °C was 288 times higher than that of neat LiBH_4_, and a highly reversible 8.1 wt% was achieved in this catalyzed scaffold.

Wang et al. incorporated LiBH_4_ into modified carbon nanotubes: SWCNTs, andMWCNTs by ball milling, thus introducing local disorder and defects in CNTs. As a result, all incorporated LiBH_4_ was decomposed after 3 h at 500 °C [101].

Other fluorides were also explored as active catalysts, like NbF_5_ in mesoporous carbon MC via grinding (10 min), followed by LiBH_4_ infiltration MC-NbF_5_ (30 min, 300 °C, 140 bar H_2_, 85% vol% optimum filling) [102]. This approach led to a considerable reduction in apparent activation energy to E_a_ = 97.8 kJ/mol for LiBH_4_@MC-NbF_5_. Interestingly, nanoconfined samples do not exhibit a melting transition, in line with the disordered state of LiBH_4_. The synergistic role of nanoconfinement and nanocatalysis afforded a desorption that was 3.2 times faster in MC-NbF_5_ compared to neat MC (Reaction (12)).

2LiBH_4_ + NbH_x_ → 2LiH + NbB_2_ + (3 + x/2) H_2;_ ΔG = −113.97 kJ (12)

Dolotko et al. utilized SiS_2_ scaffolds to produce x LiBH_4_–SiS_2_ (x = 2–8) composites. Among them, six LiBH_4_—SiS_2_ was found to be the most promising material. The mixed borohydride decomposed according to (13), affording a reversible H_2_ capacity of 2.4 wt% [103].

Li_2_SiS_2_(BH_4_)_2_ → 2B + Li_2_S + 0.5Si + 0.5SiS_2_ + 4H_2_; ΔH = 32.5 kJ/mol H_2_ (13)

Nanoporous Mg scaffold was employed by Sofianos et al. for the melt-infiltration of LiBH_4_, according to the reaction Sequence (14)–(16).

NaH + MgH_2_ → NaMgH_3_ → Na(l) + Mg + 3/2H_2_ (14)

2LiBH_4_ + Mg → 2LiH + MgB_2_ + 3H_2_ (15)

LiH + Mg → LiMg + 1/2H_2_ (16)

Notably, Mg did not form MgH_2_ during LiBH_4_ melt-impregnation (300 °C, 60 bar H_2_) [104].

When LiBH_4_ was nanoconfined in AlN functionalized with O-atoms containing grafting groups, a frustrated Lewis pair (FLP)–like interaction occurs H^δ+^…H^δ−^, which in turn enhances the H_2_ release [105]. The AlN@LiBH_4 (_3:2 wt. ratio) yields the most complete desorption (2.8H/1LiBH_4_), while the formed borate Li_3_BO_3_ facilitates the decomposition and formation of [BH_4_]^−^ (Reaction (17)).

2LiBH_4_ + 6[HO-] → 2[BO_3_]^3−^+ 2LiH + 6H_2_ (17)

The carbon replica of 2D–ordered mesoporous silicfa MSU-H was loaded by up to 40 wt% LiBH_4_ by the incipient wetness method (0.1 M LiBH_4_ in tert–butyl–methyl-ether TBME, in 10–40 impregnation-evaporation steps) [106]. However, at lower loading of 8 wt%, LiBH_4_ remains completely confined into C-MSU-H mesopores (no XRD peaks belonging to borohydride phase). Palade et al. reported the partial rehydrogenation of LiBH_4_-C-MSU-H nanocomposite at 400 °C (100 bar H_2_, 2 h) [106]. The same group reported impregnation of LiBH_4_ into Mo:MSU-H catalyzed siloxanic materials close to melting (~270–280 °C) exhibiting partially reversible behavior [107].

Vellingiri et al. explored the role of LiB(OH)_4_, Li_2_CO_3_ and LiBO_2_ on LiBH_4_ @ SWCNTs and concluded that LiBO_2_ significantly enhances the dehydrogenation and rehydrogenation, in part due to H^+^ and H^−^ coupling between in situ formed Li^+^[B(OH)_4_]^−^, Li_2_^+^[CO_3_]^2−^ and Li^+^[BH_4_]^−^ [108].

Finally, aluminum derived from AlH_3_ was used as a scaffold for LiBH_4_ producing LiBH_4_/Al composite by BM (ball milling). Reversibility was found to depend on the growth of reaction products LiH, AlB_2_ and LiAl on the surface of Al* [109] (Table 1).

#### 2.1.2. LiAlH_4_

A closely-related complex hydride is LiAlH_4_ has for a while also polarized the research of hydrogen storage materials. It stands, along with LiBH_4_ and LiNH_2_, as some of the most promising complex hydrides featuring high hydrogen gravimetric content (Table 2). With a melting temperature of 150–175 °C (endothermic process), LiAlH_4_ can, in a three-step process, store up to 10.6 wt% and 96.7 g/L hydrogen when heated to 400–440 °C, which is still too high for stationary or mobile applications. In fact, the attainable hydrogen capacity is 7.9 wt% because only the two decomposition steps (Reactions (21) and (22)) of the total of three are thermodynamically accessible under acceptable conditions, and this represents another limitation of LiAlH_4_. Moreover, the individual steps in the decomposition process are predicted to be reversible at an unreasonably high H_2_ pressure (1000 MPa), which have rendered LiAlH_4_ a “one-off” energy storage material. Reviews concerning alanate-based systems and in particular LiAlH_4_ have emerged in the literature and discuss various thermodynamic and kinetic aspects of alanate-based systems [110,111,112,113]. The alkaline and alkaline-earth aluminum hydrides experience different crystal structure evolution when heated: alkali tetrahydridoaluminates follow a transformation from [AlH_4_]^−^ tetrahedra to isolated [AlH_6_]^3−^ octahedra, while alkali-earth counterparts have not been completely investigated. Yet, they decompose at slightly lower temperatures, showing various intermediate structures with chains of corner-shared octahedra, possibly due to their higher coordination number [114]. The catalyst scope utilized for LiAlH_4_ has been covered by recent reports [115]. Of particular interest remain destabilization strategies, aimed at lowering energetic barriers and thus lowering the temperature of individual de/re-hydrogenation steps. Nano-sizing alanate systems is an effective means to achieve this goal, and recent advances are summarized in Table 2. Leaching of active hydrogen storage material or of decomposition products outside of nano-porosity of the host can be of concern and could be a reason for degrading performance of complex hydrides with cycling.

Ngene et al. synthesized LiAlH_4_/HSAG composites by wet impregnation of LiAlH_4_ (0.8 g LiBH_4_ in 1 mL dried THF) in graphite, followed by drying under a dynamic vacuum (24 h, 35 °C). The nanocomposite LiAlH_4_/HSAG released H_2_ in three distinct steps, according to Equation (21): 150–175 °C; (22): 180–220 °C; and (23): 400–420 °C [77].

3LiAlH_4_ → Li_3_AlH_6_ + 2Al + 3H_2_ 5.3 wt% H_2_ (21)

Li_3_AlH_6_ → 3LiH + Al + 3/2 H_2_ 2.6 wt% H_2_ (22)

3LiH + 16C → 3LiC_6_ + 3/2H_2_ 3.1 wt% H_2_ (23)

LiH peaks disappeared from XRD diffractogram, hence full decomposition of LiH to LiC_6_ took place (at 300 °C). Non-porous supports (NPG, non-porous graphite) only favor reactions (21) and (22), to stop decomposition at the LiH stage, whereas porous graphite pushed the reaction forward to yield LiC_6_ as the final Li-containing product [77].

When nanoconfined in high surface area graphite (HSAG), LiAlH_4_ was studied by Wang et al. to provide reasoning for a different (de)hydrogenation pathway for LiAlH_4_ (nanoconfined) vs the known dehydrogenation pathway for LiAlH_4_ (bulk), following the set of reactions (21), (22) and (24) [116].

LiH + Al → LiAl + ½H_2_; 2.6 wt% H_2_; ΔH = 140 kJ/mol H_2_ (24)

Considering the loading of active alanate species in graphite support, the 0.6 wt% reversible storage accounts for ~30% reversibility of the LiAlH_4_ in the LiBH_4_@HSAG nanocomposite (3–15 nm). The rehydrogenated material could release H_2_ from 100 °C, providing evidence of reversibility via Li_3_AlH_6_ rehydrogenation—made possible by the nanostructured reaction participants [116].

Xia et al. used NiCo_2_O_4_@rGO supports to prepare (LiAlH_4_ + 7 wt% NiCo_2_O_4_@rGO) by ball milling [117]. LiAlH_4_ (ball-milled) released H_2_ with an early onset of 105.5 °C, while (LiAlH_4_ + 7 wt% rGO) started to release H_2_ at 108.3 °C. Adsorption energy of [AlH_4_^−^] at NiCo_2_O_4_ facilitated dehydrogenation reaction. The active catalyst was synthesized by a co-precipitation technique, from Ni^2+^ and Co^2+^ sources assisted by urea decomposition in water (NH_4_^+^, CO_2_, HO^−^, Equation (25)):

2NiCo_2_(OH)_2x_(CO_3_^2−^)_(2−x)_ · nH_2_O + O_2_ → 2NiCo_2_O_4_ + (2 − x)CO_2_ + (2x + n) H_2_O (25)

Switching to a doped carbon-based support, Cho et al. prepared N-doped CMK-3 carbon (NCMK-3) for LiAlH_4_ confinement by solvent infiltration [118]. The LiAlH_4_ was freshly recrystallized from Et_2_O before impregnation procedure, and the nitrogen N–sites (NCMK-3) were found to be critical for providing binding anchors (AIMD simulations) for metal cation Li^+^, a coordination mode responsible for hypothesized Li-N bond formation. Additionally, no reversibility was observed with undoped CMK-3. Li_3_AlH_6_ formation is suppressed by combined nanoconfinement and N-doping of the support, providing “thermodynamic stabilization” of metastable metal hydrides. Li_3_AlH_6_ phase is destabilized by nanosizing (does not form), and an overall Reaction (26) was proposed, supported by in-situ SAXS and WAXS data [118].

LiAlH_4_ → LiH + Al+ 3/2H_2_ (26)

Two dimensional layered materials of MXene type have also been investigated as supports for LiAlH_4_ confinement. LiBH_4_ and Ti_3_C_2_ were mixed by ball milling (Ar, 10 h) and five compositions were investigated in LiAlH_4_ + x wt% Ti_3_C_2_ system (x = 1, 3, 5, 10, and 15). Ti_3_C_2_ was found responsible for lowered Al-H bond energy in LiAlH_4_ and interfacial charge transfer/dehybridization of Al-H, which should facilitate the hydrogen release: E_a,1_ = 79.81 kJ/mol (31.3% improvement over pristine LiAlH_4_) and E_a,2_ = 99.68 kJ/mol (25.1% lower compared to bulk LiAlH_4_) [119].

Additives like SrFe_12_O_19_ were found to be beneficial for LiAlH_4_ dehydrogenation, providing important thermodynamic improvements associated with the first step (21) and second step (22): ΔE_a,1_ = 27 kJ/mol and ΔE_a,1_ = 15 kJ/mol [120]. The final state of alanate following dehydrogenation comprises LiH and Al phases, as confirmed by XRD data. The dehydrogenation pathway is otherwise unchanged by catalyst addition. The maximum amount of H_2_ released was 6.75 wt% and 6.5 wt% (10 wt% and 20 wt% SrFe_12_O_19_ doping) [120].

Li et al. introduced ZrC in LiBH_4_ by ball milling (Spex, BPR = 20:1, 1200 rpm, 60 min), without ZrC reacting with LiAlH_4_ during this process (XRD diffractogram) [121]. Even hand shaking mixing for 30 min caused catalytic effect to manifest when 5 mol% ZrC doping was used. Thermodynamic enhancements and increased surface defects triggered a lowering of E_a_ for elementary steps for 10 mol% ZrC-doped LiAlH_4_ sample, translating in lower onset dehydrogenation temperatures of 85.3 °C (first step, Equation (21), Δ = 90.7 °C relative to pristine) and 148.4 °C (second step, Equation (22), Δ = 57.8 °C relative to pristine). However, reversibility was not achieved (250 °C, 8 MPa H_2_) [121].

**Figure 3:**
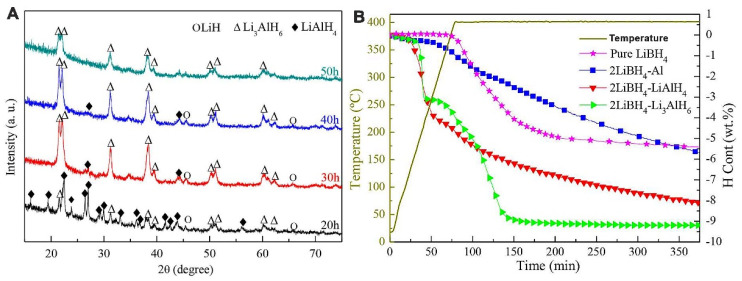
(**A**) XRD patterns of mixed powder of LiH and LiAlH_4_ after ball-milling for different numbers of hours; (**B**) TPD curves of pure LiBH_4_, 2LiBH_4_–Al, 2LiBH_4_–LiAlH_4_ and 2LiBH_4_–Li_3_AlH_6_ samples. Reprinted from ref. [122], Copyright © 2020 Li, Wu, Zhu, He, Xiao and Chen, distributed under the terms of the Creative Commons Attribution License (CC BY).

Other Al-based additives M (M = Al, LiAlH_4_, Li_3_AlH_6_) provided thermodynamic enhancements in 2LiAlH_4_-M composites [122]. Dehydrogenation temperature decreases in the order LiBH_4_ (469 °C) > 2LiBH_4_-Al (445 °C) > 2LiBH_4_-LiAlH_4_ (435 °C) > 2LiBH_4_-Li_3_AlH_6_ (416 °C). The most promising material among investigated samples was 2LiBH_4_-Li_3_AlH_6_ composite, which released 9.1 wt% H_2_ in 150 min. (Figure 3)

A heavier hexaferrite, that of barium BaFe_12_O_19_, was used by Sazelee et al. as a catalyst for LiAlH_4_ dehydrogenation [123]. Important thermodynamic improvements were registered over the pristine variant of the alanate: E_a1_ = 71 kJ/mol (ΔE_a,1_ = 32 kJ/mol) and E_a2_ = 90 kJ/mol (ΔE_a,2_ = 22 kJ/mol) associated with the first (Equation (21)) and second dehydrogenation step (Equation (22)). BaFe_12_O_19_ catalyst was introduced via ball milling, a process that reduced LiAlH_4_ particle size (from 10–50 µm-as received, to sub-µm), creating high surface defects and grain boundaries [123].

K_2_NbF_7_ was introduced as an additive in LiAlH4, using planetary ball milling [124]. Using a 10 wt% loading, the nanocomposite LiAlH_4_ and 10 wt% K_2_NbF_7_ showed improved thermodynamics with t_1_ = 90 °C, E_a,1_ = 80 kJ/mol, ΔE_a,1_ = 24 kJ/mol (21) and t_2_ = 149 °C; and E_a,2_ = 86 kJ/mol and ΔE_a,2_ = 26 kJ/mol (22). Desorption from catalyzed sample was 30 times faster than that of pure LiAlH_4_ [124].

Yang et al. have investigated three alanates: LiAlH_4_, NaAlH_4_ and Mg(AlH_4_)_2_ and drew a relationship between the catalytic effect and the cation’s electronegativity. A stronger catalytic effect (as seen for [Ni]) was recorded when the cation had a lower electronegativity [125]. For instance, the dehydrogenation temperature of NaAlH_4_ decreased from 140 to 111 °C. The reduced form of the catalyst, [Ni], was prepared from NiCl_2_ and ethylene glycol by sonication and thermal treatment, and was further incorporated in the alanates by ball milling for 90 min. using a BPR = 40:1. The obtained LiAlH_4_-5 wt% Ni-PCS composite suppressed the expansion of alanate during decomposition, in addition to the confirmed catalytic activity of [Ni] [125].

Other researchers have employed Ti–based catalysts to enhance dehydrogenation kinetics of alanates [126]. Zhao et al. prepared, by a one–step solvent method, the active catalyst TiO_2_/Hierarchically Porous Carbon (HPC), which showed evenly distributed TiO_2_ NPs (~10 nm) on honeycomb hollow hemispheres (600 nm diameter). By utilizing a solvent infiltration process, LiAlH_4_ was incorporated into TiO_2_/HPC support, at various proposed loadings (29, 37, 45 and 55 wt%). The authors observed a synergy TiO_2_—HPC, as their composite TiO_2_/HPC exhibits superior de-/rehydrogenation catalytic activity than either TiO_2_ or HPC. The composition 37 wt% LiAlH_4_—25 wt% TiO_2_/38 wt% HPC showed the lowest dehydrogenation temperature of 64 °C. The activation energies decreased in the order 63.5 ± 0.4 kJ/mol (HPC) > 57.8 ± 2.1 kJ/mol (TiO_2_) > 47.1± 3.5 kJ/mol (TiO_2_/HPC), confirming the TiO_2_/HPC as the most effective catalyst among the studied examples, and justifying the proposed synergy. The dehydrogenated composite comprises of Al and LiH, without complex alanates (LiAlH_4_ or Li_3_AlH_6_), while the broadened XPS spectra of Ti 2p were suggestive of multiple oxidation states of Ti, which promotes de/rehydrogenation performance of the nanoscaffold [126].

Employing a solvent impregnation method, Pratthana et al. infiltrated LiAlH_4_ 1.0 M (Et_2_O solution) in hollow carbon nanospheres (HCNs) to prepare nanoconfined LiAlH_4_@HCNs [127]. The choice of solvent (Et_2_O) was motivated by the better wettability of Et_2_O solution surface tension of 0.0165 J·m^−2^, compared to HCNs. As a result, the altered thermodynamic pathway was recorded, as described by the suggested Reaction (27) describing also a partial decomposition of LiH.

2LiAlH_4_ → 2LiH + 2Al + 3 H_2_ → 2LiAl + 1/2 H_2_ (27)

The altered mechanism proposed by Equation (27) was consistent with experimental findings showing that only LiH and Al were present in the sample at 150 °C, and thus (27) represents an alternate dehydrogenation pathway [127]. Additionally, the missing intermediate Li_3_AlH_6_ containing the complex anion [AlH_6_]^3−^ may be destabilized at the nanoscale and could split into two H- and [AlH_4_]^−^ due to the Jahn-Teller distortion effect [127].

On a related note, adducts of type LiAlH_4_.xMe_2_O also excluded formation of Li_3_AlH_6_ [128]. When regeneration of LiAlH4 was solvent-mediated, the reaction was tracked by in situ ^27^Al and ^7^Li NMR, confirming regeneration at 0 °C (Reaction (28)). The formation of adduct was proven crucial for reversibility. The solvate formation strategy proposed by Humphries et al. was constructed on previous reports where [Al(Ti)] alloying (room temperature, 13 bar H_2_) or LiAlH_4_.4THF adduct (120 °C, 350 bar H_2_) showed regeneration to be feasible [128].

(28) $$\mathrm{LiH}+\mathrm{Al}+3/2H_{2}\;\overset{{\mathrm{Me}}_{2}O}{↔}\;{\mathrm{LiAlH}}_{4}\cdot x{\mathrm{Me}}_{2}O$$

Using TiCl_3_ as the catalyst, Graetz et al. synthesized LiAlH_4_-2 mol% TiCl_3_ composites that achieved regeneration of LiAlH_4_ via LiAlH_4_ · 4THF, using solvent (THF) adducts from LiH and Ti-catalyzed Al desolvation (Reaction (29)) [129].

(29) $$\mathrm{LiH}+\mathrm{Al}+3/2H_{2}\;\overset{\mathrm{THF}}{↔}\;{\mathrm{LiAlH}}_{4}\cdot 4\mathrm{THF}$$

In an attempt to obtain alanate NPs, Pratthana et al. used various surfactants for size tuning in the range 10–200 nm [59]. Hexylamine (HXA, 99%), dodecylamine (DDA, *≥*99%), octadecylamine (ODA), *≥*99%), heptanethiol (HTT, 98%), dodecanethiol (DDT, *≥*98%), octadecanethiol (ODT, 98%), tetra-*n*-butylammonium bromide (TBAB, *≥*98%), tetra-*n*-octylammonium bromide (TOAB, 98%), tetra-*n-*decylammonium bromide (TDAB, *≥*99%) and tridecylic acid (TDA) were investigated. Surfactants have enabled high steric hindrance that can restrict alanate particle size. However, nanosizing and destabilization (even in 2–5 nm porosity), does not enable H_2_ release below 100 °C. Among grafted functionalities, the thiol (-SH) and amino (-NH_2_) functional groups lowered the H_2_ release temperature. LiAlH_4_ can reduce various groups (-COOH, -SH), reacting even with resulted –OH groups (Reactions (30)–(32)).

LiAlH_4_ + RCOOH → RCH_2_OH + LiOH + AlH_3_ (30)

LiAlH_4_ + 4RSH → LiAlH_4*−*n_(SR)_n_ + nH_2_ (31)

4LiAlH_4_ + 12ROH → LiAlH_4_ + 3LiAl(OR)_4_ + 12H_2_ (32)

A fluorographite scaffold (FGi contains 62 wt% F) was used to produce nanocomposites LiAlH_4_-xFGi (x = 0, 20, 30 and 40 wt%) by ball milling for 2 h with BPR 40:1 [130]. It was observed that LiAlF_4_ may potentially decrease the hydrogen storage capacity of LiAlH_4_@FGi, whereas using a 20 wt% FGi loading (t_onset_ = 103.2 °C) was not enough to stimulate a fast reaction. LiAlH_4_.30FGi released 3.23 wt% H_2_ at 62.7 °C in seconds. LiAlF_4_ also seems to prevail in LiAlH_4_.30FGi according to XRD data. Notably, the carbide species Al_4_C_3_ forms in sample LiAlH_4_-40FGi according to Equation (33) [130].

4LiAlH_4_ + 4CF → 4LiF + Al_4_C_3_ + C + 8H_2_ (33)

Reaction (33) also reduces E_a_ from 11.39 kJ/mol to −178.54 kJ/mol, enabling full dehydrogenation at 65 °C [130].

Finally, a hexagonal boron nitride (h-BN) host was used by Nakagawa et al. to produce LiAlH_4_/h-BN nanocomposites [131] by ball milling. The role of h-BN was compared to the desorption properties of LiAlH_4_/X (X = graphite, LiCl and LiI) composites, and a maximum storage capacity of 7.6 wt% was reported for LiAlH_4_/4 wt% h-BN. The Li-ion conductivity enhancement was also suggested for the obtained composites [131].

#### 2.1.3. Li_3_AlH_6_

Li_3_AlH_6_ was proposed and confirmed as a key intermediate in thermal decomposition of LiAlH_4_ (reactions (21) and (22)). Reports have described the overall improvement in the dehydrogenation of alanates when Li_3_AlH_6_ was destabilized through catalysis or nanoconfinement, in such a way that the first and second dehydrogenation steps collapsed into a single hydrogen release event [77,116,118,122,123,126,128,131]. In some cases, the intermediacy of Li_3_AlH_6_ was excluded based on experimental diffraction data [118,128]. In fact, when adducts of LiAlH_4_ with various ethers (Me_2_O, Et_2_O and THF, etc.) were employed to guide a regeneration route of spent alanates, typically, Li_3_AlH_6_ was excluded from the plausible intermediates [128]. The same is the case when N-doped ordered mesoporous carbon was used as the host, and the formation of Li_3_AlH_6_ was completely suppressed [118].

However, the synergistic effects of reactive hydride composites considerably improve the behavior of Li_3_AlH_6_. 2LiBH_4_-Li_3_AlH_6_ composite released 9.1 wt% H_2_ in 150 min [122]. Li et al. showed that among investigated composited 2LiAlB_4_ + M (M = Al, LiAlH_4_ and Li_3_AlH_6_), the sample 2LiBH_4_-Li_3_AlH_6_ exhibited the best results in hydrogenation studies [122]. The synthesis of Li_3_AlH_6_ was performed by milling LiH and LiAlH_4_ (Reaction (34)) for many hours, with a complete reaction being achieved by the 50th h mark, although clear formation of Li_3_AlH_6_ was confirmed by XRD even after 20 h of milling (Figure 3) [122].

(34) $$2\mathrm{LiH}+{\mathrm{LiAlH}}_{4}\;\to {\;\mathrm{Li}}_{3}{\mathrm{AlH}}_{6}$$

By the end of the hydrogen release programme of 2LiBH_4_ + Li_3_AlH_6_, the majority of the phases were identified as AlB_2_ and LiH, along with an apparently extraneous peak at about 2θ = 50°, which was not observed from 2LiBH_4_ + Al composite dehydrogenation products. The reversibility of the nanocomposite 2LiBH_4_ + Li_3_AlH_6_ still requires further research data [122]. 

On a similar note, but with a slight variation of the borohydride component, the NaBH_4_ + Li_3_AlH_6_ nanocomposite was studied by Yahya et al. [132] who pointed to thermodynamic destabilization, with best results when a 1:1 molar ratio of complex hydrides was used. Li_3_AlH_6_ was synthesized by the authors within 12 h using the same Reaction (34). The composite was able to dehydrogenate in two stages (170 °C, Li_3_AlH_6_ and 400 °C, NaBH_4_), and could reabsorb 6.1 wt% at 420 °C and 30 atm H_2_ in 60 min [132]. Improved dehydrogenation behavior was due to the formation of Na, Al and AlB_2_ which can act as catalysts for dehydrogenation steps. Indeed, the role of Li_3_AlH_6_ seems to be the destabilization of alkali metal borohydride [132]. It could be seen as a reservoir for active [Al] which results from the decomposition at 170 °C (Reaction (22)).

Indeed, this may react with NaBH_4_ according to Reaction (35) to produce the final dehydrogenation phases consisting of Na, AlB_2_ and remaining LiH (from Li_3_AlH_6_ decomposition).

2NaBH_4_ + Al → 2Na + AlB_2_ + 4H_2_ (35)

In contrast to previous rehydrogenation attempts in LiAlH_4_ system where 100 atm H_2_ and temperatures up to 260 °C were applied, the report from Yahya et al. shows temperature (420 °C) and destabilization (with Li_3_AlH_6_) to be key factors in achieving reversibility [132].

#### 2.1.4. LiNH_2_

Lithium amide (LiNH_2_) was used in the recent past as a component of RHC (reactive hydride composite) mixtures, many of which showed remarkable hydrogen storage properties, especially when coupled with main group borohydrides such as Mg(BH_4_)_2_. However, it was found that LiNH_2_ can also enhance Li-ion conductivity in LiBH_4_–LiNH_2_/metal oxide nanocomposites [133,134,135]. The main reason for observed enhancement was the partial anion substitution of [BH_4_]^−^ with [NH_2_]^−^, coupled with nanoconfinement in mesoporous oxide scaffolds, which allowed for a bump in ion conductivity from 10^−8^ S cm^−1^ for neat LiBH_4_, to 5 × 10^−4^ S cm^−1^ in LiBH_4_–LiNH_2_/metal oxide composites [135]. It was also inferred from the same study that the porosity of the host was essential for tuning conductivity features of the composite, even more so than the nature of the scaffold or the chemical interactions scaffold-amide/borohydride [133,134,135,136].

Hydrogen storage studies involving LiNH_2_ are rather scarce, but some reports refer to an interaction of LiNH_2_ with other complex hydrides. and the observed synergies in hydrogenation investigations [137]. Surprisingly, ball milling LiNH_2_ and Mg_2_FeH_6_ produced by a metathesis reaction Li_4_FeH_6_, (typically obtained from LiH and Fe at 700 °C and 5.5 GPa H_2_) (Reaction (36)).

Mg_2_FeH_6_ + 4LiNH_2_ → Li_4_FeH_6_ + 2Mg(NH_2_)_2_, ΔH = −92.8 kJ/mol (36)

**Figure 4:**
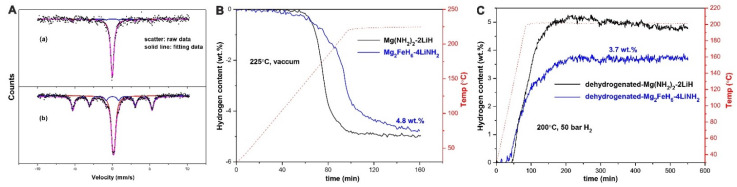
(**A**) ^57^Fe Mössbauer spectra: (**a**) as-prepared Mg_2_FeH_6_ and (**b**) Mg_2_FeH_6_–4LiNH_2_ -BM 12 h-200 °C; (**B**) Dehydrogenation curves of two post-milled samples (black: Mg(NH_2_)_2_–2LiH; blue: Mg_2_FeH_6_–4LiNH_2_); (**C**) Rehydrogenation curves of two samples (black: dehydrogenated–Mg(NH_2_)_2_–2LiH; blue: dehydrogenated–Mg_2_FeH_6_–4LiNH_2_). This figure was reprinted with permission from ref [137].

The resulting hydride nanocomposite Mg_2_FeH_6_-4LiNH_2_ was subjected to further de/rehydrogenation studies when ~4.8 wt% H_2_ (t_onset_ = 130 °C) was desorbed and 3.7 wt% H_2_ was re-absorbed, with slightly worse kinetics than the known and studied Mg(NH_2_)_2_-2LiH composite [137] (Figure 4). The regenerated form after rehydrogenation of the spent composite comprises of Mg(NH_2_)_2_, LiH and Fe.

#### 2.1.5. Li-RHC (Reactive Hydride Composite)

Practical implementation of RHC and of hydrides in general is hampered by the high operating temperature and rather low reversibility exhibited by such hydrogen-storing systems [138]. The behavior of RHC (a combination of hydride materials) can be enhanced by choosing proper selection of metal hydrides and complex hydrides, by significant thermodynamic alteration by metathesis reaction—which in turn will speed up kinetics and the reversibility of the RHC system. Collateral improvements were recorded in Li-ion conductivity studies conducted on nanoconfined RHC composites such as the LiBH_4_-LiNH_2_ system catalyzed by metal oxide NPs [135,139]. For instance, an enhancement of OMS (ordered mesoporous silica, of 1D type MCM-41 or 2D type SBA-15) afforded Li-ion conductivity 10 times higher for LiBH_4_-LiNH_2_/MCM-41 (1.16 × 10^−4^) compared to the nonconfined composite LiBH_4_-LiNH_2_ [139].

An overview of components of RHCs (Table 3) also allowed in-depth analysis of some of the chemical reactions involved in RHC—presented in Table 4.

While many research results are not always accompanied by a chemical reaction to account for the hydrogen released, there are some examples that justify the use of binary or tertiary systems (Reactions (37)–(45)) (Table 4). In some cases, like for the system 2NaAlH_4_ + Ca(BH_4_)_2_, the intermediacy of intermetallic components or derived complex hydrides (CaAlH_5_, CaH_2_, Al_4_Ca, Al_2_Ca) was inferred [138].

Many of these reactions are chain-reactions (one product, such as Al produced in Reaction (43) reacts with one of the reagents—MgH_2_—to produce the intermetallic Mg_17_Al_12_) releasing considerable amounts of hydrogen (Reaction (46)).

17MgH_2_ + 12Al → Mg_17_Al_12_ + 17H_2_ (46)

By corroborating reported reaction data with hydrogen storing properties (Table 3 and Table 4), it can be observed that Li-based RHC (Li-RHC) are the most widespread RHC investigated to date [138,140,141,142,143,144]. TEM investigations in LiBH_4_-MgH_2_ RHC catalyzed by 3TiCl_3_·AlCl_3_ additives revealed MgB_2_ platelets (Reactions (40) and (41)) originating from potential nucleation centers including Mg or TiB_2_ and AlB_2_. The formation and identification of MgB_2_ in the reactive mixture pleads for the kinetic improvement brought about by MgB_2_ in the RHC [141]. It was also suggested that using additives providing a small atomistic misfit (1.7% relative to MgB_2_) could further enhance the behavior in hydrogenation studies [141].

Perhaps one of the first reactions to meet nanoconfined space, the RHC system LiBH_4_-MgH_2_, was reported by Nielsen et al. in 2010, when using a carbon aerogel of pore size ~21 nm (~722 m^2^/g, 1.1 cm^3^/g) [142]. The mixture 2LiBH_4_:1MgH_2_ follows the decomposition path described by reactions (40) and (41), that can be summed up by the overall Reaction (47).

2LiBH_4_ + MgH_2_ → 2LiH + MgB_2_ + 4H_2_ (47)

The reverse Reaction (47) can be regarded as the starting point for Li-RHC systems investigated by several groups [143,144]. Using high energy ball milling (HEBM), the particle size of Li-RHC could be reduced to 10 to 70 μm (15 m^2^/g, rounded-platelet morphology) [144]. Comprehensive kinetic modelling was performed by Neves et al. using the best-fitting models (JMAEK-Johnson-Mehl-AvramiErofeyev-Kholmogorov with n = 1 and n = 1.5; contracting area model, contracting volume model), revealing that the limiting step is the movement of the not-hydrogenated/hydrogenated material interface [144]. Further nanostructuring can be achieved by ball milling with additives producing active catalytic species such as Li_x_TiO_2_ and AlTi, which can hydrogenate within 30 min at 400 °C [144].

Polymeric scaffolds including the adaptive poly(4-methyl-1-pentene) (TPX™ Polymer) were employed as hosts for 2LiH + MgB_2_ + 7.5(3TiCl_3_·AlCl_3_) RHCs, and allowed for higher stability in hydrogen storage studies (variation ~0.005 wt% per cycle) [140]. When the C aerogel scaffold was used by Nielsen et al. for 2 LiBH_4_: MgH_2_ RHC, no toxic B_2_H_6_ emissions were detectable [142]. About 55% of the free pore volume of the scaffold was infiltrated by RHC, suggesting successive impregnation cycles could increase overall H_2_ wt% storage [142].

Gamba et al. utilized 2LiH + MgB_2_/2LiBH_4_ + MgH_2_ RHC for hydrogen purification under a H_2_–CO (0.1 mol%) mixture and CO methanation [143]. Inclusion of only 1 mol% TiO_2_ additive mechanical milling (2 h, planetary ball mill, 400 rpm, 0.1 MPa Ar) led to nanocomposites Li-RHC-Ti that afforded a stable 10.1 wt% hydrogen capacity after more than 10 a/d cycles [143].

The same composite 2LiH + MgB_2_/2LiBH_4_ + MgH_2_ RHC catalyzed by 5 mol% TiCl_3_ was synthesized by Neves et al. by high energy ball milling (5g powder, planetary ball-mill, 20 h, BPR 10:1, 230 rpm, 20% volume filling of vial). Thermodynamic measurements under absorption conditions led to the following parameters for the 1D-interface-controlled Reaction (47): ∆H = 34 ± 2 kJ∙mol^−1^ H_2_, ∆S = 70 ± 3 J∙K^−1^∙mol^−1^ H_2_, apparent activation energy E_a_ = 146 ± 3 kJ∙mol^−1^ H_2_ and A = (1.8 ± 1.0) 10^8^ s^−1^ [144].

The nanocomposite 2LiBH_4_-LiAlH_4_/RFC (RFC = resorcinol formaldehyde carbon aerogel) was obtained by a two-step melt-infiltration process (0.0595 cm^3^/g, 45.3 m^2^/g BET data confirm nanoconfinement inside pores) and showed good H_2_ storage properties with 5.7 reversible wt% [145]. AlB_2_ formed during the second dehydrogenation step altered the decomposition pathway of LiBH_4_, and incomplete rehydrogenation of LiBH_4_ was confirmed by the identification of Li_2_B_12_H_12_ in the FTIR spectrum (vibration peak at 2480 cm^−1^) [145].

Plerdsranoy et al. prepared a pellet of RHC compressed under 976 MPa (LiBH_4_-LiAlH_4_ with 1:1 molar ratio), which exhibited good mechanical stability during cycling with 80% of theoretical H_2_ capacity compared to the milled sample (65%) [146]. This improvement was also associated with an important reduction in activation energy ΔE_A_ = 69 kJ/mol H_2_ compared to in the milled sample. The support used was a carbonaceous polyacrylonitrile (PAN)-based activated carbon nanofiber ACNF, that was prepared by electrospinning, carbonization and chemical activation ((KOH_aq_). Solution impregnation (LiAlH_4_ 1 M in Et_2_O) and melt impregnation (LiBH_4_, 310 °C, 110 bar H_2_) took place with a 2:1 weight ratio ACNF: RHC (Reaction (48), 10.12 wt% theoretical H_2_ content for milled RHC and 3.37 wt% for RHC-ACNF).

LiAlH_4_ (l) + LiBH_4_ (l) → 2LiH (s) + 1/2AlB_2_(s) + 1/2Al (s) + 3H_2_ (g) (48)

Melt infiltration of 2LiBH_4_-NaAlH_4_ confined into CA (33% pore volume filling, 310 °C, 30 min, 110 bar H_2_) produced a 2.4 wt% reversibility. In fact, 9.52 wt% was released during the first dehydrogenation (3.36%, 1st step; 6.16 wt%, 2nd step), but the formation of the eutectic LiBH_4_-NaAlH_4_ at 250 °C was not associated with any H_2_ release [147]. Furthermore, it was shown that NaBH_4_ is the compound from the original RHC that stores H_2_ reversibly. The dehydrogenation reaction can be formulated according to (49).

2LiBH_4_(s) + NaAlH_4_(s) → [LiBH_4_(s) + LiAlH_4_(s) + NaBH4(s)] → 2LiH(s) + Na(s) + AlB_2_(s) + 5H_2_(g) (49)

A LMBH eutectic comprising LiBH_4_-Mg(BH_4_)_2_ in a 55:45 molar ratio was obtained by ball milling (BPR 40:1, 400 rpm, 2 h) [148]. High loading of LMBH in porous hollow carbon nanospheres (HCNS) by over-infiltration (33, 50 and 67 wt% at 190 °C, 60 bar H_2_, 1 h) led to LMBH@HCNS nanocomposites. It was observed that an interfacial adhesion effect due to HCNSs avoids borohydride aggregation during de/rehydrogenation, thus having a beneficial effect on cyclability. The optimal composition of the composite was 50LMBH@HCNS, with a 4.5 wt% stable reversible H_2_ capacity vs hydride content, or a 2.3 wt% actual cycling capacity (50 wt% LMBH loading in HCNS). The hydrogen release/uptake pathway is described by Reaction (50) for dehydrogenation, and by Reaction (51) for the rehydrogenation reaction [148].

LiBH_4_ + Mg(BH_4_)_2_ → MgB_2_ + LiH + B + 5.5H_2_ (50)

2LiH + MgB_2_ + 4H_2_ → 2LiBH_4_ + MgH_2_ (51)

An interesting strategy for the inclusion of 2LiBH_4_–MgH_2_ in a porous Ni/C scaffold was proposed by Huang et al. [149]. The host was produced by the pyrolysis of the nickel-based metal organic framework, MOF-74-Ni. The hydrogen capacity of the nanocomposite 2LiBH_4_–MgH_2_–15%Ni/C was ~9 wt% and MgNi_3_B_2_ was identified as an active catalyst for the hydrogenation reaction [149].

Dansirima et al. nanoconfined 2LiBH_4_-MgH_2_ into biomass-derived, activated carbon (AC) to produce LiBH_4_-MgH_2_-AC composite [150]. LiBH_4_-Mg (2:1 molar ratio) were milled (planetary ball mill, BPR 20:1, 10 h, 580 rpm), then milled in a 1:1 AC weight ratio to yield 2LiBH_4_-Mg-AC which underwent further hydrogenation of Mg (400 °C, 5 °C/min, 40–50 bar H_2_, 10 h), to yield the final composite: 2LiBH_4_-MgH_2_-AC. The H_2_ storing composite “LiBH_4_-MgH_2_-AC” was used in a small hydrogen storage tank (21.7 mL packing volume), proving the feasibility of using nanoconfined RHC as a viable fuel (58% of the 5.7 wt% theoretical hydrogen achieved: 3.28 wt%, due to temperature gradient and poor hydrogen diffusion through hydride bed). However, the formation of thermally stable Li_2_B_12_H_12_ limits the reversible H_2_ storage capacity, which conforms to the Reaction (51) [150].

The RHC 2LiBH_4_-MgH_2_ was produced by planetary ball milling (BPR 10:1, 5 h), and infiltrated in. ZrCl_4_–doped carbon aerogel scaffold CAS synthesized by the carbonization of cross-linked resorcinol-formaldehyde polymer [151]. The optimum loading was investigated by the melt infiltration technique, which produced composites RHC: ZrCl_4_-CAS with weight ratios 1:1 (pore blocking), 1:2 (most suitable) and 1:3 (lower H_2_ storage content). The optimum composition 1:2 featured 3.8 wt% theoretical H_2_ capacity, and showed 3.7 wt% -1st dehydrogenation, and 3.54–3.45 wt%—second to fourth dehydrogenation cycles. Partial dehydrogenation and formation of [B_12_H_12_]^2−^ seemed to the lower overall H_2_ storage capacity, which otherwise follows the pathway described by reactions (40) and (41) [151].

Dematteis et al. investigated mixed-cation borohydrides formed in ternary and quaternary systems: KCa(BH_4_)_3_, LiKMg(BH_4_)_4_, LiK(BH_4_)_2_ and also new eutectics. LiBH_4_ promoted early H_2_ release (~200 °C), while KCa(BH_4_)_3_ promoted a single-step reaction (higher temperature). The investigated ternary systems were LiBH_4_-NaBH_4_-Mg(BH_4_)_2_, LiBH_4_-NaBH_4_-Ca(BH_4_)_2_, LiBH_4_-KBH_4_-Mg(BH_4_)_2_, LiBH_4_-KBH_4_-Ca(BH_4_)_2_, LiBH_4_-Mg(BH_4_)_2_-Ca(BH_4_)_2_, NaBH_4_-KBH_4_-Mg(BH_4_)_2_, NaBH_4_-KBH_4_-Ca(BH_4_)_2_, NaBH_4_-Mg(BH_4_)_2_-Ca(BH_4_)_2_ and KBH_4_-Mg(BH_4_)_2_-Ca(BH_4_)_2_. Whereas, the quaternary systems comprise LiBH_4_-NaBH_4_-KBH_4_-Mg(BH_4_)_2_, LiBH_4_-NaBH_4_-KBH_4_-Ca(BH_4_)_2_, LiBH_4_-NaBH_4_-Mg(BH_4_)_2_-Ca(BH_4_)_2_, LiBH_4_-KBH_4_-Mg(BH_4_)_2_-Ca(BH_4_)_2_ and NaBH_4_-KBH_4_-Mg(BH_4_)_2_-Ca(BH_4_)_2_ (Table 3) [152].

An investigation of new mutually destabilized reactive hydride system LiBH_4_–Mg_2_NiH_4_. Mg_2_NiH_4_ and MgNi_2.5_B_2_ prepared in-house by Bergemann et al. revealed MgNi_2.5_B_2_ as an active intermediate, which was confirmed by ^11^B MAS NMR (154 ppm) [153]. MgO (<5 wt%) was present after dehydrogenation in the samples, which could be a result of the high affinity of Mg for oxygen, and points to unavoidable side reactions during sample manipulation. The dehydrogenation of RHC follows Reaction (42) which could hypothetically consist of three individual steps [153].

2LiBH_4_ + 2.5Mg_2_NiH_4_ → (2LiH + 2B +2.5Mg_2_Ni +8H_2_ → 2LiH + MgNi_2.5_B_2_ + 4Mg + 8H_2_) → 2LiH + MgNi_2.5_B_2_ + 4MgH_2_ + 4H_2_ (42)

A TiCl_4_-catalyzed CAS prepared by resorcinol-formaldehyde (RF) aerogels technique was used to yield composites 2LiBH_4_–MgH_2_–0.13TiCl_4_. The RHC was confined by solution impregnation and melt infiltration in nanoporous catalyzed carbon aerogel host TiCl_4_-CAS [154]. The superior behavior of TiCl_4_-catalyzed RHC was due to the formation of Ti–MgH_2_ alloys (Mg_0.25_Ti_0.75_H_2_ and Mg_6_TiH_2_) during the first rehydrogenation. The dehydrogenation proceeded in three steps (140, 240 and 380 °C), with no detectable traces of B_2_H_6_ [154].

Other investigations started from neat RHC LiBH_4_-NaBH_4_ which was synthesized by ball milling (BPR 30:1, 120 min, 350 rpm) leading to formation of a solid solution. Notably, the orthorhombic-to-hexagonal LiBH_4_ transition took place at 94 °C (15 °C lower than that of neat LiBH_4_). A new eutectic with a composition between Li_0.65_Na_0.35_BH_4_ and Li_0.70_Na_0.30_BH_4_ was observed (mp = 216 °C), and ab initio and Calphad allowed the calculation of thermodynamic parameters and the phase diagram [155].

Liu et al. synthesized LiBH_4_–Mg(BH_4_)_2_@NPC nanocomposites by melt infiltration (20 wt% hydride loading vs support; 200 °C, 60 bar H_2_, 30 min.) [156]. The mixed borohydride Li/Mg(BH_4_)_3_ formed an eutectic with a structure similar to α-Mg(BH_4_)_2_, with varying diborane (B_2_H_6_) and triborane (B_3_H*_8_*) evolution—lower for bulk, higher for nanoconfined, confirming a suppressed reaction pathway and altered decomposition mechanism [156].

The 0.62LiBH_4_-0.38NaBH_4_ RHC catalyzed by nano-Ni was prepared by planetary ball milling (10 h, 1 bar Ar, 175 rpm) [157]. Nano Ni-addition destabilizes the dehydrogenation process (a three-step reaction, 20–25 °C reduction in peak temperatures compared to neat RHC). The cycling stability was unaffected by the nickel catalyst, which facilitates regeneration of LiBH_4_. The dehydrogenation products were identified as Ni_4_B_3_ (first step) and Li_1.2_Ni_2.5_B_2_ (third step). The possible reactions of LiBH_4_ with Ni depend on the initial LiBH_4_:Ni molar ratio conforming to Equations (52)–(54), and comprise various nickel boride formulations as catalyst-containing species [157].

LiBH_4_ + 4/3Ni → 1/3Ni_4_B_3_ + LiH + 3/2H_2_ (52)

LiBH_4_ + 2Ni → Ni_2_B + LiH + 3/2H_2_ (53)

LiBH_4_ + 3Ni → Ni_3_B + LiH + 3/2H_2_ (54)

Additives M in the RHC of type (2LiBH_4_ + M) (M = LiAlH_4_, Li_3_AlH_6_) were utilized to destabilize LiBH_4_, lowering its mp to 445 °C and 435 °C, respectively [122]. The Li_3_AlH_6_ was synthesized from LiH and LiAlH_4_ by ball milling (50 h, 300 rpm, BPR 25:1), and was part of the most promising sample, the 2LiBH_4_-Li_3_AlH_6_ RHC, which featured a two-step release (3 and 6.1 wt% H_2_) forming AlB_2_ for a potentially reversible system [122].

The interfacial interaction of LiBH_4_ in LiBH_4_–Ca(BH_4_)_2_ RHCs (4:1, 2.1:1 -eutectic composition) with the surface of mesoporous silica (MCM-41 or SBA-15) revealed the liquid-like behavior starting at 95 °C when nanoconfined, which led to high diffusional mobility. Notably, utilizing (VT) NMR the authors revealed the co-infiltration of eutectic LiBH_4_–Ca(BH_4_)_2_ (hand or ball milling) into mesopores far below the eutectic melting point [158].

When introduced via mechanical milling, 2D-MXene (Ti_3_C_2_) has served as a support for 4MgH_2_-LiAlH_4_ RHC [159]. Chen et al. obtained the composite 4MgH_2_-LiAlH_4_-Ti_3_C_2_ which showed improved de/re-hydrogenation kinetics (ΔT = 64 K lower than milled 4MgH_2_-LiAlH_4_ RHC). Additionally, thermodynamic parameters for the three-stage dehydrogenation were also deduced (E_A_ = 65.9 kJ mol^−1^ H_2_^−1^, 70.6 kJ mol^−1^ H_2_^−1^ and 74.3 kJ mol^−1^ H_2_^−1^, respectively) [159].

The LiBH_4_/KBH_4_ mixed borohydride system was hand-ground and used as a eutectic electrolyte (0.725LiBH_4_/0.275KBH_4_; ~50% of whole RHC system was the hydride content) to facilitate dehydrogenation/rehydrogenation of theSn-catalyzed MgH_2_ and 0.5Sn system (ball milled; 2.3 wt% theoretical H_2_). Sn provides destabilization by the formation of Mg_2_Sn, thus lowering ΔH_dehydrogenation_ (Reaction (55)).

2MgH_2_ + Sn ↔ Mg_2_Sn + 2H_2_ (55)

Kinetic improvements account for a 10-fold increase in reaction rate of both de-and rehydrogenation. E_A_ was also decreased from ~150 kJ/mol to ~100 kJ/mol. Notably, the working regime imposed a restricted temperature profile, below mp (Sn) = 232 °C [160].

Using a Ti-based catalyst (TiF_3_, Ti and TiO_2_), Ma et al. showed the strong influence of titanium over dehydrogenation temperature and kinetics of the 4LiAlH_4_–Mg_2_NiH_4_ system [161]. A low activation energy of E_A, desorption_= 81.56 kJ mol^−1^ was deduced based on the Johnson-Mehl-Avrami model, and the most effective form of the catalyst was TiF_3_. Mg_2_NiH_4_ catalyzes the decomposition of LiAlH_4_, and it further accelerates dehydrogenation by reaction with in-situ formed Al (reactions (21), (22) and (56)).

Al + Mg_2_NiH_4_ → Mg_17_Al_12_ + Al_3_Ni + Al_1.1_Ni_0.9_ + H_2_ (220~330 °C) (56)

Mesoporous carbon (C-replica of SBA-15, namely CMK-3) was used as a host for the x LiBH_4_– (1 − x) Ca(BH_4_)_2_ system (x = 0.50, 0.60, 0.65, 0.68, 0.70, 0.75, and 0.8) [162]. The mixed borohydride was introduced in CMK-3 via melt infiltration (230 °C, 30 min, 3 bar H_2_) producing RHC@CMK-3 when mixed in a 1:1 weight ratio. Half of the initial dehydrogenation content was recovered after rehydrogenation, pointing to a synergy nanoconfinement-mutually-destabilized system. Analysis by XRD data of reaction products revealed Ca_3_(BH_4_)_3_(BO_3_) and LiCa_3_(BH_4_)(BO_3_)_2_ as a result of [BH_4_]^−^ oxidation, presumed to form outside the mesopores, due to unit cell size mismatch with a mesoporous channel diameter. The rehydrogenated material comprised LiCa_3_(BH_4_)(BO_3_)_2_ (ex situ XRD) [162].

Nanoconfinement of 0.55LiBH_4_–0.45Mg(BH_4_)_2_ system in activated carbon aerogel via melt infiltration was shown to facilitate both hydrogen release and uptake, while also maintaining a high H_2_ release content of 8.3 wt% in RHC@CA after the fourth hydrogen release [163]. A clear role in the high reversible H_2_ content was attributed to the high porosity and pore volume (689–2660 m^2^/g, 1.21–3.13 cm^3^/g) of the activated scaffold [163].

MWCNTs were utilized by Meethom et al. to confine LiBH_4_–LiAlH_4_ by ball milling [164]. The MWCNTs (5 wt%) provided enhanced thermal conductivity and a high surface area for reactive contact between Al and LiBH_4_/LiH, leading to a reversible in H_2_ storage with faster kinetics (three times faster) and reduced hydrogen release onset by ΔT = −120 °C. The quenching of the first desorption step (220 °C) was introduced for the first time, and among the reaction products, AlB_2_ was identified to form during reaction of LiAlH_4_ with Al (Equation (18)).

Other techniques like vacuum vapor deposition produced Mg@NaBH_4_/MgB_2_ core-shell structures affording ~6 wt% hydrogen storage [165]. Mg@NaBH_4_ displayed better hydrogenation properties to pristine Mg, while nanosized MgB_2_ produced NPs dispersed on Mg enhance sorption kinetics (E_A_ = 60.1 kJ/mol H_2_, ΔH_ab_ = −73.8 kJ/mol H_2_, ΔH_des._ = 89.7 kJ/mol H_2_, dehydrogenation onset of 245 °C was lower than for MgH_2_). The reaction mechanism confirms the intermediacy of MgH_2_-NaBH_4_ RHC, and conforms to Equation (57):

2MgH_2_ + 2NaBH_4_ → Mg + MgB_2_ + 6H_2_ + 2Na (57)

By ball milling, 4MgH_2_-LiAlH_4_-10 wt%TiO_2_ composite was obtained (1 h, 400 rpm, Ar) and showed that the TiO_2_ destabilized RHC-TiO_2_ compared to the neat RHC (lowered onset of the first step from 100 °C to 70 °C, and lowered the second step from 270 °C to 200 °C). The activation energy was also reduced from 133.3 (4MgH_2_-LiAlH_4_) to 102.5 kJ/mol (4MgH_2_-LiAlH_4_-TiO_2_) [166].

Composites LiBH_4_- x AlH_3_ (x = 0.5, 1.0, 2.0) introduced by Liu et al. [167] showed enhanced destabilization as [AlH_3_] increased in RHCs prepared by ball milling (proposed kinetic model: JMA) with E_A, RHC desorption_~122.0 kJ/mol, while E_A, LIBH4_ = 169.8 kJ/mol. Dehydrogenation seems to be controlled by AlB_2_ intermediate and its increased nucleation rate. The simple binary hydride AlH_3_ decomposes in the first step into H_2_ and Al, which is hypothesized to partake in the second dehydrogenation step (Reaction (58)). The nature of the “Li-Al-B” product was unclear [167].

LiBH_4_ + Al →’Li-Al-B’ + AlB_2_ + H_2_ (58)

The reactive mixture Mg(NH_2_)_2_–2LiH–0.07KOH was prepared by Chen et al. and yielded a new RHC containing Li_3_K(NH_2_)_4_–MgNH–LiNH_2_, with a decomposition dependent on H_2_ pressure [168]. The KOH role was highlighted in the Mg(NH_2_)_2_–2LiH RHC (90 °C theoretical desorption temperature from thermodynamic data, but ~220 °C in practice due to slow kinetics), which follows a reversible pathway described by (59):

(59) $${\mathrm{Mg}({\mathrm{NH}}_{2})}_{2}\;+2\mathrm{LiH}\;↔\;1/2{\mathrm{Li}}_{2}{\mathrm{Mg}}_{2}\;{\left(\mathrm{NH}\right)}_{3}\;+1/2{\mathrm{LiNH}}_{2}\;+1/2\mathrm{LiH}+3/2H_{2}\;↔\;{\mathrm{Li}}_{2}{\mathrm{Mg(NH)}}_{2}+2H_{2}$$

Reversibility and enhanced kinetics were reported for the RHC 2LiBH_4_–NaAlH_4_ melt infiltrated into CAS at 185 °C (mp_NaBH4_) and 310 °C(mp_LiBH4_), using weight ratios CAS:RHC of 2:1, 2:1.5 and 1:1 [169]. The composite 2:1 showed the best results storing ~2.4 wt% hydrogen up to 400 °C. Nanoconfinement by melt impregnation in CAS (resorcinol–formaldehyde aerogels) inhibits Na evaporation and promotes NaBH_4_ regeneration, which was impossible in as milled RHC due to the loss of Na source (Reaction (60)).

LiH + Al + 2NaH + 3/2H_2_ ↔ LiNa_2_AlH_6_ (60)

In a report by Peru et al. [170], a 5 nm pore-size CMK-3 carbon was utilized to infiltrate a eutectic mixture 0.725 LiBH_4_–0.275 KBH_4_ resulting in 2.5–3 wt% reversible hydrogen storage due to a favorable synergy C_surface_—nano-sized MBH_4_ (M = Li, K), which helped circumventing or reducing irreversible side-reactions [170].

More exotic borohydrides of lanthanides were also investigated. For instance, the mixture 3LiBH_4_ and Er(BH_4_)_3_ and 3LiH was shown to desorb up to 3.5 wt% after 3 a/d cycles [171]. LiBH_4_ was generated in-situ via metathesis, and its high temperature form (h-LiBH_4_) was stabilized in RHC at 40–60 K (61).

Er(BH_4_)3(s) + 3LiH(s) → ErH_2_(s) + 3LiBH_4_(s) + 0.5H_2_(g) (61)

The desorption (62)—resorption (63) are similar transformations, which also imply a redox behavior of the Er(II/III) center [171].

4LiBH_4_(s) + ErH_2_(s) → ErB_4_(s) + 4LiH(s) + 7H_2_(g) (62)

ErB_4_(s) + 4LiH(s) + 7.5H_2_(g) → 4LiBH_4_(s) + ErH_3_(s) (63)

When confined in activated carbon nanofiber ACNFs, the 2LiBH_4_-MgH_2_ RHC produced 2LiBH_4_-MgH_2_ -30 wt% ACNFs composites storing up to 4.5 wt% hydrogen [172]. The dehydrogenation onset was reduced by 50 °C (from 350 °C to 300 °C) due to doping with ACNFs and subsequent compaction to pellet (under 600 MPa). The activation energy E_A_ was reduced by 67 kJ/mol, and H_2_ permeability was increased in compacted RHC@ACNFs (10 times), while the heat transfer was also increased (1.5 times). ACNFs alter the dehydrogenation pathway, from a one-step process (neat RHC: 2LiBH_4_-MgH_2_) to a two-step process (RHC@ACNFs). H_2_ desorbs from both individual hydrides (yielding Mg and LiH), rather than reacting according to the neat 2LiBH_4_-MgH_2_ (Equations (40) and (41)) [172].

The RHC K_2_Mn(NH_2_)_4_–8LiH was recently investigated [173]. K_2_Mn(NH_2_)_4_ was synthesized by ball milling metallic 1 Mn: 2 K under seven bar NH_3_, and activated by ball milling (12 h, 200 rpm, BPR 40:1, 10 bar H_2_). Final dehydrogenation products contain Li_2_NH, Mn_3_N_2_ and MnN, while rehydrogenation mixture contains LiH, LiNH_2_ and other “K-Mn-species”. Notably, the activation energy E_A_ = 65 kJ/mol (K_2_Mn(NH_2_)_4_–8LiH) is comparable with E_A_ = 66 kJ/mol for the more mainstream RHC: LiNH_2_-2LiH [173].

Javadian et al. utilized the eutectic mixture 0.62LiBH_4_–0.38NaBH_4_produced by melt infiltration composites RHC@CA (CA = CO_2_-activated resorcinol-formaldehyde carbon aerogel host with 37–38 nm pores and S_BET_ = 690–2358 m^2^/g) [174]. The authors recorded important thermodynamic enhancement (ΔT = −100 °C of nanoconfined vs. neat RHC), while cycling capacity was three times higher. The activation energy E_A_ was decreased from 139 kJ/mol (bulk) to 116–118 kJ/mol (C aerogel nanoconfined), with 4.3 wt% hydrogen storage (0.7 of initial) after four a/d cycles. By contrast, the overall reversibility of neat 0.62LiBH_4_–0.38NaBH_4_ was poor with 1.6 wt% (0.22 of theoretical) after four a/d cycles [174]. Another example of an eutectic mixture of borohydride comes from the same group, when 0.7LiBH_4_–0.3Ca(BH_4_)_2_ was infiltrated into activated carbon aerogel (pristine: 689 m^2^/g, 1.21 cm^3^/g and CO_2_-activated: 2660 m^2^/g, 3.13 cm^3^/g) filling the pores up to 60 vol% [175]. Thermodynamic improvements recorded are a clear reduction of dehydrogenation temperature ΔT = −83 °C (CA-RHC; 156 kJ/mol) and ΔT = −95 °C (CA_CO2_-RHC, E_A_ = 130 kJ/mol) compared to neat RHC (E_A_ = 204 kJ/mol). CO_2_-activated CA produces less borates and oxides, highlighting the narrow pore’s role in maintaining stability and altering the thermodynamic pathway in RHCs. Reversible behavior is reasonably well described by Reaction (64).

4LiBH_4_ + Ca(BH_4_)_2_ ↔ 4LiH + CaB_6_ + 10H_2_ (64)

Taking advantage of the investigation possibilities offered when labelled borohydrides are used, in 2012 Sartori et al. described the melt infiltration of Li^11^BD_4_–Mg(^11^BD_4_)_2_ into carbon nanoscaffolds (IRH33, 1.17 cm^3^g^−1^, 2587 m^2^ g^−1^_,_ 0.5 to 4.5 nm pores) yielding Li^11^BD_4_–Mg(^11^BD_4_*)*_2_/IRH33 composites (190 °C, 40 bar D_2_, 1 h) [176]. By-products (like the toxic B_2_D_6_) and poorly regenerative dodecaborane and [B_12_D_12_]^2−^ were avoided by nanoconfinement (no signals in the expected region of ^11^B NMR), while also reducing the desorption temperature by ΔT = −60 °C.

A proof of thermodynamic destabilization of NaBH_4_ by Li_3_AlH_6_ was shown in NaBH_4_–Li_3_AlH_6_ composite [132]. Na, Al and AlB_2_ species formed during dehydrogenation were key to the observed improvement. The activation energies recorded were reduced to E_A,1_ = 162.1 kJ/mol and E_A,2_ = 68.1 kJ/mol for NaBH_4_ decomposition [132].

The predicted hydrogen release in RHC do not usually match actual experimental data. For instance, although predicted to release 4.2 wt% H_2_ at 64 °C and 1 bar H_2_, the 6Mg(NH_2_)_2_–9LiH–LiBH_4_ RHC faces kinetic barriers, and YCl_3_/Li_3_N additives were used to alter the kinetics [177].

Other TM salt additives were used in more widespread RHC. The 2LiH and MgB_2_/2LiBH_4_ + MgH_2_ system was catalyzed with 0.05 TiCl_3_ and used as an additive [144]. The following thermodynamic parameters were determined: ΔH = −34 ± 2 kJ∙mol H_2_^−1^ and entropy ΔS = −70 ± 3 J∙K^−1^∙mol H_2_^−1^_;_ while the apparent E_A_= 146 ± 3 kJ∙mol H_2_^−1^ and the Arrhenius pre–exponential factor was A= (1.8 ± 1.0) 10^8^ s^−1^ [144].

Another report of using TiCl_3_ for the 2LiBH_4_–MgH_2_ RHC’s inclusion in the porosity of resorcinol–formaldehyde carbon aerogel scaffold (RF–CAS) comes from Gosalawit-Utke et al. [178]. The carbon scaffold RF-CAS was decorated with TiCl_3_ (1.6 wt%) by solution impregnation, and after melt impregnation of RHC produced composites 2LiBH_4_–MgH_2_–TiCl_3_@RF-CAS capable to release 3.6 wt% hydrogen during the fourth a/d cycle [178]. The kinetics were twice as fast relative to undoped RHC, due to TiCl_3_ doping. The 2LiBH_4_–MgH_2_–TiCl_3_ system proved to be reversible, and regeneration of LiBH_4_ and MgH_2_ was assessed by FTIR and SR-PXD data, overall conforming to the Equations (40) and (41).

**Figure 5:**
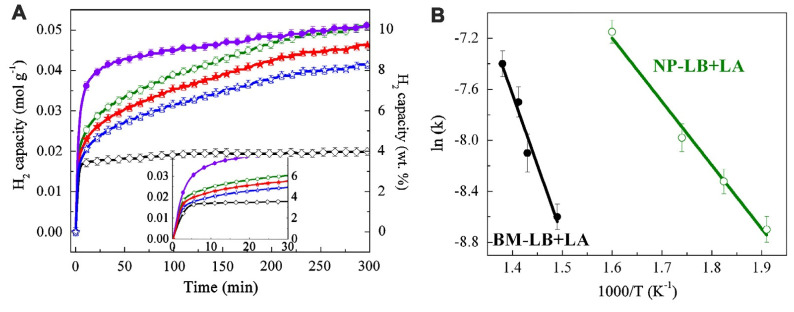
(**A**) Isothermal H_2_ release of the nanoconfined 2LiBH_4_–LiAlH_4_ composite at 250 C (blue), 275 C (red), 300 C (green) and 350 C (violet), including the post-milled 2LiBH_4_–LiAlH_4_ composite at 350 C (}) for comparison. The inset is an enlargement of (**A**) for dehydrogenation time from 0 to 30 min. (**B**) Arrhenius plots of the temperature-dependent rate data for the post-milled and nanoconfined 2LiBH_4_–LiAlH_4_ composites. Figure is re printed with permission from ref. [179].

The reversible behavior of the 2LiBH_4_-LiAlH_4_ system was assessed by nanoconfinement in mesoporous carbon (MC) scaffolds, with ~8.5 wt% reversibility confirmed by seven a/d cycles (Figure 5) [179].

**Figure 6:**
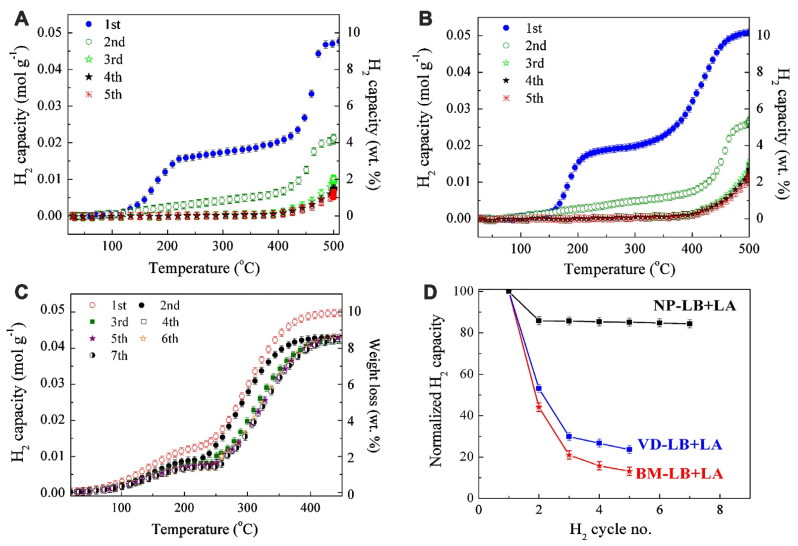
Evolution of consecutive H_2_ desorption curves: five cycles for the ball milled (**A**) and vacuum-dried (**B**) composites; (**C**) seven cycles for the nanoconfined 2LiBH_4_–LiAlH_4_ composite and (**D**) normalized H_2_ capacity as a function of cycle number for the post milled, vacuum-dried and nanoconfined 2LiBH_4_–LiAlH_4_ composites, respectively. Figure is reprinted with permission from ref. [179].

The dehydrogenation events in 2LiBH_4_-LiAlH_4_@MC were identified at 80 °C (ΔT = −40 °C) and 230 °C (ΔT = −145 °C), considerably lower than the milled 2LiBH_4_-LiAlH_4_. The favorable synergy nanoconfinement–thermodynamic destabilization suppressed B_2_H_6_ emissions, forming catalytically active AlB_2_ instead of Li_2_B_12_H_12_. The decomposition conforms to Equation (65) (Figure 6) [179].

2LiBH_4_ + LiAlH_4_ ↔ 3LiH + AlB_2_ + 9/2H_2_ (65)

As oftentimes seen in XRD diffractogram, metal borides arise as potential intermediates in dehydrogenation studies. Hence, their effect was checked by independently preparing Fe_3_B–catalyzed LiBH_4_-MgH_2_ RHCs soring ~2.9 wt% hydrogen after the seventh a/d cycle, with the main release step occurring below 265 °C [180]. A new processing method, Ball Milling with Aerosol Spraying (BMAS) was introduced by Ding et al., producing Mg(BH_4_)_2_ at RT from (nano-) MgH_2_ and LiBH_4_ [180].

Other investigations expanded on the multi-borohydride systems with an example of quinary RHC, namely LiBH_4_-NaBH_4_-KBH_4_-Mg(BH_4_)_2_-Ca(BH_4_)_2_ [181]. The equimolar composition of complex borohydrides was produced by a planetary ball mill (10 bar H_2_, 10 mm diameter stainless steel balls and BPR 30:1, 1–50 h, 350 rpm), yet even after 50 h of milling no miscibility was obtained. The only phase formed upon milling was KCa(BH_4_)_3_, while dehydrogenation occurred from the liquid phase as a complex multi-step process (in-situ SR PXRD, Synchrotron Radiation Powder X-ray Diffraction). This was the first report of five-component liquid borohydride obtained via the eutectic approach [181].

Another eutectic mixture 0.68LiBH_4_–0.32Ca(BH_4_)_2_ (“LiCa” eutectic) was nanoconfined in the porosity of a carbon aerogel scaffold CAS (*S*_BET_ = 2421 ± 189 m^2^/g, *V*_tot_ = 2.46 ± 0.46 mL/g, 13 nm pore size) [182]. The as-synthesized nanocomposite had a maximum theoretical H_2_ capacity of 14.34 wt%, when Reaction (64)) occurred via melt infiltration. It seemed that the LiBH_4_ component was affected only during the first a/d cycle by CAS confinement (ΔT = −40 °C), with diminishing returns after four cycles (ΔT = −10 °C). LiBH_4_@CAS continuously loses H_2_ capacity after the second cycle, but “LiCa”@CAS and neat “LiCa” retain stability during cycling up to the seventh cycle. Ca(BH_4_)_2_ decomposition products (CaO, CaH_2_ and/or CaB_6_) are believed to facilitate full LiBH_4_ reversibility [182].

Due to its high gravimetric capacity, Ca(BH_4_)_2_ was investigated in other RHCs as well, for instance in 2NaAlH_4_ + Ca(BH_4_)_2_ with 5 wt% TiF_3_ as additive [183]. Mustafa et al. described TiF_3_-doped RHC (ball milling, 6 h, 400 rpm, BPR 40:1), converting the initial RHC_i_: NaAlH_4_–Ca(BH_4_)_2_ system into RHC_f_: Ca(AlH_4_)_2_–NaBH_4_ (Reaction (44)). The intermediate phases in (2NaAlH_4_-Ca(BH_4_)_2_)-to-(Ca(AlH_4_)_2_-NaBH_4_) transition were identified as CaAlH_5_ (Reaction (66)) and CaH_2_ (Reaction (67)).

Ca(AlH_4_)_2_ → CaAlH_5_ + Al + 3/2H_2_ (66)

3Ca(AlH_4_)_2_ + 2TiF_3_ → 3CaF_2_ + 2Al_3_Ti + 12H_2_ (67)

The a/d of RHC-TiF_3_ was significantly improved by TiF_3_, with a significantly decreased desorption onset (125 °C to 60 °C for 1st stage), and corresponding similar reduction of activation energies of CaAlH_5_ (79.3 kJ/mol; ΔE_A_ = −63.6 kJ/mol) and NaBH_4_ (124.6 kJ/mol; ΔE_A_ = −21.9 kJ/mol). The complex hydride CaAlH_5_ produced in (66) would decompose to generate CaH_2_ and Al (Reaction (68)).

CaAlH_5_ → CaH_2_ + Al + 3/2H_2_ (68)

Notably, CaH_2_ plays a critical role because it will generate Al-Ca alloys by reaction with Al (Reaction (69)).

CaH_2_ + 3Al → 1/2Al_4_Ca + 1/2Al_2_Ca + H_2_ (69)

The two formulations of Al-CA alloys further react with the more thermodynamically stable NaBH_4_ (Reactions (70) and (71)), again producing active boride species (CaB_6_, AlB_2_).

14NaBH_4_ + Al_4_Ca → 14Na + CaB_6_ + 4AlB_2_ + 28H_2_ (70)

10NaBH_4_ + Al_2_Ca → 10Na + CaB_6_ + 2AlB_2_ + 20H_2_ (71)

**Figure 7:**
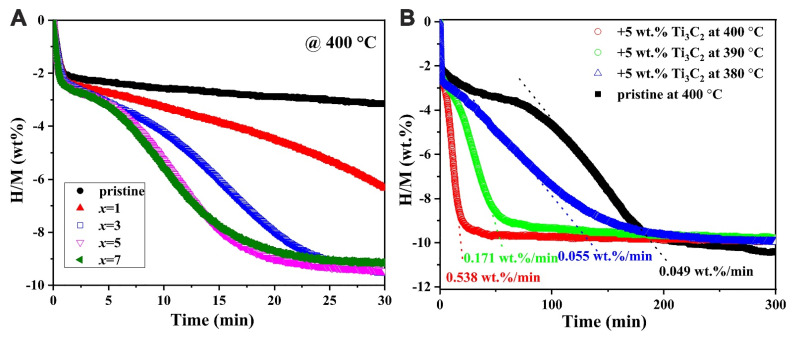
(**A**) Isothermal dehydrogenation curves (400 °C, 0.4 MPa H_2_) of initially hydrogenated 2LiH and MgB_2_ and x wt% Ti_3_C_2_ systems; (**B**) Isothermal dehydrogenation curves at different temperatures of the systems with 5 wt% Ti_3_C_2_ addition and without Ti_3_C_2_. Figure is reprinted with permission from ref. [184].

When 2D materials of the MXene type were introduced by ball milling as nanoadditives (1, 3, 5 and 7 wt%) in the 2LiH and MgB_2_ system, RHCs 2LiBH_4_ and MgH_2_ were produced [184]. The source of MgH_2_ regeneration was discussed by the authors in the context of TiB_2_ formed during dehydrogenation, which functions as heterogeneous nucleation nuclei for MgB_2_, allowing the control over nanosizing of the system during cycling. Another interesting feature of the system was the observed reduction of MXene Ti_3_C_2_ to generate Ti(0)) active metal sites, a crucial event for generating the catalytic TiB_2_ species (Reaction (72)) (Figure 7) [184].

Ti + 2LiBH_4_ → TiB_2_ + 2LiH + 3H_2_ (72)

A ternary RHC system LiBH_4_-MgH_2_-NaAlH_4_ was introduced by Plerdsranoy in 2016 and transformed in another RHC LiAlH_4_–MgH_2_–NaBH_4_ when nanoconfined by melt infiltration in CAS (resorcinol-formaldehyde synthesis; CAS-RHC 1:1 weight ratio) [185]. This led to thermodynamic improvement (ΔT = −70 °C) and reversible behavior after four cycles (65% and 55% H_2_ released by nanocomposites). Notably, thermodynamic alteration by nanoconfinement reduced the multi-step release for neat RHC to only one major H_2_ release event. NaBH_4_ starts to decompose at 360 °C (@CAS) vs. 475 °C (milled RHC) or 540 °C (bulk NaBH_4_). Re-hydrogenation proceeds also at 360 °C, under 50 bar H_2_ for 12 h, making NaBH_4_, MgH_2_ and Li_3_AlH_6_ reversible, but Li_3_AlH_6_ and NaBH_4_ were partially reversible when nanoconfined (p_rehydrogenation_ > 80 bar H_2_). A downside when dealing with fully reduced light metals, is that the production of Na(l) is followed by its evaporation and the subsequently reduced H_2_ wt% capacity (Reactions (21), (22) and (73)) [185].

NaAlH_4_ + LiBH_4_ → LiAlH_4_ + NaBH_4_ (73)

Intermediate phases form as a result of the reaction of MgH_2_-Al (46) and MgH_2_—LiH (74).

7MgH_2_ + 3LiH → Li_3_Mg_7_ + 8.5H_2_ (74)

There are some reports of less-common complex hydrides as components of innovative RHCs, like the (1 − x)LiBH_4_ − x Mg_2_FeH_6_ system (x = 0.25, 0.5, 0.75), releasing 6.0 wt% hydrogen up to 630K [186]. Pressure-composition-isothermal (PCT) data revealed the same reaction occurring within all investigated samples (x = 0.5). The reversibility was demonstrated for equimolar RHC (LiBH_4_ − Mg_2_FeH_6_) after 4 a/d cycles, with no H_2_ wt% loss. The dehydrogenation reaction conforms to Reaction (75) (ΔH = 64 kJ/mol H_2_; ΔS = 125 J/(K molH_2_)), and confirms the crucial role of FeB [186].

LiBH_4_ + Mg_2_FeH_6_ → LiH + 2MgH_2_ + (Fe,FeB) + 5/2H_2_ (75)

Another experimental data was the equilibrium pressure of 1.85 MPa, very close to the theoretical one (2.1 MPa, 643 K—from thermodynamic considerations). Mg resulted from MgH_2_ dehydrogenation could react with LiBH_4_ (Reaction (15)), and the final H_2_ release step was assigned to Mg_2_FeH_6_ dehydrogenation (Reaction (76)).

Mg_2_FeH_6_ → 2Mg + Fe + 3H_2_ (76)

Lastly, reversible capacity of 5.0 wt% was recorded in NaAlH_4_-7 wt%NP-TiH_2_@G composites prepared by using reactive titanium (II) hydride–catalyzed graphene nanosheets [187]. The reactive TiH_2_ was prepared by ultrasonication (4 h, 40 kHz) of TiCl_4_ with LiH in THF (handled in an Ar-filled glovebox; Reaction (77)).

TiCl_4_ + 4LiH⟶TiH_2_ + 4LiCl + H_2_↑ (77)

**Figure 8:**
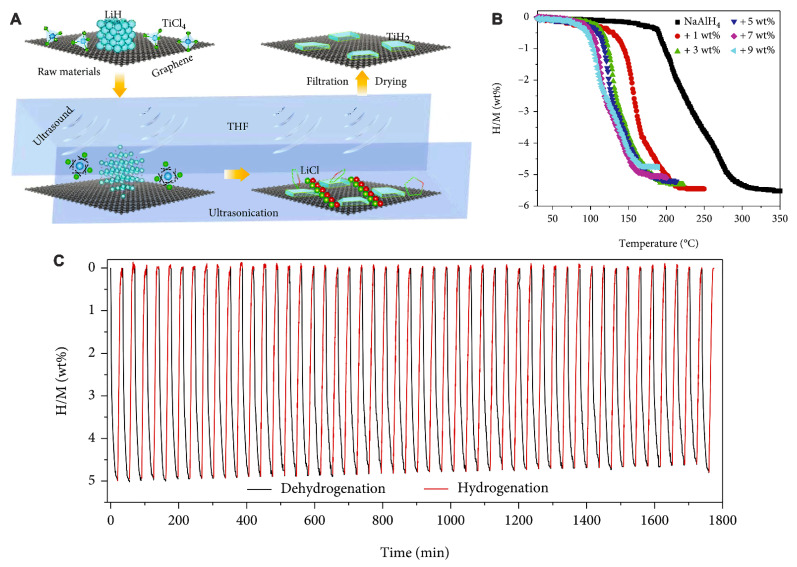
(**A**) Schematic illustration for the preparation process of TiH_2_ nanoplates.; (**B**) Volumetric release curves of NaAlH_4_ doped with NP-TiH_2_@G, (**C**) Cycling tests operated at 140 °C for dehydrogenation and 100 °C/100 atm H_2_ for hydrogenation of NaAlH_4_-7 wt% NP-TiH_2_@G. Figure is reprinted with permission from ref. [187]-Copyright © 2021 Zhuanghe Ren et al. Exclusive Licensee Science and Technology Review Publishing House. Distributed under a Creative Commons Attribution License (CC BY 4.0).

The nanocomposite NaAlH_4_-7 wt% NP-TiH_2_@G resulted after incorporation of NaAlH_4_ into NP-TiH_2_@G scaffolds, could be fully dehydrogenated very close to RT, at 30 °C. The dehydrogenation occurred in two steps with thermodynamically accessible energy barriers (E_A,1_ = 80 ± 3*:*3 kJ/mol and E_A,2_ = 70 ± 2*:*8 kJ/mol), probably lowered by formation of reactive, catalytic Al-Ti species (Figure 8) [187].

A summary of the main aspects discussed on nanosized RHCs is given in Table 5.

#### 2.1.6. LiBH_4_-Adducts—Ammoniates

Ammine metal borohydrides of the general formula M(BH_4_)_n_ · *x*NH_3_ are particular cases of metal borohydride adducts M(BH_4_)_n_ · *x* L (L = dative ligand, typically containing an electron-donor atom/group). While providing good H_2_ storage capacity, the main drawback of borohydride adducts is the lack of a generally applicable regeneration treatment.

In case of ammonia-adduct LiBH_4_·NH_3_, a three-step process was proposed, involving digestion (H^+^ addition to Li-B-N polymer formed by dehydrogenation; CH_3_OH treatment producing LiB(OCH_3_)_4_ depicted by Reaction (78)), reduction (H^−^ addition; LiAlH_4_ converts LiB(OCH_3_)_4_ into LiAl (OCH_3_)_4_ and regenerated LiBH_4_) and NH_3_-complexation (exposure of LiBH_4_ to ammonia NH_3_ atmosphere) [188].

LiN_x_BH_y_ + CH_3_OH ⟶ LiB(OCH_3_)_4_ + xNH_3_ + yH_2_ (78)

**Figure 9:**
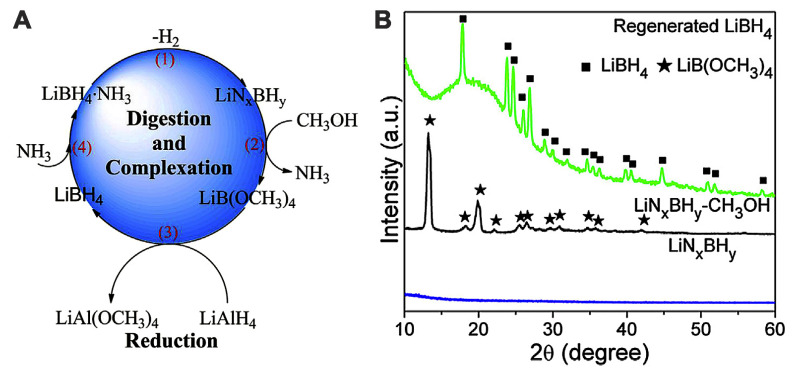
(**A**) Regeneration of ammonia adduct LiBH_4_·NH_3_ from spent fuel LiN_x_BH_y_ constituting a multi-step process: dehydrogenation (1), digestion (2), reduction (3) and ammonia complexation (4). (**B**) XRD patterns of the products of LiNxBHy (0 < x < 1, 0 < y < 1) after alcoholysis and then reduction by LiAlH_4_. Reprinted/edited with permission from ref. [188].

In the process, a CoCl_2_ catalyst was also used, yielding LiBH_4_ · NH_3_-2 mol% CoCl_2_ composite which was released upon heating to 200° (2 °C/min) ~13.6 wt% H_2_ (3 equiv.), thus producing LiN_x_BH_y_. Regeneration of ammonia adduct of LiBH_4_ from spent fuel LiN_x_BH_y_ was achieved through the three-step process (MeOH-LiAlH_4_-NH_3_ method) and was confirmed by ^11^B NMR and XRD data (Figure 9) [188].

**Figure 10:**
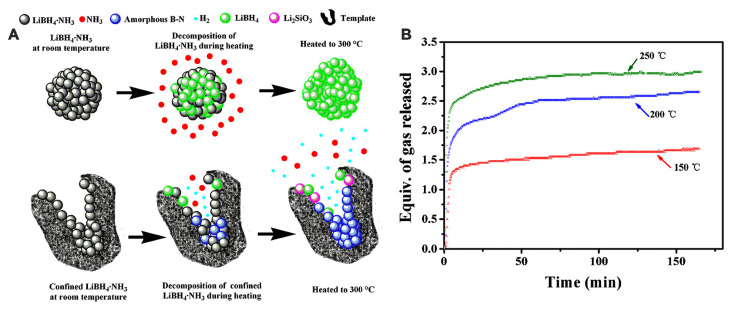
(**A**) Schematic model of LiBH_4_.NH_3_ and confined LiBH_4_.NH_3_@SiO_2_, which displays the difference in their thermal decomposition. (**B**) The isothermal gas releases results on the confined LiBH_4_.NH_3_@SiO_2_ (1:2, wt/wt) sample at 150 °C, 200 °C and 250 °C. Figure is reprinted with permission from ref. [189].

The same ammoniate complex of LiBH_4_ was also investigated by nanoconfinement in nanoporous SiO_2_ (LiBH_4_·NH_3_@SiO_2_, 1:2 wt/wt)) [189]. This strategy led to an onset dehydrogenation reaction at 60 °C, and an enhanced conversion of NH_3_ to H_2_ (85% of total gas evolved). The H_2_ release was measured to be 1.26, 2.09 and 2.35 equivalent of hydrogen at 150 °C, 200 °C, and 250 °C, respectively. It was proposed that the new “ammonia-deliquescence” method—which avoids the usage of additional solvents—affords a low dehydrogenation temperature due to the NH_3_ interaction with siloxanic support, which leads to the stabilization of ammonia by nanoconfinement into nanopores, and enhanced interaction of LiBH_4_ and NH_3_ in the nanoscaffold (Figure 10) [189].

#### 2.1.7. LiNH_2_BH_3_ (Lithium Amidoborane)

Lithium amidoborane (LiNH_2_BH_3_) is a promising material for hydrogen storage due to its high H_2_ content (10.9 wt%), which can be desorbed below 100 °C without formation of side-products like borazine. It can be synthesized by reaction of ammonia borane (NH_3_BH_3_) with either LiH or Li_2_NH (79).

(79) $$\mathrm{LiH}+{\mathrm{NH}}_{3}{\mathrm{BH}}_{3}\;\overset{-H_{2}}{\to }\;{\mathrm{LiNH}}_{2}{\mathrm{BH}}_{3}\;\overset{-\frac{1}{2}{\mathrm{NH}}_{3}}{\leftarrow }½{\mathrm{Li}}_{2}\mathrm{NH}+{\mathrm{NH}}_{3}{\mathrm{BH}}_{3}$$

Bearing similarities to NH_3_BH_3_, lithium amidoborane will release H_2_ in two consecutive steps (90 °C, and 150 °C), yielding LiNHBH_2_ and finally, LiNBH (80).

(80) $${\mathrm{LiNH}}_{2}{\mathrm{BH}}_{3}\;\overset{-H_{2}}{\to }\;{\mathrm{LiNHBH}}_{2}\;\overset{-H_{2}}{\to }\;\mathrm{LiNBH}+H_{2}$$

Ammoniates of LiNH_2_BH_3_ are also known, and LiNH_2_BH_3_.NH_3_ can generate H_2_ at very reasonable temperature ranges ~40–70 °C. Regeneration of lithium amidoborane can be achieved by reaction of solid residue LiNBH with methanol with the formation of LiB(OCH_3_)_4_, and distillation to form B(OCH_3_)_3_ which can be reduced by LiAlH_4_ and NH_4_Cl to NH_3_BH_3_ (AB). As a last step, reaction of AB with LiH reforms LiNH_2_BH_3_ [190]. Even under a nanoconfined state, LiNH_2_BH_3_ has an exothermic decomposition and cannot be regenerated under 10 MPa H_2_ pressure [168].

#### 2.1.8. Li-N-H System; Li_3_BN_3_H_10_

The Li-N-H system has received attention due to important thermodynamic features which allured the community into the promise of a material that would release H_2_ at temperatures near ambient condition. However, as is often the case with many hydrogen storage systems, its reversibility is not trivial [136]. As previously mentioned, RHC typically contain an amide source and a hydride partner, oftentimes with the aid of a third component (complex hydride—borohydride or catalyst), as is the case in the 6Mg(NH_2_)_2_-9LiH-LiBH_4_/(YCl/Li_3_N) system discussed before [177].

**Figure 11:**
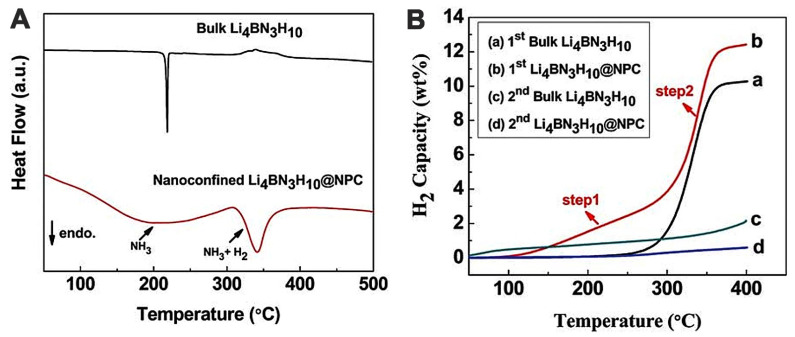
(**A**) DSC curves of bulk Li4BN3H10 and nanoconfined Li_4_BN_3_H_10_@NPC. The heating rate is 5 C/min. (**B**) Temperature-programmed desorption (TPD) curves of as prepared Li_4_BN_3_H_10_ and pre-melted Li_4_BN_3_H_10_@NPC with an initial loading of 20 wt%. (a) First desorption of prepared Li_4_BN_3_H_10_; (b) First desorption of pre-melted Li_4_BN_3_H_10_@NPC; (c) Second desorption of rehydrided Li_4_BN_3_H_10_; (d) Second desorption of rehydrided Li_4_BN_3_H_10_@NPC. Note that some of the desorbed gas is NH_3_, especially in the bulk sample (a). Figure is reprinted with permission from ref. [191].

An interesting report of an Li-N-H system involved nanoconfinement of Li_3_BN_3_H_10_ in the pores (4.4 nm) of highly ordered nanoporous carbon NPC (S_BET_ = 1012 m^2^/g, V_pore, BJH_ = 0.65 cm^3^/g). Thermodynamic destabilization was confirmed by an onset dehydrogenation temperature of 110 °C (160 °C lower than bulk). More importantly, the exothermic reaction of bulk Li_4_BN_3_H_10_ was altered to a two-step, endothermic process which released lower toxic gases—NH_3_ and B_2_H_6_—upon confinement (Figure 11) [191].

The sheer size of the mesopores (4.4 nm) appeared to play an important role in driving decomposition and suppressing B_2_H_6_ emission, while other reports utilizing mesoporous carbon of larger sizes (13 nm) did not bypass this issue [191].

### 2.2. Na-Based Complex Hydrides

#### 2.2.1. NaBH_4_

NaBH_4_ bears similarities with its lighter counterpart (LiBH_4_); however, its thermodynamic stability is too high for use in hydrogen storage materials in the bulk form (580 °C, 1 atm H_2_, E_A_ = 275 kJ/mol). Catalysts and nanostructuring have shown very promising results in the recent past, achieving system destabilization and implicitly lower dehydrogenation temperatures and, in some case, partial reversibility [192], by controlling particle size growth during cycling [193].

Sodium borohydride remains an important pillar of the hydrogen storage puzzle, whether used in RHC with other borohydrides [152,156,174,181], or with other complex hydride systems (alanates [132] or Mg_2_NiH_4_ [194]), nanostructured [192], on development of new synthesis methods to be obtained in NP form [60,195], pursuing features like restrained growth [193], fast Li-ion conductors [66], catalyzed systems (GdF_3_ [196,197], ScF_3_-YF_3_ [198], V-based catalysts [199], SiS_2_ [103], MgFe_2_O_4_ [200]), inclusion in core-shell-structures (with Mg/MgB_2_ as partners) [165], or investigation accounts based on physical methods investigation of {BH_4_]^−^ anion mobility (neutron scattering [71]) or interphase evolution (LiI [201]). Other approaches have employed the use of FeCl_3_ catalyst for NaBH_4_-based proton exchange membrane fuel cells [202].

#### 2.2.2. NaAlH_4_

Sodium aluminate (NaAlH_4_) is another promising complex hydride which promises a theoretical H_2_ storage capacity of 5.5 wt% at 250 °C, which—at least on paper—complies to US DOE’s upcoming target for the year 2025 [78,113,203]. However, regeneration is still an issue and can be aided by catalyst/dopant addition, or by nanostructuring techniques [110,111,204] which oftentimes alter the hydrogen dynamics [205], ionic conductivity [206,207] and thermodynamic behavior of alanate systems [114,208].

Recent reports focus on confinement in carbonaceous hosts: in ordered mesoporous carbon [209,210], ScOCl-functionalized carbon aerogel [211], MOFs (Ti-functionalized MOF(Mg) [212], new additives or scaffolds (Ni Raney with pore size 3 nm –affording onset of desorption at ~85° [213], graphene G [214], Ti-doped CO_2_-activated carbon aerogel [215], polymer nanocomposites based on g of polyaniline or sulfonated polyetherimide as polymer matrices, affording 1.1 wt% storage after 12 h at 120 °C and 32 bar H_2_ [216], N-doped nanoporous carbon NPC that lowered desorption E_A_ by 70 kJ/mol [217], graphene oxide GO [218], Al [219], exploring plasmonic heating effect on Au–hydride interface for local, light-activated heating [220], Co-catalyzed porous carbon hosts–affording a 3.3 wt% reversible gravimetric capacity after five cycles a/d [221], carbon nanotubes CNTs [222], CeO_2_ hollow nanotubes HNTs [223], ordered mesoporous carbon OMC–producing a highly stable material with 80% H_2_ capacity retention after 15 cycles [224], CeF_3_/Ti_3_C_2_ MXene [225], TiH_2_/G [187], C@TiO_2_/Ti_3_C_2_ [226]). In addition, novel investigations on RHCs (NaAlH_4_-Ca(BH_4_)_2_ [183]), undoped– [227] or TiCl_3_-catalyzed micro-mesoporous carbon produced by resorcinol-formaldehyde method [228], multi-wall carbon nanotubes MWCNTs [229], Ni-nanoporous sheets of carbon [125], Ti-functionalized MOF–74(Mg) [212] or improved synthetic methods that generate alanates as NPs [59] were focused on. Destabilization of NaBH_4_ was reviewed recently by correlation to TM fluorides used as dopants [230]. Destabilization can also be achieved using an ionic liquid–vinylbenzyl trimethylammonium chloride [231], alkali metals addition –leading to enhanced reversibility [232] or carefully-chosen TM ferrites like NiFe_2_O_4_ NPs [233]. Using RHC has proved advantageous, especially when combined with light metal borohydrides (2LiBH_4_-NaAlH_4_ [169], quinary equimolar mixture of light borohydrides [181] or various eutectic compositions in Al nano-framework [234]).

### 2.3. Mg-Based Complex Hydrides

Magnesium based hydrides have puzzled scientists because they offered remarkable H_2_ storage properties largely unattainable by other complex or simple hydrides, including the real prospect of reversibility during cycling [235,236,237]. Results stemmed from the high energy ball milling process [238], magnesium borohydride [239], or advances in the Li-Mg-Al systems [240].

#### 2.3.1. Mg(BH_4_)_2_

Perhaps one of the most fascinating borohydrides, Mg(BH_4_)_2_, has the largest number of polymorphs (at least six of them characterized), complex crystal structures and true permanent porosity (γ-polymorph, ~1100 m^2^/g surface area), all the while exhibiting one of the highest H_2_ wt% among characterized borohydrides. The influence of H_2_ pressure (up to 1000 bar H_2_) on the thermodynamics of Mg(BH_4_)_2_ impregnation into carbonaceaous supports was studied recently by White el al. [241]. The field of solid-state electrolytes for batteries was enriched with new examples of Li/Na/Mg nanoconfined borohydrides [66].

Improved hydrogen storage capabilities were recorded through nanoconfinement [54] in C-based materials ([80], high surface area graphite [242], ordered mesoporous carbons like CMK-3 and CMK-8 [241] and mesoporous carbon [243]. Also recorderd were IRH33 carbon [176]) or graphene (G) and its derivatives (G [244], rGO [245] and G-aerogels [241]) as part of RHC (LiBH_4_-Mg(BH_4_)_2_ [148,163,242], the isotopycally-labeled RHC: Li^11^BD_4_-Mg(^11^BD_4_)_2_ [176], or produced in-situ by cycling from the prior-RHC: LiBH_4_-MgH_2_ [180]). In addition, LiBH_4_-NaBH_4_-KBH_4_-Mg(BH_4_)_2_-Ca(BH_4_)_2_ [152,181], hybrid dual-component Mg(BH_4_)_2_-Metal–Organic Borohydride: tetramethylammonium borohydride (TMAB) [246] or ternary systems Mg_2_NiH_4_-LiBH_4_-Mg(BH_4_)_2_ [194]). Catalysts (Al_2_O_3_ produced by atomic layer deposition in γ-Mg(BH_4_)_2_@Al_2_O_3_ composite, Ni-Pt core-shell NPs [243]), additives (MgCl_2_ [247], or others [248], Ti_3_C_2_ MXenes—a 2D layered material [249]) or exhibiting the influence of the nanoscale modification of dehydrogenation product—MgB_2_—on the overall improved hydrogenation behavior [250,251]. Given the high affinity towards catalysts, addition of Ni NPs dispersed in mesoporous carbon yielded clear improvements in hydrogenation behavior of Mg(BH_4_)_2_ [252,253]. Bipyridine-functionalized MOF was shown to improve reversibility of Mg(BH_4_)_2_, potentially through the B-N synergy [254].

#### 2.3.2. Mg(B_3_H_8_)_2_

While probably not enough explored, the speciation of borohydrides is rich and other complex hydrides could be nerated as a result of this. For instance, Mg(B_3_H_8_)_2_ was synthesized and the conversion to tetrahydridoborate [BH_4_]^−^ ion was studied in the RHC: MgH_2_-Mg(B_3_H_8_)_2_ [255]. Mg(B_3_H_8_)_2_ was synthesized by a metathesis reaction between 2NaB_3_H_8_ and MgBr_2_ under Ar in a ball milling process (Reaction (81)).

2NaB_3_H_8_+ MgBr_2_ → Mg(B_3_H_8_)_2_ + 2NaBr (81)

With about 22 wt% conversion [B_3_H_8_]^2−^-to–[BH_4_]^−^, and a decomposition onset as low as ~100 °C, the RHC proposed by Gigante et al. Mg(B_3_H_8_)_2_-4MgH_2_ shows real promise for MgH_2_-Mg(B_3_H_8_)_2_ as a hydrogen carrier, managing tetrahydridoborate conversion (85–88 wt%) below 200 °C within 1 h, without B-losses as toxic boranes (B_2_H_6_ and higher homologues) (Reaction (82)).

Mg(B_3_H_8_)_2_ + 4MgH_2_ → 3Mg(BH_4_)_2_ + 2Mg (82)

In this process, the leading role was that of activated MgH_2_ addition, since neat octahydrotriborate does not undergo this transformation under such mild conditions [255]. Moreover, the use of unsolvated Mg(B_3_H_8_)_2_ affords minimal boranes by-products, in stark contrast to its diglyme adduct that released mainly B_5_H_9_ [255].

#### 2.3.3. Mg(BH_4_)_2_-Adducts/Ammoniates: Case of Mg(BH_4_)_2.6_NH_3_

**Figure 12:**
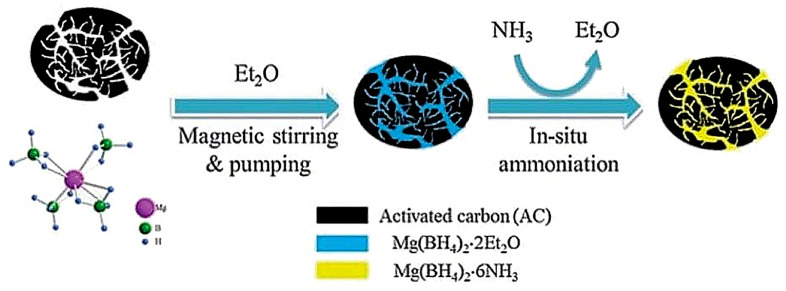
Schematic preparation procedure of the nanoconfined Mg(BH_4_)_2_·6NH_3_. Figure is reprinted with permission from [256].

The practical use of NH_3_-adducts of metal borohydrides is plagued by the exothermic effects. However, upon nanoconfinement into a microporous activated carbon (AC, S_BET_ = 2051 m^2^/g, V_micro_ = 0.835 cm^3^/g) scaffold, the dehydrogenation of the hexaammoniate Mg(BH_4_)_2_·6NH_3_ changes the thermodynamics of the process, which now becomes endothermic with an improvement in H_2_ release temperature of ~40 °C [256]. The microporosity of the AC was found to be a critical factor (mean pore size 4 nm). Notably, the ammoniation was the result of a solvent exchange occurring within AC porosity (Figure 12, Reaction (83)).

(83) $$\mathrm{Mg}({\mathrm{BH}}_{4}{)}_{2}\overset{2{\;\mathrm{Et}}_{2}O;\;\mathrm{AC}}{\to }\;\mathrm{Mg}({\mathrm{BH}}_{4}{)}_{2}\cdots 2{\mathrm{Et}}_{2}O@\mathrm{AC}\overset{6{\;\mathrm{NH}}_{3};-2{\mathrm{Et}}_{2}O}{\to }\;\mathrm{Mg}({\mathrm{BH}}_{4}{)}_{2}\cdots 6{\mathrm{NH}}_{2}@\mathrm{AC}$$

**Figure 13:**
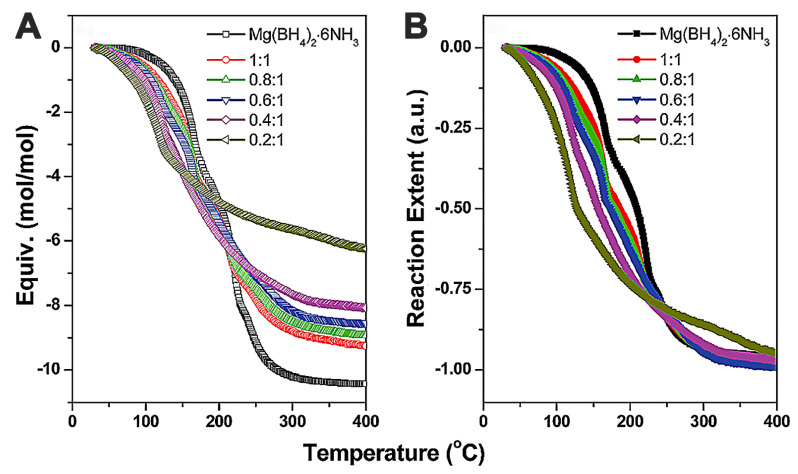
Volumetric release (**A**) and reaction extent (**B**) curves of the bulk Mg(BH_4_)_2_·6NH_3_ and Mg(BH_4_)_2_·6NH_3_@AC nanocomposites. Figure is reprinted with permission from [256].

Among the five investigated samples xMg(BH_4_)_2_·6NH_3_ @ yAC (1:1, 0.8:1, 0.6:1, 0.4:1 and 0.2:1), the 1:1 Mg(BH_4_)_2_·6NH_3_ @ AC sample released H_2_ at a low temperature of ~40 °C, 85 °C lower than its bulk, non-nanoconfined counterpart, with the main dehydrogenation event occurring in the range 150–350 °C *(*Figure 13).

Increasing the AC content lowered the dehydrogenation peak even further. The 0.6Mg(BH_4_)_2_·6NH_3_ @ 1AC had a peak dehydrogenation temperature of 148 °C, with the H_2_ release ending at 240 °C [256]. The intermediacy of a novel Mg-B-N compound was identified; however, rehydrogenation attempts were not successful.

#### 2.3.4. Mg(NH_2_)_2_

Recent reports on magnesium amide are concerned with RHC systems: Mg(NH_2_)_2_-2LiH-0.07KOH [168] and 6Mg(NH_2_)_2_-9LiH-LiBH_4_ co-catalyzed by YCl_3_/Li_3_N [177].

**Figure 14:**
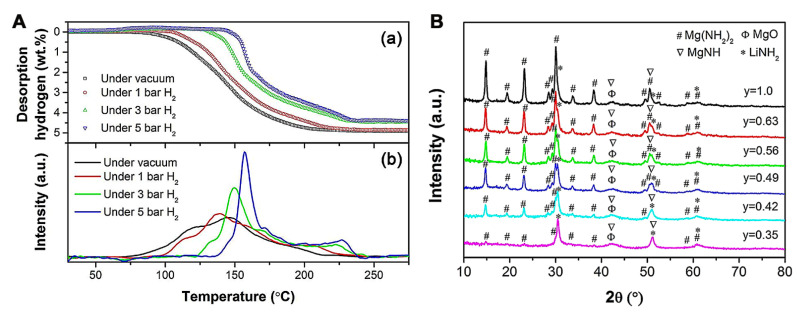
(**A**) Dehydrogenation capacity (**a**) and dehydrogenation velocity (**b**) curves with the temperatures of Mg(NH_2_)_2_-2LiH-0.07KOH under initial vacuum, 1, 3 and 5 bar hydrogen. (**B**) XRD patterns of the yMg(NH_2_)_2_-0.35LiH-0.07KOH samples dehydrogenated to 150 °C under argon carrier gas. Figure is reprinted with permission from ref. [168].

Chen et al. started from an RHC: Li_3_K(NH_2_)_4_–MgNH–LiNH_2_ showed that the dehydrogenation is dependent on p(H_2_). Up to 4.5–4.9 wt% H_2_ was released under pressures up to 5 bar H_2_ (Figure 14).

The role of KOH was important, since it enhances the reversibility of the Mg(NH_2_)_2_–2LiH system (Reaction (59)) [168].

Cao et al. have used chloride (YCl_3_) and nitride (Li_3_N) catalysts to improve the hydrogenation in 6Mg(NH_2_)_2_–9LiH–LiBH_4_ system, and achieved 4.2 wt% H_2_ capacity which can be regenerated applying 85 bar H_2_ (180 °C, 8 min) or 185 bar H_2_ (90 °C) [177]. The nanocrystaline catalysts (2–10 nm) and catalyst-derived species (YH_3_, YB_x_, all nanocrystaline 2–10 nm) provide kinetic alteration to the de/rehydrogenation Reaction (84).

6Mg(NH_2_)_2_ + 9LiH + LiBH_4_ ↔ 3Li_2_Mg_2_(NH)_3_ + Li_4_(BH_4_)(NH_2_)_3_ + 9H_2_ (84)

**Figure 15:**
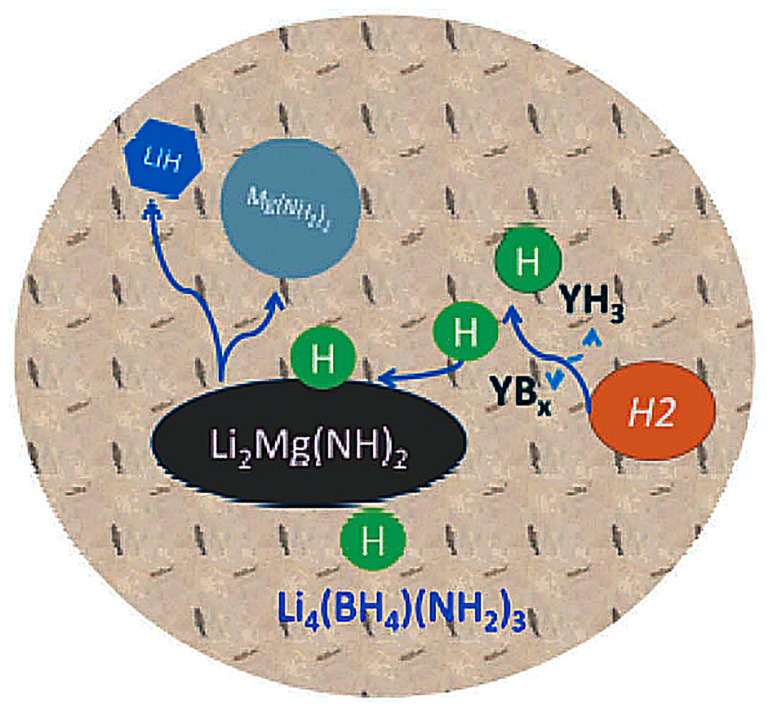
The proposed absorption reaction mechanism of the co-added sample. Figure is reprinted with permission from ref. [177].

**Figure 16:**
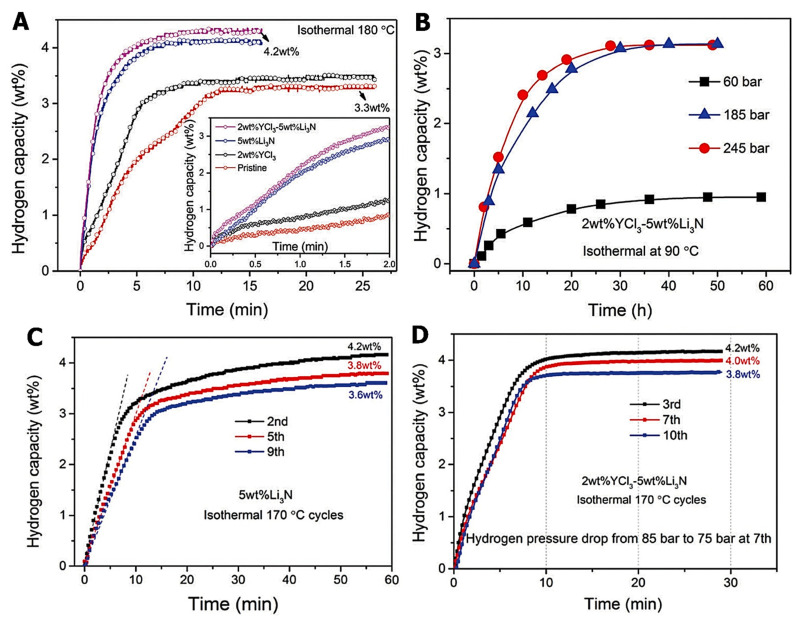
(**A**) Isothermal hydrogenation curves of the pristine system, and 2 wt% YCl_3_, 5 wt% Li_3_N and 2 wt% YCl_3_-5 wt% Li_3_N-co-added samples at 180 °C and 50 bar. Inset plot: isothermal hydrogenation curves of these samples during the first 2 min; (**B**) isothermal hydrogenation of the 2 wt% YCl_3_-5 wt% Li_3_N-co-added sample at 90 °C under 60, 185 and 245 bar of H_2_, respectively. (Before hydrogenation, all the samples were fully dehydrogenated under isothermal conditions at 180 °C). Isothermal hydrogenation curves of (**C**) the second, fifth and ninth cycle of the 5 wt% Li_3_N-added sample, and (**D**) the third, seventh and tenth cycle of the co-added sample, at 170 °C and under 85 bar of H_2_. The H_2_ pressure during the absorption measurement decreased from 85 to 75 bar in the 7th cycle. This might be one of the reasons for the reduced reversible hydrogen capacity. This figure was reprinted with permission from ref. [177].

The origin of the Li_4_(BH_4_)(NH_2_)_3_ product can be traced back to the successive reactions occurring within the proposed RHC system (Reactions (85)–(87)) (Figure 15 and Figure 16) [177].

3LiH + YCl_3_ → YH_3_ + 3LiCl (85)

Li_3_N + 2H_2_ → LiNH_2_ + 2LiH (86)

3LiNH_2_ + LiBH_4_ → Li_4_(BH_4_)(NH_2_)_3_ (87)

#### 2.3.5. Mg(AlH_4_)_2_

**Figure 17:**
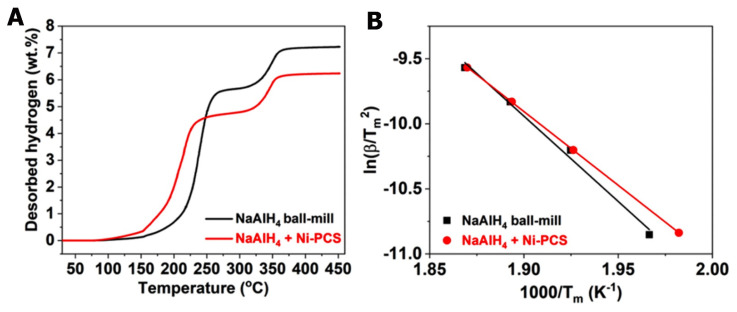
(**A**) Hydrogen desorption capacity curves of ball milled Mg(AlH_4_)_2_ and Mg(AlH_4_)_2_ with Ni-PCS. (**B**) Kissinger’s plots for dehydrogenation of ball milled Mg(AlH_4_)_2_ (box solid, Ea = 118 kJ·mol^−1^) and Mg(AlH_4_)_2_ with Ni-PCS (red-colored circle solid, Ea = 103 kJ·mol^−1^). Figure was reprinted with permission from ref. [125].

Only a few reports of Mg(AlH_4_)_2_ have been published so far [125,257]. When confined into nickel-containing porous carbon sheets (Ni-PCSs, S_BET_ = 2572 m^2^/g, pore size 2.8 nm), LiAlH_4_, NaAlH_4_ and Mg(AlH_4_)_2_ were shown to lower the desorption temperature by as much as 29 °C (Figure 17) [125].

The Mg(AlH_4_)_2_-Ni-PCS sample started to desorb H_2_ at 125 °C (146 °C in ball-milled version), with a total hydrogen content of 3.34 wt%*—*a lower amount compared to the theoretical 9.3 wt% due to the inclusion in a porous support, but also due to added weight of LiCl from metathetical synthesis of Mg(AlH_4_)_2_ (LiAlH_4_ and MgCl_2_) [125].

Xiao et al. synthesized Mg(AlH_4_)_2_ nanoparticles (2–7 nm) using a solvent-free, mechanochemical strategy and studied their hydrogenation properties [257]. These NPs start to desorb H_2_ at 80 °C (completing the first dehydrogenation step at 120 °C, 30 min), recording a 65 °C improvement compared to microparticles of Mg(AlH_4_)_2_ [257]. Similar improvement was deduced regarding the activation energy of dehydrogenation: a reduced value of 105.3 kJ/mol vs. 123.6 kJ/mol in micro-Mg(AlH_4_)_2_. Interestingly, the presence of LiCl from the metathesis reaction was found to keep the Mg/MgH_2_ products in the nanorange (<10 nm), hence improving the cycling behavior of the system [257].

### 2.4. Other First Group-Derived Borohydrides

Since the general trend regarding stability of alkali metal borohydrides is to increase down the group 1 elements of the periodic table, the thermodynamic destabilization should be even greater in order to bring their operation regime into a fuel cell achievable domain (100–200 °C). Hence, the reports on heavier alkali metal borohydrides, coupled with their lower theoretical H_2_ content, are rather scarce. For instance, only a few reports of RbBH_4_ and CsBH_4_ exist*—*and which concern reorientation mobility of [BH_4_]^−^ anions [71], a report on complex hydride K[Al(NH_2_BH_3_)_4_] [258] and slightly more research performed on lighter KBH_4_ [60,71,152,160,170,181,194]. In one of these reports, a mixture of complex hydrides (LiBH_4_/KBH_4_) was used as an electrolyte to perform full rehydrogenation in the MgH_2_/Sn coupled system [160].

Bearing the familiar amidoborane ligand, K[Al(NH_2_BH_3_)_4_] was synthesized by Møller et al. and featured a triclinic unit cell crystal structure (P-1) [258]. Following a mechanochemical approach, KAlH_4_ and AB (NH_3_BH_3_) were milled and afforded the complex hydride K[Al(NH_2_BH_3_)_4_] which featured two-step, exothermic decomposition peaks in DSC analysis (94 °C and 138 °C), releasing a total of ~6.0 wt% H_2_ along with amorphous KBH_4_ (^11^B-and ^27^Al NMR) [258]. Still, the system was not reversible under employed conditions (260 °C, 110 bar H_2_).

Nanostructuring KBH_4_-LiBH_4_ RHC into CMK-3 type ordered mesoporous carbon has shown improved dehydrogenation behavior [170]. Exploiting an eutectic composition of 0.725 LiBH_4_–0.275 KBH_4_ featuring a low melting point (105 °C), melt-infiltration into mesoporous carbon CMK-3 afforded a reversible H_2_ uptake-release of 2.5*–*3.0 wt% during five hydrogenation cycles [170]. The quinary equimolar mixture LiBH_4_-NaBH_4_-KBH_4_-Mg(BH_4_)_2_-Ca(BH_4_)_2_ was investigated by Dematteis et al. [181], and showed that the pure borohydrides only form KCa(BH_4_)_3_ as a new phase, yielding a liquid phase (after up to 50 h of ball milling) from which dehydrogenation was shown to be a complicated, multi-step process. This approach of combining five borohydrides relied on the concept of high energy alloys [181]. Dematteis et al. have also explored the opportunity of Mg_2_NiH_4_*—*confirmed to act as a hydrogen pump in the Ni-doped Mg/MgH_2_ system*—*to form novel RHC-like systems Mg_2_NiH_4_-LiBH_4_-M(BH_4_)_x_ (M = Na, K, Mg, Ca) [194]. In particular, the Mg_2_NiH_4_-LiBH_4_-KBH_4_ was prepared by ball milling in the eutectic borohydride composition (reversible, mp = 110 °C), yielding 0.56 Mg_2_NiH_4_, 0.32 LiBH_4_, 0.12 KBH_4_ [194]. Even so, a modest temperature enhancement relative to the bulk was recorded (ΔT = −10 °C) and the PXD spectra confirmed formation of Mg_2_NiH_0.3_, MgH_2_ and MgNi_2.5_B_2_ only from reaction of LiBH_4_ with Mg_2_NiH_4_, with KBH_4_ remaining largely unaffected [194].

### 2.5. Ca-Based Complex Hydrides; Ca(BH_4_)_2_

Magnesium and calcium are two representative examples of group 2 complex hydrides. Ca(BH_4_)_2_ in particular has some attractive features that set it apart from its Mg-counterpart.

Ca(BH_4_)_2_ was also investigated in the context of RHC when mixed with other borohydrides in the ternary/quaternary [152] or quinary [181] systems based on mixtures LiBH_4_-NaBH_4_-KBH_4_-Mg(BH_4_)_2_-Ca(BH_4_)_2_, while DFT computations shed light on the decomposition of Ca(BH_4_)_2_ altered by nanoconfinement effects [259]. In the quantum mechanical computations, thin films of β-Ca(BH_4_)_2_ were shown to afford the decrease of dehydrogenation enthalpy by ΔH = −5 kJ/mol H_2_, through the formation of the active intermediates CaH_2_ (path (88a)) or CaB_2_ (pathway (88b), Reaction (88)) [259].

(88) $${\mathrm{CaH}}_{2}+2B+3H_{2}\;\overset{a}{\leftarrow }\;\mathrm{Ca}({\mathrm{BH}}_{4}{)}_{2}\overset{b}{\to }2/3{\mathrm{CaH}}_{2}+1/3{\mathrm{CaB}}_{6}+10/3H_{2}$$

**Figure 18:**
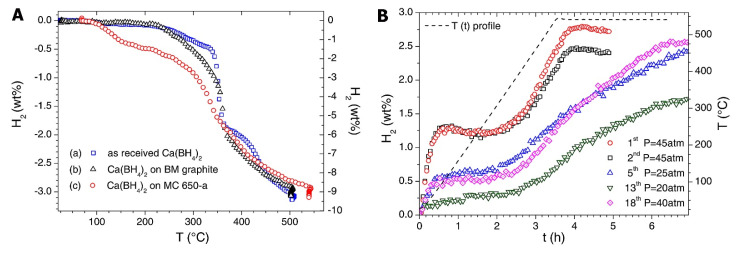
(**A**) TPD curves of Ca(BH_4_)_2_ with a heating rate of 2.5 °C min^−1^. Use vertical scale on the right for (a) as-received material; use scale on the left for Ca(BH_4_)_2_ on ball-milled graphite (b) and on MC 650-a (c). (**B**) TPA curves of nanocomposite Ca(BH_4_)_2_@MC 650-a for successive hydrogenation cycles at different pressures. Reprinted with permission from Ref. [260].

Nanoconfinement effects in actual physical systems were confirmed by Comanescu et al., who designed a micro-mesoporous carbon scaffold and used the activated scaffold (MC 650-a, S_BET_ = 1780 m^2^/g, V_pore_ = 1 cm^3^/g) of high porosity to confine Ca(BH_4_)_2_ by the incipient wetness method of an MTBE solution of calcium borohydride. This approach led to ~ 2.4 wt% reversible and roughly stable H_2_ capacity over 18 cycles of hydrogen release/uptake (Figure 18*)*. Remarkably, the onset of H_2_ desorption was reduced to ~100 °C and rehydrogenation conditions were significantly improved, compared to bulk Ca(BH_4_)_2_ (20–45 atm H_2_, 6.5 h). Additionally, ~68.7% of Ca(BH_4_)_2_ was proved to behave reversibly after the evaluation of support weight contribution to the Ca(BH_4_)_2_@MC-a nanocomposite [260].

Other supports for Ca(BH_4_)_2_ confinement were also investigated: SBA-15 and MCM-41 (RHC: LiBH_4_-Ca(BH_4_)_2_) [158], mesoporous carbon (RHC, eutectic composition LiBH_4_-Ca(BH_4_)_2_) [162], activated carbon aerogel (RHC: LiBH_4_−Ca(BH_4_)_2_) [175], ordered mesoporous carbon CMK-3 (1320 m^2^/g, 1.48 cm^3^/g) catalyzed by TiCl_3_ additive [261]. Other RHCs containing Mg_2_NiH_4_ (Mg_2_NiH_4_-LiBH_4_-Ca(BH_4_)_x_) highlighted the role of complex hydride Mg_2_NiH_4_ in lowering the desorption temperature of the eutectic mixture of borohydrides [194]. Even in the absence of a Ni-based hydride, reversibility of LiBH_4_ was enhanced by using the eutectic composition of RHC: LiBH_4_-Ca(BH_4_)_2_, namely 0.68LiBH_4_–0.32Ca(BH_4_)_2_, when nanoconfined into high porosity carbon aerogel (S_BET_ = 2421 ± 189 m^2^/g, V_tot_ = 2.46 ± 0.46 mL/g, 13 nm pore size) [182]. The catalyst effect strategy was explored in the system comprising 2NaAlH_4_ and Ca(BH_4_)_2_, when enhancements were recorded upon TiF_3_ addition, presumably due to the formation of intermediates of the form [AlF_6_]^3−^ [183]. General catalytic principles and implications of using d-block metal derivatives were reviewed in correlation to metal hydride gravimetric capacity, and their potential for kinetic improvements. Among them, Ni, Co, V, Ti, Fe and Nb appear to have offered the most promising results [262].

### 2.6. Al-Based Complex Hydrides

While Al(BH_4_)_3_ may have a very high theoretical storage capacity, its volatile and extremely reactive nature have precluded its use as a hydrogen storage material. However, the corresponding ammoniate Al(BH_4_)_3_(NH_3_)_6_ could be stabilized by confinement in a PSDB (poly(styrene-co-divinylbenzene) polymeric matrix [263] where it also showed partially-reversible behavior using a hydrazine/ammonia treatment step. An interesting detail regarding the report from Tang et al. is the understanding that the diffusion of Al(BH_4_)_3_ (produced by mixing AlCl_3_ and LiBH_4_ in a 1:3 stoichiometric ratio) into the pores of PSDB was enhanced by complexation to the phenyl rings of PSDB to produce Al(BH_4_)_3_/PSDB nanocomposites. Subsequent exposure to ammonia afforded final Al(BH_4_)_3_·6NH_3_/PSDB nanocomposites. The host could also be tuned for inorganic component, such as nanostructured porous carbon [264]. Inclusion of Al(BH_4_)_3_(NH_3_)_6_ in pore-expanded mesoporous carbon with high textural characteristics (S_BET_ = 980 m^2^/g, V_pore_ = 1.629 cm^3^/g and d_pore_ = 12.7 nm) had a beneficial effect in reducing borane emissions from the composite ammoniate@nanoporous carbon, yielding an H_2_ purity of up to 93.5%.

### 2.7. TM- and RE-Based Complex Hydrides

#### 2.7.1. Adducts/Ammoniates: Zr(BH_4_)_4.8_NH_3_

Featuring a low desorption temperature, Zr(BH_4_)_4_.8NH_3_ is considered a promising material for hydrogen storage and was recently synthesized by Wu et al. [265] using a physical vapor deposition approach, terming the method “heating-ball milling vial”. Additionally, 10 wt% NaBH_4_ inclusion in Zr(BH_4_)_4_.8NH_3_-10 wt%NaBH_4_ RHC showed a thermodynamioc improvement by lowering the desorption temperature from 130 °C (bulk ammoniate) to 75 °C (composite), while also supressing alternative reaction pathways leading to B_2_H_6_/NH_3_ (Reaction (89)) [265].

#### 2.7.2. NaMgH_3_ and NaZn(BH_4_)_3_

The NaMgH_3_ perovskite type complex ternary hydride synthesized from MgH_2_ and NaH, and activated by a ball milling approach (under Ar, 2–15 h) showed a two-step decomposition behavior, releasing 5.8 wt% H_2_ from 287 °C to 408 °C within 2 h [266]. Notably, rehydrogenation of decomposed products were rehydrogenated under 10 bar H_2_, at ~200 °C [266].

NaMgH_3_ → NaH + Mg + H_2_ → Na + Mg + H_2_ (89)

The Rietveld refinement of rehydrogenated samples XRD data showed a regeneration of NaMgH_3_ (27 wt%), NaH (6 wt%) and oxides (67 wt%) [266].

**Figure 19:**
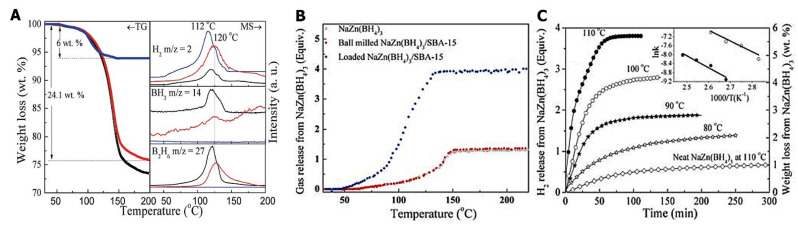
(**A**) TGA and MS results for pure NaZn(BH4)3 (black line), ball milled NaZn(BH_4_)_3_/SBA-15 (red line) and loaded NaZn(BH_4_)_3_/SBA-15 (blue line),with a heating rate of 2 C/min under dynamic Ar atmosphere. The right axis of the TGA chart gives the amount of weight loss relative to the mass of NaZn(BH_4_)_3_ only. (**B**) Volumetric gas release measurements for pure NaZn(BH_4_)_3_(black line), ball milled NaZn(BH_4_)_3_/SBA-15 (red line) and loaded NaZn(BH_4_)_3_/SBA-15 (blue line), with a heating rate of 2 °C/min under 1 atm argon, expressed with respect to the content of NaZn(BH_4_)_3_ only. (**C**) Isothermal volumetric results for gas release from the loaded NaZn(BH_4_)_3_/SBA-15 composite at 80 °C, 90 °C,100 °C and 110 °C, and for pure NaZn(BH_4_)_3_ at 110 °C. The inset shows a comparison of the Arrhenius plots of the temperature-dependent rate data of the loaded NaZn(BH_4_)_3_/SBA-15 composite (full circles) and the pure NaZn(BH_4_)_3_ (empty circles). This figure was reprinted with permission from ref. [267].

Nanoconfinement strategy was applied in the case of NaZn(BH_4_)_3_ by the inclusion of a complex hydride into the mesoporosity of SBA-15 silica*—*S_BET_ = 274.6 m^2^/g; V_pore_ = 0.4596 cm^3^/g*—*(hydride:support weight ratio 1:3, ball milling, 1 h, and infiltration of 25 drops NaZn(BH_4_)_3_–THF solution over 0.2 g SBA-15). Pure hydrogen was released in the range 50–150 °C, and a reduction of 5.3 kJ/mol in the activation energy was computed based on the Arrhenius plot (E_A_ = 38.9 kJ/mol for nanoconfined NaZn(BH_4_)_3_@SBA-15) (Figure 19) [267].

The decomposition reaction can be formulated according to Reaction (90), and is supported by observance of NaBH_4_, amorphous B and Zn among decomposition products, along with the release of pure H_2_ (6 wt%); however, rehydrogenation attempts in the solid state (400 °C, 10 MPa H*_2_*) were unsuccessful [267].

NaZn(BH_4_)_3_ (nano) → NaBH_4_ + Zn + B + 2H_2_ (90)

#### 2.7.3. XTiH_3_ (CaTiH_3_, MgTiH_3_)

CaTiH_3_ and MgTiH_3_ perovskite hydrides were studied by Selgin using DFT and showed the predicted 4.01 wt% hydrogen storage for MgTiH_3_ based on electronic band structures and energy of states computation (ductile material) vs. CaTiH_3_ (brittle nature) [268].

#### 2.7.4. Mg_2_NiH_4_

While not yet a commercial material, Mg_2_NiH_4_ can be synthesized from MgH_2_ and Ni, and was utilized in a series of RHC: in LiBH_4_–Mg_2_NiH_4_ where it showed mutually-destabilized system [153]; in the formation of nanocomposites Mg_2_NiH_4_@G nanosheets, where the surface MgO layer actually protected the complex hydride from further oxidation; in affording a low E_A_ = 31.2 kJ/mol [269] and in LiAlH_4_-Mg_2_NiH_4_ doped with TiF_3_ affording a dehydrogenation onset of 50 °C and E_A_ = 81.56 kJ/mol [161]. In addition, in the formation in-situ of Mg_2_NiH_4_-Mg_2_Ni@MOF from a MgH_2_@Ni-MOF starting nanocomposite (ΔH_des_ = 69.7 ± 2.7 kJ/mol; E_A,des_ = 144.7 ± 7.8 kJ/mol H_2_; ΔH_abs_ = −65.7 ± 2.1 kJ/mol; E_A,abs_ = 41.5 ± 3.7 kJ/mol) [270], or in Mg_2_NiH_4_-LiBH_4_-M(BH_4_)_x_ (M = Na, K, Mg, Ca) composites where it afforded improvement in thermodynamic behavior by reduction with up to 40 °C the desorption temperature [194].

#### 2.7.5. Mg_2_FeH_6_

The ternary hydride Mg_2_FeH_6_ was synthesized (2.1 MgH_2_: 1 Fe, ball milling for 15 h, 200 rpm, 20 bar H_2_) and used in composite systems Mg_2_FeH_6_ + 4LiNH_2_ (Reaction (36)), allowing further reactivity enhancement that eliminated ~4.8 wt% H_2_ at 225 °C and absorbed 3.7 wt% H_2_ at 200 °C and 50 bar H_2_ [137]. The report from Zhang et al. also highlights that the ternary hydride Li_4_FeH_6_ could be synthesized for the first time in a metathetical reaction, rather than applying GPa H_2_ pressure, as previously reported in the literature [137].

When used in a (1 − x)LiBH_4_ + xMg_2_FeH_6_ nanocomposite, the system showed for composition x = 0.5 a reversible behavior at 400 °C and 20 MPa H_2_ for more than four cycles [186].

#### 2.7.6. K_2_Mn(NH_2_)_4_

K_2_Mn(NH_2_)_4_ was synthesized by ball milling Mn and K under seven bar NH_3_, and was utilized in the RHC K_2_Mn(NH_2_)_4_–8LiH where it released 6.0 wt% H_2_ in an open system. Moreover, very fast reabsorption kinetics (>1 wt%/min) allowed RHC recharging within minutes, at 230 °C and 50 bar H_2_ [173]. The final identifiable products for dehydrogenation were Li_2_NH, Mn_3_N_2_ and MnN, while after rehydrogenation an unknown “K-Mn-species2” was produced, alongside LiH and LiNH_2_ [173].

#### 2.7.7. Ti(BH_4_)_4_

Ti(BH_4_)_3_ could be a promising candidate for H_2_ storage application (13.0 wt% theoretical storage capacity), if it were not for its high instability*—*it spontaneously decomposes at RT. Although volatile in pristine form, Ti(BH_4_)_3_ was synthesized and stabilized by the nanoconfinement strategy (trapping at 200 K) in the MOF structure of UiO-66 ((Zr_6_O_4_(BDC)_6_, BDC = 1,4-benzenedicarboxylate, S_BET_ = 1200 m^2^/g). LiBH_4_ and TiCl_3_ were milled in a 3:1 molar ratio using a planetary mill (10 min) (Reaction (91)).

3LiBH_4_ + TiCl_3_ → Ti(BH_4_)_3_ + 3LiCl (91)

While it would immediately decompose in bulk form into B_2_H_6_ and TiH_2_, the nanoconfined composite Ti(BH_4_)_3_@MOF (S_BET_ = 770 m^2^/g, confirming confinement of borohydride species, but also that the pores have not been completely filled) was stable up to 350 K under vacuum, which is a great improvement in stabilization of such a reactive species [271]. The narrow cage size of UiO-66 (1.6–1.7 nm diameter) was demonstrably essential in achieving this stabilization effect.

#### 2.7.8. (RE)(BH_4_)_x_

Frommen et al. studied the thermal decomposition of rare earth (RE) borohydrides*—*either obtainable starting from RECl_3_-LiBH_4_ mixtures (molar ratios 1:3 and 1:4), yielding RE(BH_4_)_3_/LiRE(BH_4_)_3_Cl and LiRE(BH_4_)_4_, respectively, (Reactions (92) and (93)), but also by exploring wet chemical sysnthesis which allow for a LiCl-free borohydride upon filtering and subsequent vacuum drying [272].

LiRE(BH_4_)3Cl + 2LiCl ← RECl_3_ + 3LiBH_4_ → RE(BH_4_)_3_ + 3LiCl (92)

RECl3 + 4LiBH_4_ → LiRE(BH_4_)_4_ + 3LiCl (93)

Y(BH_4_)_3_ is probably among the most promising RE borohydride due to its high gravimetric hydrogen capacity (9.1 wt%), but also due to reasonable dehydrogenation temperature (onset at 187 °C, peak at 250 °C).

Heere et al. investigated the composite 3LiBH_4_ + Er(BH_4_)_3_ + 3LiH (9 wt% theoretical hydrogen capacity) and revealed a practical hydrogen release of 4.2, 3.7 and 3.5 wt% during the first three a/d cycles [171].

### 2.8. Ammonia Borane (AB) and Related Compounds

Ammonia-borane remains a highly investigated material, due essentially to its high hydrogen capacity. However, the system is not reversible without nanoconfinement into mesoporous materials [273] or other further tuning [274,275,276,277,278,279]. Yet, mechanistic investigations have not completely elucidated the dehydrogenation pathways and as such, rehydrogenation mechanisms are still under investigation to date.

#### 2.8.1. NH_3_BH_3_ (AB)

In general, nanoconfinement of AB (NH_3_BH_3_, 19.6 wt% theoretical gravimetric capacity) into mesoporosity of various supports was shown to promote H_2_ desorption starting at a lower temperature onset. Various supports were investigated, including mesoporous silica, GaO_3_ and Co-substituted AlPO_4_-5 (when polyiminoborane was not detected among thermolysis of AB@support using operando Raman-Mass Spectrometry, suggesting AB-SBA-15 interaction modifying typical decomposition pathway) [273]. Nanosized (average size 110 nm) AB synthesis using cetyltrimethylammonium bromide (CTAB) was also investigated, in addition to dodecane C_12_H_26_ as a counter-solvent in aqueous media (overall weight loss between 80–200 °C of 24–57.3 wt%) [280]. Mechanistic investigations of energy release from AB included using different oxidants (NH_4_ClO_4_ rerouting the decomposition pathway of AB through [NH_3_BH_2_NH_3_]^+^[ClO_4_]^−^ salt which inhibits BNH_x_ species and affords AB complete oxidation) [281], the effect of polyacrylamide-grafted organically modified mesoporous silica (PAM-COOH-MSNs and PAM-Ph-MSNs) as nanocarriers for AB confinement [282].Further investigations include silica aerogel scaffold (up to 60 wt% AB loading, thermolysis onset at 80 °C due to SiOH and SiOSi groups involed in the interaction AB-SiO_2_ silica) [283], ZIF-67-derived fcc-Co@porous carbon nano/microparticles used as catalysts to enhance H_2_ release from AB [284], carbon nanotubes CNTs array (CMK-5 with 1650 m^2^ g^−1^ and 1.69 cm^3^ g^−1^, bimodal porosity; 9.4 wt% H_2_ released from (30AB:30AlH_3_)@CMK-5 nanocomposite at 95 °C in 10 min) [285]. Moreover, investigation subjects include nanoporous carbon (termed MDC –S_BET_ = 2222 m^2^/g, 2.49 cm^3^/g, 0.40 nm pore size–, obtained from MOF-5 calcination in N_2_ at 1000 °C, yielding after confinement AB@MDC composites using a solution infiltration technique; 4.7 wt% H_2_ was released at 80 °C with the lowest t_onset_ = 72 °C) [286]. GO/rGO derivatives (AB@GO and AB@rGO integrated AB into graphene derivative without solvent or melt infiltration, in a one-step “ice-templating” process, releasing no harmful gases such as B_2_H_6_, B_3_H_6_N_3_ or NH_3_) [287]. TiO_2_(B)-catalyzed C-scaffold (synergistic role of C-TiO_2_(B) in the enhancement of H_2_ desorption from C-TiO_2_(B)/NH_3_BH_3_ nanocomposites probably by added H+ ions from TiO_2_ hydrolysis reaction) [288]. Microporous carbon with narrow PSD of 1.05 nm pore size (t_onset_ = 50 °C, main event at 86 °C and ~12 wt% H_2_ release at 90 °C in 30 min) [289]. The study of MOF nature and effect on AB dehydrogenation behavior (MOFs: IRMOF-1, IRMOF-10, UiO-66, UiO-67 and MIL-53(Al)) [290], of the effect of nanoconfinement into MOF with MIL-53 topology: Al-MIL-53 (86% H_2_ release), Al-MIL-OH (38% H_2_ release) and Al-MIL-NH_2_ (67 wt% H_2_ release within 60–110 °C, suggesting interaction AB–functional groups on MOFs) [291]. Mesoporous monolithic BN as AB–scaffolds (S_BET_ = 584–728 m^2^ g^−1^, high V_pore_ = 0.75–0.93 cm^3^/g. H_2_ release at 100 °C up to 8.1 wt%) [292]. Ni-matrix affording ~50 nm size reduction of AB (suppression of toxic gases like diborane, and rehydrogenation occurring at 200 °C under 6 MPa H_2_) [293]. Investigation of an AB/PEO (polyethylene oxide) cocrystal for the potential energy storage applications [294]. Incorporation of AB into Pd/halloysite nanotubes (small Pd catalyst sizes of ~1.4 nm; H_2_ release was recorded at 60 °C and E_A_ = 46 kJ/mol vs. 183 kJ/mol for neat AB) [295]. Evaluation of the feasible usage of 40:60 wt% AB:AC nanococomposite in a portable power tank (solution impregnation; ~6.0 wt% H_2_ with t_onset_ = 96 °C) [279]. Alternatively, using less explored hosts such as hypercrosslinked porous poly(styrene-co-divinylbenzene) resin (PSDB) for AB nanoconfinement [296]. Shen et al. have used Cu NPs and showed that 6.8 nm Cu NPs are active catalysts in the decomposition of NH_3_BH_3_ to release H_2_, as well as affording pure polybenzoxazole (PBO) in a one-pot reaction of NH_3_BH_3_, diisopropoxy-dinitrobenzene and terephthalaldehyde. [297]. Highly pure and chemically-resistant PBO (M_W_ = 19 kDa) was also reported by the same group using 8–18 nm Cu_2_O, as a catalyst also able to enhance ammonia borane dehydrogenation [298].

#### 2.8.2. Tetraalkyl Ammonium Borohydrides [NR_4_][BH_4_]

The parent ammonium borohydride, NH_4_BH_4_ (ABH_2_), has a high gravimetric capacity of 18 wt% H_2_ that can be released below 160 °C. However, decomposition occurs even at room temperature into [(NH_3_)_2_BH_2_][BH_4_] (DADB) and hence, a stabilization method needs to be developed before adopting its wider use as a H_2_ storing media. To this end, MCM-41 mesoporous silica was used for nanoconfinement of ABH_2_, allowing its storage at temperatures below −30 °C [299].

Tetraalkyl ammonium borohydrides [NR_4_][BH_4_] are a novel class of organic borohydrides that were investigated for CO_2_ capture via triformatoborohydride ([HB(OCHO)_3_]^−^) and converted to more useful chemicals [300].

## 3. Improvement Strategies and Most Encouraging Results—An Overview

Porous materials have emerged as a class of materials featuring high versatility regarding both structure and envisioned applications. They can be tailored to accommodate smaller or larger reagents, offering shorter or longer reaction times depending on the application field [113,208,240]. In the recent past, these materials have been explored in the energy storage field: MOFs [204,212,270,301,302,303], carbon nanomaterials [100,101,108,126,127,172,175,176,178,210,227,228,242,286,288,304,305], graphene derivatives [269,287], MXenes [159,184,225,306,307], TM oxides (CeO_2_ [223]), siloxanic materials [93,107,189,267], ordered mesoporous carbon OMC [102,106,170,179,185,191,209,224,261], metal scaffolds for microencapsulation (Al [234]), polymers [296] and nitrides [105]. Machine learning was recently employed for predicting the hydrogen release of systems based on lithium borohydride [58,62].

**Figure 20:**
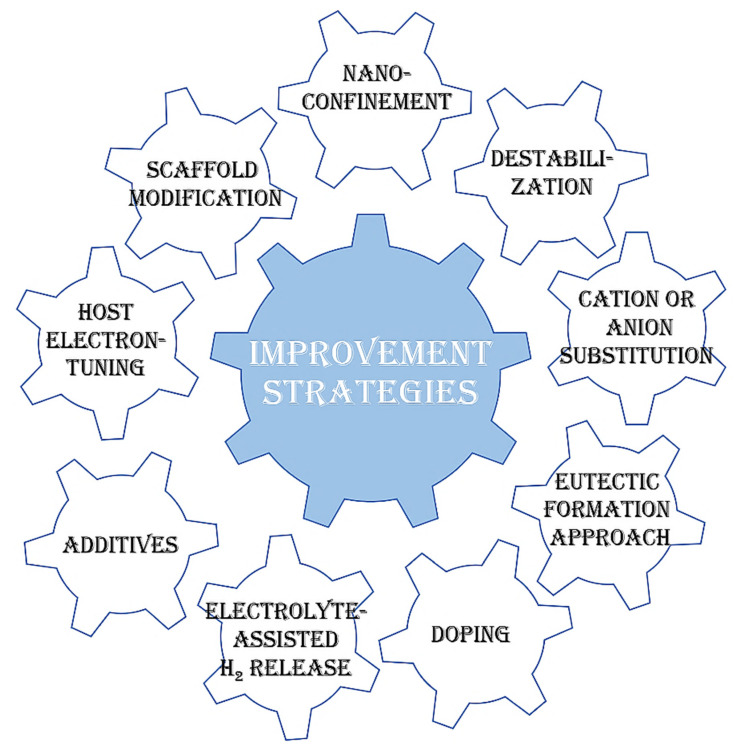
Improvement avenues that were explored to enhance hydrogenation effectiveness in hydride-based systems.

Improvement attempts have tackled the hydrogen storage issue from various angles, and today many approaches have shown their effectiveness (Figure 20). The current strategies that were summarized in Figure 20 are detailed as follows.

### 3.1. Nanoconfinement

Nanoconfinement was a preferred strategy to improve hydrogenation behavior by incorporating simple or complex hydrides in MOFs [204,212,270,290,291,301,302,303,306]; metal-doped carbon originating from thermally-collapsed MOFs (Co-[284]) and(Zn-[286]); carbon materials [232,305]; microporous carbon [256]; mesoporous carbon [72,162,170,179,185,210,253,261]; micro-mesoporous carbon (0.5–4.5 nm, IRH33 [176]); nanoporous carbon aerogels [163,169,227]; CO_2_–activated carbon aerogel [174,175]; TiCl_3_-catalyzed carbon aerogels [178,228]; ScOCl-catalyzed carbon aerogel [211]; carbon nanofibers CNFs [95,172]; carbon nanotubes CNTs [101,285]; activated carbon catalyzed by metal salts (CeF_3_ [100]); graphene G; graphene oxide GO and reduced graphene oxide rGO (GO, rGO–[287]), Fe_3_O_4_–G [98] and G-MC [99]; highly microporous carbon (PSD~ 1 nm, [289]); ordered mesoporous carbon OMC [106,191,209,224]; SWCNTs [108]; Zr-CMK-3 [308]; MWCNTs [164,229]; high surface area graphite [242]; fluorographite [130]; hollow carbon nanospheres HCNs [127]; various other mesoporous materials (BN–[292]), Ni-matrix [293] and Pd/halloysite NTs [295]); 1D nanoporous materials [304]; CeO_2_ hollow nanotubes HNTs [223]; silica-based scaffolds [74,75,76,93,107,158,189,267]; MXenes Ti_3_C_2_ [96,159,184,307]; CeF_3_-Ti_3_C_2_ MXene [225]; C@TiO_2_ 2D scaffold [226]; nanoporous Ni-based alloy [97]; Ni–Pt core-shell nanoparticles [243]; Ni-catalyzed C nanosheets [125]; TiO_2_/porous C [126]; NbF_5_–MC [102]; porous Al scaffold [234]; h-BN [131]; polymeric matrix (hypercrosslinked porous poly(styrene-co-divinylbenzene) resin [296]) or PcB (poly (methyl methacrylate)–co–butyl methacrylate) [229].

### 3.2. Destabilization

Two apparently divergent strategies were pursued. Firstly, the stabilization of complex hydride systems that are too unstable for practical use under normal conditions and above (like Al(BH_4_)_3_ in the form of ammoniate complex Al(BH_4_)_3_.6NH_3_, or Ti(BH_4_)_3_ for instance). Secondly, the destabilization of those compounds that present too high thermodynamic stability such that they lower their desorption/resorption into the achievable realm (100–150 °C ideally). However, due to the high thermal stability of complex hydrides in general, the destabilization methods prevail [309]. More specifically, nanostructured γ-Mg(BH_4_)_2_ was destabilized by the Al_2_O_3_ atomic layer [310], mutual destabilization is typically observed in RHC comprising metal borohydrides (LiBH_4_–NaBH_4_ [155]), RHCs based on borohydride-light hydride (LiBH_4_-AlH_3_ [167]), borohydride-alanate systems (NaBH_4_-Li_3_AlH_6_ [132]), SiS_2_-destabilized light borohydrides (6LiBH_4_–SiS_2_, 8.2 wt%H_2_ released with t_onset_ = 92 °C [103]), TM fluorides destabilize alkali borohydrides (NaBH_4_ [196,230]) and ionic liquids (ILs) were also reported to destabilize NaBH_4_ (vinylbenzyl trimethylammonium chloride IL [231]). Others have reported confinement strategies to stabilize/destabilize NH_4_BH_4_ [299], or the synergistic effect of ternary/binary Mg-hydrides (Mg(AlH_4_)_2_/MgH_2_ [257]).

### 3.3. Cation/Anion Substitution

Utilizing a catalyzed RHC system is a proven strategy to improve hydrogenation behavior. For instance, the nanoconfined 2LiBH_4_–MgH_2_–0.13TiCl_4_ system showed good reversibility behavior possibly due to the formation of Ti-MgH_2_ alloys (Mg_0.25_Ti_0.75_H_2_ and Mg_6_TiH_2_, which can be regarded as cation substitutions of Mg^2+^ in MgH_2_ by Ti^3+^ [154]). By forming eutectic compositions, mixed alkali borohydrides could be formulated (Li_0.65_Na_0.35_BH_4_– Li_0.70_Na_0.30_BH_4_ [155]) as well as anion substitutions (I^−^/NH_2_^−^ substitutions of BH_4_^−^, in LiBH_4_–LiI and LiBH_4_-LiNH_2_ composites producing Li_2_(BH_4_)_x_I_1−x_ and Li_2_(BH_4_)_x_(NH_2_)_1−x_ [139]). The presence of F^−^ anion in the 3NaBH_4_-GdF_3_ system produced dehydrogenation in the LT stage of NaBH_4_, due to F^−^ substitution of H^−^ [196]. The stability of mixed-cation, mixed-anion borohydrides was also evaluated for solid electrolyte application in batteries (LiM(BH_4_)_3_Cl, M = La, Ce, Gd [70]).

### 3.4. Eutectic Formation Approach

A strategy to perform a more effective infiltration of RHC based on mixtures of borohydrides has been utilized recently, with reports of ternary and quaternary mixtures in the LiBH_4_-NaBH_4_-KBH_4_-Mg(BH_4_)_2_-Ca(BH_4_)_2_ system [152], LiBH_4_-NaBH_4_ [66,155], 0.62LiBH_4_-0.38NaBH_4_ [157], LiBH_4_-Ca(BH_4_)_2_ [158,162,182], LiBH_4_–KBH_4_ [160,170], LiBH_4_-Mg(BH_4_)_2_ [163] and various combinations of alkali- and alkali-earth metal borohydrides (0.725LiBH_4_-0.275KBH_4,_ 0.68NaBH_4_-0.32KBH_4,_ 0.4NaBH_4_-0.6 Mg(BH_4_)_2_) [234], LiBH_4_-M(BH_4_)_x_ (M = Na, K, Mg, Ca) [194].

### 3.5. Doping Strategy

Various dopants have been used so far to enhance the resorption parameters in hydrogen storage systems: TiO_2_(B) nanoparticles embedded in ordered carbon (for AB [288]), TiO (for LiBH_4_ [91]), TiCl_4_ confined in nanoporous carbon aerogel (for RHC: 2LiBH_4_–MgH_2_ [154]), nano-Ni (for a 0.62LiBH_4_-0.38NaBH_4_ [157]), ZrC (for LiAlH_4_ [121]), TM fluorides (for NaBH_4_, [230]), V_2_O_5_ or VO_2_ (for NaBH_4_ [199]), MWCNTs (for RHC: LiBH_4_–LiAlH_4_ [164]), various dopants introducing strain (Na, K, Al, F, or Cl for LiBH_4_ [61]), NiFe_2_O_4_ (for NaAlH_4_ [233]), YCl_3_ and Li_3_N (for RHC: 6Mg(NH_2_)_2_-9LiH-LiBH_4_ [177]) and ScOCl–functionalized carbon aerogel (for NaAlH_4_ [211]), C@TiO_2_/Ti_3_C_2_ (for NaAlH_4_ [226]).

In the context of dopants used, the presence of many forms of titanium (TiO, TiO_2_, TiO_2_(B), Ti_3_C_2_) typically supported by nanoscaffolds is noteworthy; this important role of Ti in particular may originate from the relative ease with which it can access multiple oxidation states, including metallic state (0, +2, +3, +4).

### 3.6. Electrolyte-Assisted Dehydrogenation

While nanoconfined borohydrides are usually studied at lower temperatures and confined in siliceous supports for solid-state electrolyte application [67], recent reports employ electrolyte systems as a means to accelerate de-/rehydrogenation of MgH_2_/Sn systems (LiBH_4_/KBH_4_ electrolyte [160]).

### 3.7. Additives

Many classes of compounds were used as additives [262]: (TM)H_x_ (TiH_2_ [187]); (TM)F_x_, chlorides (TiCl_3_ [228] [178], TiCl_4_ [154], FeCl_3_, YCl_3_ [177]); fluorides (of transition metals TM [196,230], TiF_3_ [161,183], CeF_3_ [100,225], K_2_NbF_7_ [124], NbF_5_ [102], YF_3_ [197,198], GdF_3_ [197] and ScF_3_ [198]); iodide (LiI [201]); tetrafluoroborates (LiBF_4_ [68]); main group hydrides (LiH, MgH_2_ and AlH_3_ [167]); sulfide (Ce_2_S_3_ [94] and SiS_2_ [103]); nitride (Li_3_N [177], BN and TiO_2_(B) [288]); borides and composites (MgB_2_/Mg [165]); ferrites (NiFe_2_O_4_ [233] and MgFe_2_O_4_ [200]) and hexaferrites (SrFe_12_O_19_ [120] and BaFe_12_O_19_ [123]); oxides (TiO [91], TiO_2_ [126,143,166,226], Fe_3_O_4_ [98], Al_2_O_3_ [310], V_2_O_5_ or VO_2_ [199], V_2_O_5_ supported by MWCNTs [311,312] and various oxides [112]); oxochlorides (ScOCl [211]); hydroxide (KOH [168] and LiB(OH)_4_ [108]); metals (nano-Ni [97,125,157,253,293], alkali metals [232], Zr [308], Al [234], Mg [104] and Ti(Al) [129]); intermetallics and derivatives (CoNiB [92] and Ni–Pt [243]); MXenes (Ti_3_C_2_ [159,225,226,249]); activated carbon [248] and SWCNTs [108].

### 3.8. Electron-Tuning of the Scaffold

Electron modification of the host component can oftentimes yield positive results. Three-dimensional mesoporous boron nitride BN, prepared as a C-replica of monolithic activated carbon, showed exceptional H_2_ storage capacity when nanoconfining AB (8.1 wt% at 100 °C, [292]), while h-BN confirmed a strong catalytic effect in dehydrogenation of LiAlH_4_ and Li_3_AlH_6_ intermediate generated from a LiAlH_4_@h-BN nanocomposite [131]. On a similar note, surface-modified AlN (with O-H and C-H groups providing electronic interactions of hydridic protons of LiBH_4_ and surface H^δ+^) was used as a scaffold for LiBH_4_ with increased reversible gravimetric capacity (6.1 wt% when cycling, [105]). Nitrogen-annealing of SWCNTs was shown to facilitate acidic and hydridic hydrogen interactions in a LiBH_4_@SWCNT-N nanocomposite, affording dehydrogenation onset as low as 108 °C [108].

### 3.9. Host Modification

Finally, various modifications of the scaffold allow tuning the reactivity towards improved cycling behavior of complex hydrides, and this approach oftentimes alters the desorption pathway. Various types of scaffolds were investigated: carbonaceous supports– SWCNTs, MWCNTs, carbon nanofibers CNFs, carbon nanospheres CNSs, carbon aerogel CA and ordered mesoporous carbon OMC. The latter includes CMK-3, graphene oxide GO, reduced graphene oxide rGO, modified C-scaffold by incorporation of metals (Al, Zr, Ti, Ni, Pd), siloxanic materials (SBA-15, MCM-41 etc.), metal salts (chlorides, fluorides, iodides, nitrates), bases (KOH), borides, sulfides, ferrites or MXenes. A survey of the nanostructuring procedure of complex hydrides reveals that most of the known classes of substances were used either in neat form (carbon, metals –Al, Ni etc.) or incorporating active catalysts/dopants to alter thermodynamics and accelerate de-/rehydrogenation reactions. These aspects were discussed in relation to the complex hydride utilized, in the previous sections.

## 4. Outlook and Future Directions

The current state-of-the-art in the field of nanosized complex hydrides was presented, with their most recent advances in hydrogen storage technology. Various tactics for achieving improved thermodynamics and for overcoming sluggish kinetics have been overviewed. These include using thermodynamic destabilization, cation/anion substitution, the eutectic formation approach, doping/additives, choosing the right scaffold and its electron-tuning, along with the—by now proverbial—nanoconfinement. The main advantages and the most resilient drawbacks of using these methods were discussed in relation to practical examples reported in the literature. Additionally, mechanistic insights are discussed and supported by the successful de-/rehydrogenation behavior of reported nanocomposites. Additionally, it is the author’s belief that among the improvement pillars presented before, the electronic synergy direction is not yet fully explored, and is an area where significant advances can be foreseen based on recent developments. Theoretical and experimental research efforts will join hands in order to better predict the composition, hydrogenation and recycling behavior of future nanocomposites for reaching an energy storage goal: the sustainable hydrogen economy.

## Figures and Tables

**Table 1:** LiBH_4_-based nanostructured systems and specific details regarding hydrogenation storage capacity.

Host Type	Catalyst/Active Intermediate; H_2_ Storing Composite	wt% H_2_	Ref.
High surface area graphite HSAG, carbon aerogels CA	LiC_x_ (^7^Li NMR), Li_2_B_12_H_12_ (^11^B NMR)	18.5	[77]
Hollow carbon nanospheres—modified by removal of carboxyl/ketone groups (HCNWs)	B, Li_2_B_8_H_8_ and Li_2_B_12_H_12_ (^11^B NMR)	~0.3 (reversible (0–2 wt% rev. in similar scaffolds)	[78]
N-Doped Graphene-Rich Aerogels Decorated with Nickel and Cobalt Nanoparticles	Ni, Co NPs generate Ni_2_B and CoB intermediates	8 wt% H_2_ at 325 °C(Co-NPs); 8 wt% H_2_ (Ni-NPs decorated CA, after rehydrogenation at 400 °C)	[79]
HNCs spheres (hollow carbon nanospheres)	Li_2_B_12_H1_2_ (by FTIR 2470 cm^−1^ and 756 cm^−1^)	0.65–0.47 wt% (LiBH_4_@HNCs)	[80]
Mesoporous carbon hollow spheres (MCHSs) with diameter 300 nm, and nanochannels 2–8 nm	Li_2_B_12_H_12_; No B_2_H_6_ in desorption (TPD data).	9.5 wt% DCLB-3 (70 wt% LiBH_4_, 30 wt% MCHSs); 10.9 wt% DCLB-2 (20 wt% MCHSs). 8.5 wt% reversible capacity (300 °C)	[81]
Activated charcoal (AC)	LiBH_4_/AC nanocomposite showed no B_2_H_6_ release (MS data)	T_onset,des_ = 190 °C; 13.6 wt% (400 °C); 6 wt% reversible (350 °C, 6 MPa)	[82]
Porous Hollow Carbon Nanospheres (PHCNSs)	Li_2_B_12_H_12_ (FTIR spectra, vibration at ~2490 cm^−1^)—weak, retarded by nanoconfined systems xLiBH_4_@yPHCNS (x,y)∈{(5,5);(6,4);(7,3);(8,2)}	8.1 wt% (350 °C,25 min; t_onset,des_ = 200 °C), 4.8 wt% reversible (5th cycle)	[83]
Carbon wrapped ultrafine Fe_3_O_4_ skeleton (p-Fe_3_O_4_@C)	Li_3_BO_3_, FeB, Fe_2_B and B proposed as intermediates; xLiBH_4_@yp-Fe_3_O_4_@C	7.8 wt% (350 °C, 30 min; t_onset_ = 175 °C, t_peak_ = 337 °C); 6.2 wt% reversible (20th cycle); 79.4 g/L volumetric hydrogen density	[84]
Electrospun nanofibers of polyacrylonitrile (PAN)-titanium (IV) isopropoxide composite (ACNF-Ti)	TiO_2_; LiBH_4_-ACNF-Ti compacted (868 MPa)	5–5.2 wt% (~75% of theoretical for 1:1 LiBH_4_:ACNF-T; t_des_ = 352–359 °C)	[85]
LiBH_4_ (5–10 nm)/Graphene/Ni nanocrystals (2–4 nm)	Li_2_B_12_H_12_, B_2_H_6_—are avoided; Li (XPS) slows kinetics above 315 °C, hence 300 °C chosen as maximum; nano-LiBH_4_/10Ni@20G	9.2 wt% (reversible, 300 °C, 100th cycle); 11.6 wt% (600 °C, t_onset_ = 130 °C).	[86]
Carbon matrix prepared by resorcinol-formaldehyde method (4 types mesopores: 6, 10, 15, 25 nm; 0.82 cm^3^ = V_t,pores_)	LiH + B—negatively impact reversible behavior; LiH-catalytic destabilization of LiBH_4_	10 wt% (S10/40); poor reversibility; ~12.5 wt% (50% LiBH_4_ loading)	[87]
LiBH_4_ 50–60 nm prepared/no support	close contact LiH and B crucial for reversibility	12.1 wt% of reversible (400 °C, t_onset,des_~190 °C; t_onset,rehyd_~165 °C, 100 bar H_2_)	[88]
Core–shell structure of CNT@ porous carbon @TiO_2_ hybrid	TiO_2_; LiTiO_2_ and TiB_2_ destabilize LiBH_4_	17.7 wt% (500 °C); 7.3 wt% H_2_ (60 min, 320 °C); 5.1 wt% (20th cycle)	[89]
Nanosheet-like LiBH_4_·H_2_O (20–30 nm thick) by freeze-drying technique	H+ from H_2_O ligand; LiBO_2_ identified	10 wt% (at 70 °C; t_onset,des_ = 50 °C)	[90]
LiBH_4_ heated with Ti(OEt)_4_ (precatalyst)	TiO in-situ introduced into LiBH_4_, yielding LiBH_4_-0.06TiO. Li_3_BO_3_ and TiH_2_ produced can act as catalysts for LiBH_4_	9 wt% (400 °C, 30 min); 9 wt% (reversible, 500 °C, 50 bar H_2_, t_onset,rehyd_ = 150 °C).	[91]
Carbon aerogels (CA): 9.7 nm, 0.843 cm^3^/g, 147.1 m^2^/g	CoNiB-NPs loaded into carbon aerogels (CA), forming LiBH_4_@CA@CoNiB	15.9 wt% (600 °C); 14.5 wt% (400 °C, 300 min.; t_onset_ = 192 °C, t_peak_ = 320 °C); 9.33 wt% (350 °C, 30 min)	[92]
Nanoporous silica (3.3, 3.6, 4.2, 4.4, 5.9, 6.1 nm) and carbon scaffolds (3.1, 3.8, 5.6, 20 nm) (comparison)	LiBH_4_@SiO_2_ (SBA-15); LiBH_4_@C	–; (thermodynamc investigation study-phase transition shift in solid-solid phase transition)	[93]
Ce_2_S_3_ scaffolds (solvothermal method, 100–200 nm diameter)	Li_2_S and CeB_6_ have co-catalytic effects (rehydrogenation) (Reaction (11)). LiBH_4_ + 20 wt% Ce_3_S_3_	4.0 wt% (3000 s, 400 °C; t_onset,dehyd_ = 250 °C); rehydrogenation (400 °C, 5 MPa H_2_)	[94]
Activated carbon nanofibers (ACNF*tt*, *tt* = activation time during heating, 15–75; S_BET_ = 2752 m^2^/g, V_tot_ (2.17 mL/g).	B_2_H_6_ is suppressed; C surface shows catalytic effect. Li_2_B_12_H_12_ formed (lowers H_2_ wt% during cycling).	11.7 wt% (1st), 7.1 wt% (2nd cycle); 81% of theoretical H_2_ capacity (t_onset_ = 125 °C, t_peak_ = 170 °C)	[95]
Ti_2_C_3_ MXene	MXene catalyst; LiBH_4_@2Ti_3_C_2_ hybrid investigated showed best results. Ti(0)//Ti(II)/ Ti(III)/Ti(IV)-TiO_2_ complex redox speciation of Ti with possible catalytic role (XPS data)	9.6 wt% (380 °C, 1 h; t_onset_ = 172.6 °C); partial reversibility: 6.5 wt%—2nd cycle; 5.5 wt%—3rd cycle (300 °C, 95 bar H_2_; 48% capacity degradation at 3rd cycle)	[96]
Ni(0)	nanoporous Ni-based alloy (np-Ni); 1 LiBH_4_/5 np-Ni	11.9 wt% (400 °C; t_onset_ = 70 °C); 8.2 wt% (2nd cycle, t_onset_ = 60 °C).	[97]
Porous graphene support	Fe_3_O_4_ nanoclusters; Fe_3_O_4_@rGO destabilizer and catalyst precursor; Li_3_BO_3_ catalyst formed in situ. LiBH_4_–20 wt% Fe_3_O_4_@rGO investigated.	3.36 wt% (400 °C, 1000s; t_onset_ = 74 °C); 5.74 wt% uptake (400 °C, 5 MPa H_2_); 3.73 wt% reversible (5th cycle)	[98]
Graphene in a mesoporous resorcinol–formaldehyde matrix (600 m^2^/g, 6.1 nm, 1.53 cm^3^/g)	B_2_H_6_ avoided (IR); graphene@MC catalytic effect	13 wt% (400 °C; t_onset_ = 253 °C); 6 wt% (2nd cycle); reversible system (rehydrogenation at 400 °C, 5 h, 60 bar H_2_)	[99]
Activated carbon	LiBH_4_-AC-CeF_3_; CeF_3_—catalyst	13.1 wt% (LiBH_4_-AC; 8.1 wt% reversible–4th cycle); 12.8 wt% (LiBH_4_-AC-CeF_3_; 9.3 wt% reversible–4th cycle) (t_onset_ = 160 °C)	[100]
Modified carbon nanotubes: SWCNTs, MWCNTs	CNTs; LiBH_4_@SWCNT, LiBH_4_@SWCNTs (BM), LiBH_4_@MWCNTs, and LiBH_4_@MWCNTs (BM)	11 wt% (LiBH_4_@ MWCNTs, 450 °C); 11 wt% (LiBH_4_@MWCNTs (BM), 400 °C)	[101]
Highly ordered mesoporous carbon, C-SBA-15 replica, 1321 m^2^/g; 1.25 cm^3^/g (with NbF_5_ NPs)	MC-NbF_5_ (catalytic effect of 10 wt% precatalyst NbF_5_); presumed active catalysts: Nb_2_O_5_, NbH_x_, NbB_2_ (Reaction (12)). LiBH_4_@MC-NbF_5_ system investigated	6.52 wt% (200 °C) for LiBH_4_@MC-NbF_5_. t_onset_ = 150 °C (LiBH_4_@MC-NbF_5_), 205 °C (LiBH_4_@MC), 282 °C (LiBH_4_-NbF_5_-BM); 10.65 wt% rehydrogenation (mild conditions: 200 °C, 60 bar H_2_).	[102]
SiS_2_	x LiBH_4_–SiS_2_ (x = 2–8) investigated. No B_2_H_6_ during release. First principles calculations identified various polymorphs, and point to decomposition Reaction (13).	For x = 6: 8.2 wt% (t_onset_ = 92 °C), 2.4 wt% reversible. For x = 2: 4.3 wt% (385 °C, t_onset_ = 88 °C), 1.5 wt% rehydrog (385 °C, 160 bar H_2_)	[103]
Porous Mg scaffold (sintering a NaMgH_3_ pellet sintered at 450 °C under dynamic vacuum, removing molten Na) (14)	Mg-porous (26 m^2^/g; 1.25 cm^3^/g); Reactions (15) and (16); MgB_2_ as destabilizing agent for 2LiBH_4_-Mg Investigated materials: Mg_porous_- x wt% LiBH_4_ (x = 12.78–35.05)	4.81 wt% (2LiBH_4_:Mg); 7.12 wt% (PMg33); 9.69/9.74 wt% (2 LiBH_4_:MgH_2_ powder/pellet)	[104]
Surface-Modified AlN	AlN@LiBH_4_; AlN synthesized by alcoholysis of LiBH_4_ (by freeze-drying, AlN contains O–H and C–H groups (Equation (17)), best catalytic effect). x AlBH_4_: y AlN; (x,y)∈{(1,1);(3,2);(2,1);(4,1)}. Li_2_B_12_H_12_ forms only during 1st cycle, then it decomposes due to AlN. Li_3_BO_3_ in situ with potential catalytic effect (IR data: 746, 1265, 1315 cm^−1^).	5.2 wt% (1:1); 6.8 wt% (3:2); 7.7 wt% (2:1); 8.3 wt% (4:1) for LiBH_4_-AlN composites. Rehydrogenation at 400 °C, 10 MPa, 24 h, with capacity loss (3.7 wt%). 6.1 wt%—optimized 2:1 composite, stable capacity (400 °C, 10 MPa H_2_).	[105]
C:MSU-H 2D ordered mesoporous carbon replica	C:MSU-H pores; three loadings studied: 8, 20 and 40 wt% LiBH4@C-MSU-H	1.01 wt% (325 °C, t_onset_ = 150 °C; 92% of theoretical maximum for 8 wt% LiBH_4_-MSU-H); 2.7 wt% (375 °C, 96.4% of theoretical maximum for 20 wt% LiBH_4_-C-MSU-H); 0.79 wt% (2nd cycle, 200 °C onset, 8 wt% LiBH_4_-MSU-H)	[106]
Mo:MSU-H	Molibdate precursor decomposes thermally at 550 °C (4 h) to give active MoO_3_ catalyst. Mass ration LiBH_4_:Mo-MSU-H = 5:1 (~70% host pore filling)	11.2 wt% H_2_ (5.2 wt% rehydrogenation 450 °C, 80 bar H_2_)	[107]
Single-Walled Carbon Nanotubes (SWCNTs)	LiB(OH)_4_, Li_2_CO_3_ and LiBO_2_	4.0 wt% (153–368 °C, SWLiB-A); 4.3 wt% (108–433 °C, SWLiB-N); partical reversibility (100–150 °C, 5–10 bar H_2_)	[108]
Al Derived from AlH_3_ (Al*) shows superior hydrogenation effect compared to commercial Al.	LiBH_4_/Al* composite (Equations (18) and (19)): 2LiBH_4_ + Al → 2LiH + AlB_2_ + 3H_2_ (18) LiH + Al → LiAl + 1/2H_2_ (19). B_2_H_6_ and an intriguing “Li-Al-B-H” phase detected.	6.2 wt% (LiBH_4_/Al*); 5.5 wt% (LiBH_4_/Al). Reversibility (5.5 wt%) at 400 °C under 8 MPa H_2_ (Equation (20)): LiH + LiAl + AlB_2_ + 7/2H_2_ ↔ 2LiBH_4_ + 2Al. (20)	[109]

**Table 2:** LiAlH_4_-based nanostructured systems and details regarding hydrogenation storage capacity.

Host Type	Catalyst/Active Intermediate; H_2_ Storing Composite	wt% H_2_	Ref.
Graphite	LiC_x_ (instead of LiH); LiAlH_4_/HSAG nanocomposite (NC-HSAG)	10% wt% (300 °C, LiAlH_4_/HSAG)	[77]
High surface area graphite (HSAG)	Li_3_AlH_6_ active intermediate; no other catalyst was used	0.6 wt% partially reversible storage at 300 °C (30 min) (t_onset_ = 135 °C, 7 MPa H_2_)	[116]
NiCo_2_O_4_@rGO	NiCo_2_O_4_, rGO	6.28 wt% (t_onset_ = 62.7 °C); 4.0 wt% (isothermal 150 °C, 20 min.)	[117]
N-doped CMK-3 carbon (NCMK-3)	N-sites in LiAlH_4_@NCMK-3; LiAlH_4_@CMK-3 synthesized for comparison.	~1.1 wt% (240 °C, t_onset_ = 126 °C); >80% reversibility of LiAlH_4_ (50 °C, 100 MPa H_2_)	[118]
(2D) layered Ti_3_C_2_	catalytic effect of 2D Ti_3_C_2_ in LiAlH_4_ + 5 wt% Ti_3_C_2_, which forms under ball milling active Ti(0) and Ti(+3) catalytic sites (XPS data)	6.5 wt% (58.6 °C onset); 5.5 wt% (200 °C, 35 min); 3.9 wt% (120 °C, 40 min)	[119]
-	SrFe_12_O_19_ addition., with active catalyst formed LiFeO_2_/Sr-phases	5.54 wt% (130 °C, 20 min) for LiAlH_4_ + 10 wt% SrFe_12_O_19_	[120]
-	ZrC powder, x mol% ZrC-doped LiAlH_4_ (x = 1,2,5,10).	6.61 wt% (for 1 mol%-doped LiAlH_4_); 5.62 wt% (145 °C, 180 min); 5.48 wt% (130 °C, 180 min); 4.08 wt% (115 °C, 180 min).	[121]
-	2LiBH_4_-M (M = Al, LiAlH_4_, Li_3_AlH_6_)	8 wt% (2LiBH_4_-LiAlH_4_, 150 min)	[122]
-	Fe, LiFeO_2_ and amorphous Ba or Ba-containing species (XRD, after desorption at 250 °C); LiAlH_4_-10 wt% BaFe_12_O_19_	4.2 wt% (90 °C, 2.5 h); ~6.0 wt% (250 °C, t_onset_ = 95 °C)	[123]
-	K_2_NbF_7_ as precatalyst for in situ formed NbF_4_, LiF, and K-containing species; LiAlH_4_ + 10 wt% K_2_NbF_7_	~ 6.2 wt% (250ׄ°C, t_onset_ ~75 °C); 3.2 wt% (120 min, 90 °C, kinetic study)	[124]
Nickel-Containing Porous Carbon Sheets (Ni-PCS)	Ni, C_porous_; LiAlH_4_-5 wt% Ni-PCS (11.6 µm)	8.14 wt% (LiAlH_4_ + PCS); 7.97 wt% (LiAlH_4_ + Ni-PCS)	[125]
TiO_2_/Hierarchically Porous Carbon	TiO_2_/C_nanoporous_; LAH-TiO+/HPC with postulated role of defect redox sites Ti^4+^/Ti^3+^/Ti^2+^ in 0.62/0.22/0.16 atomic ration (XPS).	6.2 wt% H_2_ (60 min, 160 °C); 4.3 wt% (40 min, 130 °C; t_onset_ = 64 °C, t_peak_ = 115 °C); partial rehydrogenation possible (300 °C, 4 MPa)	[126]
Hollow carbon nanospheres (HCNs)	LiAlH_4_@HCNs	5.8 wt%/expected 2.3 wt% (solvent traces; sharp onset 146 °C; full conversion to LiH, 90 min). 0.37 wt% reversible rehydrogenation to LiAlH_4_ (150 °C, 8 MPa H_2_; vs 2.4 wt% max theoretical for used loading).	[127]
-	LiAlH_4_.xMe_2_O; adduct formation excludes Li_3_AlH_6_ or other intermediates.	N/A; proof-of-concept regarding rehydrogenation potential of LiAlH_4_.	[128]
-	LiAlH_4_ · 4THF regeneration; Ti-catalyzed Al (TiCl_3_).	4.5 wt& regeneration at 398K using Al* [Al(Ti)- 2 mol% Ti]	[129]
-	Surfactant used as stabilizer for NP size manipulation.	–; desolvation from LiAlH_4_-X-N (X-solvent; N = 0.1, 1, 5, 10)	[59]
FGi (Fluorographite)	LiF (and possibly LiAlF_4_), Al_4_C_3_—active catalyst(s), generated in LiAlH_4_-xFGi composites	6.25 wt%, with 5.7 wt% (ultra-fast: seconds, t_onset_ = 61.2 °C, 65 °C main step) for LiAlH_4_-40FGi.	[130]
h-BN	LiAlH_4_/x wt% h-BN (x = 0,4,14,40). Composites. h-BN has a catalytic effect on Li_3_AlH_6_ decomposition (second step desorption).	7.6 wt% (LiAlH_4_/4 wt% h-BN), 6.8 wt% (LiAlH_4_/14 wt% h-BN), 4.7 wt% (LiAlH_4_/40 wt% h-BN).	[131]

**Table 3:** Examples of binary and tertiary RHC with corresponding components.

RHC Type	Components	RHC Type	Components
Binary	Ca(BH_4_)_2_ + MgH_2_	Ternary	Ca(BH_4_)_2_ + 2LiBH_4_ + 2MgH_2_
	2LiBH_4_-MgH_2_		NaAlH_4_-MgH_2_-LiBH_4_
	2LiBH_4_-2.5Mg_2_NiH_4_		Ca(BH_4_)_2_-LiBH_4_-MgH_2_
	Na_3_AlH_6_-3MgH_2_		LiNH_2_-MgH_2_-Ca(BH_4_)_2_
	2NaAlH_4_-Ca(BH_4_)_2_		LiAlH_4_-MgH_2_-LiBH_4_
	NaBH_4_-Li_3_AlH_6_		LiBH_4_-CaH_2_-MgH_2_
	Ca(BH_4_)_2_-Mg(AlH_4_)_2_		MgH_2_-Na_3_AlH_6_-LiBH_4_
	Ca(BH_4_)_2_-LiNH_2_		LiBH_4_-Mg(NH_2_)_2_-LiH
	NaAlH_4_-Ca(BH_4_)_2_		LiNH_2_-MgH_2_-LiBH_4_
	LiBH_4_-Mg(BH_4_)_2_-(TiF_3_)		LiNH_2_-LiH-Mg(BH_4_)_2_
	Na_3_AlH_6_-LiBH_4_ –(MgFe_2_O_4_)		
	MgH_2_-LiAlH_4_-(SeFe_12_O_19_)		
	MgH_2_-NaAlH_4_-(TiF_3_)		
Ternary	LiBH_4_-NaBH_4_-M(BH_4_)_2_ M = Mg, Ca	Quaternary	LiBH_4_-NaBH_4_-KBH_4_-Mg(BH_4_)_2_
	LiBH_4_-KBH_4_-M(BH_4_)_2_ M = Mg, Ca		LiBH_4_-NaBH_4_-KBH_4_-Ca(BH_4_)_2_
	LiBH_4_-Mg(BH_4_)_2_-Ca(BH_4_)_2_		LiBH_4_-NaBH_4_-Mg(BH_4_)_2_-Ca(BH_4_)_2_
	NaBH_4_-KBH_4_-M(BH_4_)_2_ M = Mg, Ca		LiBH_4_-KBH_4_-Mg(BH_4_)_2_-Ca(BH_4_)_2_
	MBH_4_-Mg(BH_4_)_2_-Ca(BH_4_)_2_ M = Na, K		NaBH_4_-KBH_4_-Mg(BH_4_)_2_-Ca(BH_4_)_2_

**Table 4:** Examples of proposed chemical reactions in binary and tertiary RHCs.

RHC Type	Chemical Reaction(s) Proposed	
Binary	Ca(BH_4_)_2_ + MgH_2_ → CaH_2_ + MgB_2_ + 4H_2_	(37)
	Ca(BH_4_)_2_ + MgH_2_ → 2/3CaH_2_ + 1/3CaB_6_ + Mg+ 13/3 H_2_	(38)
	Ca(BH_4_)_2_ + MgH_2_ → CaH_2_ + 2B + Mg + 3H_2_	(39)
	2LiBH_4_ + MgH_2_ → 2LiBH_4_ + Mg + H_2_	(40)
	2LiBH_4_ + Mg + H_2_ → 2LiH + MgB_2_ + 4H_2_	(41)
	2LiBH_4_ + 2.5Mg_2_NiH_4_ → 2LiH + MgNi_2.5_B_2_ + 4MgH_2_ + 4H_2_	(42)
	Na_3_AlH_6_ + 3MgH_2_ → 3NaMgH_3_ + Al + 3/2 H_2_	(43)
	2NaAlH_4_ + Ca(BH_4_)_2_ → Ca(AlH_4_)_2_ + 2NaBH_4_	(44)
Tertiary	Ca(BH_4_)_2_ + 2LiBH_4_ + 2MgH_2_ → 1/3CaH_2_ + 2/3CaB_6_ + 2LiH + 2Mg + 26/3 H_2_	(45)

**Table 5:** Li-RHC-based nanostructured systems and specific details regarding hydrogenation storage capacity.

Host Type	Catalyst/Active Intermediate; H_2_ Storing Composite RHC	wt% H_2_	Ref.
Adaptive poly(4-methyl-1-pentene), TPX™ Polymer	2LiH + MgB_2_+ 7.5(3TiCl_3_·AlCl_3_) and 2LiH + MgB_2_+ 7.5(3TiCl_3_·AlCl_3_)@TPX	~9.1 wt% (non-confined); 7.3 wt% (TPX-confined)	[140]
Carbon aerogel scaffold with pore size *D*_max_ ∼21 nm	Al_12_Mg_17_ and Mg_1−*x*_Al*_x_*B_2_ proposed as intermediates; 2 LiBH_4_: MgH_2_	4.3 wt%; ~4 wt% reversible	[142]
-	Li-RHC (2LiH + MgB_2_/2LiBH_4_ + MgH_2_) composite doped with TiO_2_ (Li-RHC-Ti).	10.1 wt% (stable after cycles 11–15)	[143]
-	2 LiH + MgB_2_/2 LiBH_4_- MgH_2_. Additive used: 0.05 mol TiCl_3_/mol MgB_2_	- (kinetic study by PCI isotherms in the 350–400 °C temperature interval).	[144]
Resorcinol formaldehyde carbon aerogel (RFC): 1.1452 cm^3^/g, 687.1 m^2^/g.	2LiBH_4_-LiAlH_4_/RFC; AlB_2_ as catalyst in-situ. Li_2_B_12_H_12_ was also identified as potential intermediate.	5.7 wt% (reversible) (t_onset, step 1_ = 100 °C; t_onset,step 2_ = 280 °C); rehydrogenation at 350 °C, 5 MPa H_2_ (5 h)	[145]
Activated carbon nanofibers (ACNF)	LiBH_4_-LiAlH_4_	6.6 wt% (milled LiBH_4_-LiAlH_4_); 2.7 wt% (nano RHC-ACNF)	[146]
Mesoporous carbon aerogel CA (*D*_max_ = 30 nm, *S*_BET_ = 689 m^2^/g and *V*_tot_ = 1.21 cm^3^/g)	2LiBH_4_-NaAlH_4_; AlB_2_ and Al detected as final dehydrogenation products (can act as catalysts, also Li_3_AlH_6_ and LiAl_3_ was observed); C surface can also exhibit catalytic properties.	2.4 wt% (2.6 wt% theoretical; 33% pore filling); 7.8 wt% (estimated based on 100% pore filling of CA); t_onset_ = 100 °C; 83% reversible of theoretical wt% H_2_ (RHC-CA); 47% reversible (RHC). Rehydrogenation at 126 bar H_2_, 400 °C, up to 10 h (NaBH_4_ recovered)	[147]
Porous hollow carbon nanospheres (HCNS)	LMBH: LiBH_4_-Mg(BH_4_)_2_ in 55:45 mole ratio, eutectic formation reported. (LMBH@HCNS final composite). Stable [B_12_H_12_]^2−^ anion was detected by FTIR (dehydrogenated products)	4.3 wt% (33LMBH@HCNS); 6.1 wt% (50LMBH@HCNS); 8.1 wt% (67LMBH@HCNS). Rehydrogenation (100 bar H_2_, 6 h, 280 °C)	[148]
Nano–Ni(doped)in Ni/C scaffold	2LiBH_4_–MgH_2_; final composite: 2LiBH_4_–MgH_2_–15%Ni/C and reactive intermediate (nanosized) Ni_4_B_3_ as a pre-catalyst for MgNi_3_B_2_ species.	~9 wt%	[149]
Activated carbon	2LiBH_4_-MgH_2_; “LiBH_4_-MgH_2_-AC” composite; Li_2_B_12_H_12—_intermediate (incomplete rehydrogenation)	3.56–4.55 wt% (dehydrogenation); 2.03–3.28 wt% H_2_ (rehydrogenation) for the first 5 cycles	[150]
Carbon aerogel scaffold (CAS) doped with ZrCl_4_	2LiBH_4_–MgH_2_; ZrCl_4_ catalyst/dopant (Zr-P63mmc and ZrB_2_ detected by XRD in dehydrogenated samples).	2.5–5.4 wt% (nano-RHC:AC, 1:3–1:1); 8.7 wt% (milled RHC)	[151]
-	ternary and quaternary mixtures in the system LiBH_4_-NaBH_4_-KBH_4_-Mg(BH_4_)_2_-Ca(BH_4_)_2_:	various (structural investigation study)	[152]
-	2 LiBH_4_–2.5 Mg_2_NiH_4_; reversible H_2_ storage via MgNi_2.5_B_2_ (intermediate)	~4.8 wt% (under H_2_ pressure of 1 or 5 bar)	[153]
Carbon aerogel scaffold (CAS)	2LiBH_4_–MgH_2_–0.13TiCl_4_; TiCl_4_ catalyst ([B_12_H_12_]^2−^ stable intermediate identified at 2480 cm^−1^).	3.6 wt% (1.5 h, t*_onset_=* 140 °C, release in range 140–380 °C)	[154]
-	LiBH_4_–NaBH_4_	N/A	[155]
Ordered Nanoporous Carbon (NPCs) 1012 m^2^/g and 0.65 cm^3^/g	LiBH_4_–Mg(BH_4_)_2_	~7 wt% (1st cycle), ~ 1 wt% (2nd cycle)	[156]
-	0.62LiBH_4_-0.38NaBH_4_; nano-Ni as catalyst, producing 0.91 (0.62LiBH_4_-0.38NaBH_4_)-0.09Ni catalyzed RHC.	8.1 wt% H_2_ (t_peak_ = 350 °C, up to 650 °C, Ar). Decreasing performace in subsequent cycles (5.1 wt%, 1.1 wt% and 0.6 wt%).	[157]
-	2LiBH_4_ + M (M = LiAlH_4_, Li_3_AlH_6_)	8 wt% (2LiBH_4_-LiAlH_4_), 9.1 wt% (2LiBH_4_-Li_3_AlH_6_, 150 min)	[122]
Nanoporous silica (fumed silica; mesoporous MCM-41 and SBA-15)	LiBH_4_–Ca(BH_4_)_2_ (4:1, 2.1:1 -eutectic composition)	N/A	[158]
2D-MXene (Ti_3_C_2_)	4MgH_2_-LiAlH_4_-Ti_3_C_2_ (Ti_3_C_2_ MXene acts as pre-catalyst of Ti(0) species in 4MgH_2_-LiAlH_4_ RHC). TiH_1.942_ intermediate identified in situ hints at reaction Ti(0) with RHC.	~ 6.5 wt% (onset at 336K, peak at 594 K)	[159]
-	LiBH_4_/KBH_4_ as eutectic catalyst for MgH_2_/Sn destabilized hydride, and for rehydrogenation of spent MgB_2_ to Mg(BH_4_)_2_. 0.025 MgI_2_ was found to bring further kinetic enhancement	~1.7 wt% (t_onset_ = 150 °C; 40 h); ~2 wt% rehydrogenated (920–1000 bar. at 215–175 °C); 1.9 wt% (2nd dehydrogenation).	[160]
-	4LiAlH_4_–Mg_2_NiH_4_; Ti-based catalyst used (3 mol% TiF_3_); potential intermediacy of Al_3_Ti (Ti0) phase (in-situ XPS data). [F-] effect possibly due to replacement of H in Al-species, forming [AlF_4_]^−^ and [AlF_6_]^3−^ which facilitate dehydrogenation.	5.62 wt% (TiF_3_-cat.); 5.02–5.09 (undoped, Ti-or TiO_2_-cat.), with t_onset_ = 50 °C for 1st step (21); t_onset_ = 128 °C for 2nd step (22); 1.38 wt% (reversible rehydrog., 3 MPa H_2_, 300 °C, 150 s).	[161]
Mesoporous carbon CMK-3 (C-replica of SBA-15 silica)	x LiBH_4_–(1 − x) Ca(BH_4_)_2_ eutectic with x = 0.50, 0.60, 0.65, 0.68, 0.70, 0.75, and 0.80 (mp~200 °C). CMK–3 porosity: 1229 m_2_/g, 3.5 nm pore size, 1.63 cm^3^/g–0.59 cm^3^/g micro & 1.04 cm^3^/g meso)	~11 wt% (eutectic RHC); ~ 5 wt% (RHC@CMK-3, up to 400 °C, t_peak_~300 °C, 2 h, under 3 bar H_2_). Rehydrogenation (400 °C, 24 h, 110 bar H_2_)	[162]
Activated carbon aerogel CA (689–2660 m^2^/g, 1.21–3.13 cm^3^/g)	0.55LiBH_4_–0.45Mg(BH_4_)_2_	13.3 wt% H_2_ (RHC@CA); 8.4 wt% (RHC). Reversible: 8.3 wt% (RHC@CA,4th cycle); 3.1 wt% (RHC, 4th cycle).	[163]
MWCNTs	LiBH_4_–LiAlH_4_; active phases/catalysts formed in-situ: AlB_2_ and LiAl.	2.0–3.0 wt% (5.5 h, 400 °C for RHC@MWCNTs), 2.3–2.8 wt% (RHC) decreasing after 3 cycles.	[164]
-	Mg@NaBH_4_/MgB_2_ core-shell structures	5.98 wt%	[165]
-	4MgH_2_ + LiAlH_4_, TiO_2_ catalyst. Active catalyst phases are considered Al_3_Ti and TiH_2_.	4.7 wt% (450 °C); 2.5 wt% (13 min, 320 °C); rehydrogenation confirmed (2.9 wt%, 10 min)	[166]
-	LiBH_4_-x AlH_3_ (x = 0.5, 1.0, 2.0)	11.0 wt% (release, 450 °C, 6 h) for LiBH_4_ − 0.5 AlH_3_ (best result); 3.2 wt% reversible capacity (2nd dehydrogenation, 400 °C, 5 MPa H_2_, 5 h).	[167]
-	Mg(NH_2_)_2_–2LiH–0.07KOH	4.5–4.9 wt% under 0–5 bar H_2_,	[168]
Carbon aerogel scaffold (CAS)	2LiBH_4_–NaAlH_4_; LiNa_2_AlH_6_ was identified as short-lived intermediate (60)	2.39 wt% (400 °C, for 2:1 weight ratio CAS-RHC); ~2.0 wt% reversible (4th cycle, 400 °C, 80 bar H_2_, 12 h)	[169]
CMK-3 Type Carbon (5 nm pore size)	LiBH_4_-KBH_4_ Eutectic (0.725 LiBH_4_–0.275 KBH_4_)	reversible 2.5–3 wt% (5th cycle)	[170]
	3LiBH_4_ + Er(BH_4_)_3_ + 3LiH	4.2 (1st cycle); 3.7 (2nd cycle); 3.5 (3rd cycle) out of 6 wt% (theoretically accessible for given RHC)	[171]
Activated carbon nanofibers (ACNFs)	2LiBH_4_-MgH_2_; ACNFs as dopant (30 wt%)	1.8 to 4.5 wt% H_2_ (reversible)	[172]
-	K_2_Mn(NH_2_)_4_–8LiH; K_3_MnH_5_ and K–Mn-species (probably hydrides)	6 wt% (open system); rehydrogenation almost complete (230 °C, 50 bar H_2_, very fast at 1 wt%/min); 3 wt% reversible (375 °C release, 300 °C uptake)	[173]
CO_2_-activated carbon aerogel (37–38 nm; 690–2358 m^2^/g)	0.62LiBH_4_–0.38NaBH_4_ (eutectic)	1.6 wt% (4th cycle, 22% of full theoretical capacity)	[174]
Activated carbon aerogel (pristine: 689 m^2^/g, 1.21 cm^3^/g and CO_2_-activated: 2660 m^2^/g, 3.13 cm^3^/g)	0.7LiBH_4_–0.3Ca(BH_4_)_2_	12.08 wt% (RHC), 7.71 wt% (RHC-CA_CO2_), 3.36 (RHC-CA)	[175]
Carbon nanoscaffold (IRH33, 1.17 cm^3^ g^−1^, 2587 m^2^ g^−1^_,_ 0.5 to 4.5 nm pores)	Li^11^BD_4_–Mg(^11^BD_4_)_2_	4.5 wt% (460 °C, 6.7 wt% maximum theoretical based on 46% filling, 14.6 wt% maximum of RHC)	[176]
-	NaBH_4_-Li_3_AlH_6_	4.2 wt% (330 °C); 6.1 wt% (420 °C, 30 atm, 60 min)	[132]
-	6Mg(NH_2_)_2_–9LiH–LiBH_4_ (additives: YCl_3_ and Li_3_N)	4.2 wt% uptake (180 °C, 85 bar H_2_, 8 min or 90 °C, 185 bar H_2_)	[177]
-	2LiH + MgB_2_/2LiBH_4_ + MgH_2_; 0.05 TiCl_3_ was used as additive	N/A	[144]
Resorcinol–formaldehyde carbon aerogel scaffold (RF–CAS)	2LiBH_4_–MgH_2_–TiCl_3_	3.6–3.75 wt% (1st release, 95–98.6% of maximum theoretical; 425 °C, 3.4 bar, 4.5 h); 3.25 wt% (2nd release, 8 h); 3.6–3.75 (3rd, 4th release, 15 h); uptake (425 °C, 130–145 bar)	[178]
Mesoporous carbon (MC) scaffolds	2LiBH_4_-LiAlH_4_	10 wt% (300 °C, 300 min)	[179]
-	LiBH_4_-MgH_2_; Fe_3_B was used as additive.	4.11 wt% (1st cycle, des.); 2.35 wt% (1st abs); 2.89 wt% (7th cycle, des.); 3.32 wt% (7th cycle, abs.).	[180]
-	LiBH_4_-NaBH_4_-KBH_4_-Mg(BH_4_)_2_-Ca(BH_4_)_2_	N/A (up tp 500 °C, incomplete desorption)	[181]
Carbon aerogel scaffold CAS (*S*_BET_ = 2421 ± 189 m^2^/g, *V*_tot_ = 2.46 ± 0.46 mL/g, 13 nm pore size)	0.68LiBH_4_–0.32Ca(BH_4_)_2_ (“LiCa” eutectic)	5.4 wt% (bulk RHC), 3.7 wt% (RHC@CAS), 1.1 wt% (LiBH_4_@CAS), at 5th cycle.	[182]
-	2NaAlH_4_ + Ca(BH_4_)_2_; 5 wt% TiF_3_ as additive and precursor of active catalytic species Al_3_Ti and CaF_2_ (synergy)	~8 wt% (des, RHC-TiF_3_); ~7.5 wt% (des., RHC); 4.1 wt% (abs, RHC-TiF_3_); 3.5 wt% (abs, RHC), after 60 min, 420 °C, 30 atm H_2_.	[183]
2D MXene—nanoadditive (x wt%, x = 1, 3, 5, 7 wt%)	2LiH + MgB_2_/2LiBH_4_ + MgH_2_; 2D Ti_3_C_2_ MXene as additive	9.0 wt% H_2_ (400 °C, 20 min). Regeneration by hydrogenated at 350 °C/10 MPa, <5 min.	[184]
Manoporous carbon host (CAS; 458 m^2^/g; 0.56cm^3^/g; 4.86 nm pore size)	LiBH_4_-MgH_2_-NaAlH_4_ transforming into LiAlH_4_–MgH_2_–NaBH_4_, Intermediate phases detected as Li_3_Mg_7_, Mg_17_Al_12_ (PXD).	3.0 wt% (t_peak_ = 329 °C, up to 450 °C, ~72% of maximum theoretical capacity of RHC@CAS)	[185]
–	(1 − x) LiBH_4_– x Mg_2_FeH_6_, x = 0.25, 0.5, 0.75.	6.0 wt% (580–630K; rehydrogenation with no H_2_ wt% loss after 4 cycles)	[186]
NP-TiH_2_@G (TiH_2_ nanoplates 50 nm lateral; 15 nm thick; additive)	NaAlH_4_-7 wt%NP-TiH_2_@G	5 wt% (reversible, full dehydrogenation at 80 °C; rehydrogenation 30 °C, 100 atm H_2_)	[187]

Compared to other reports [147], changing the reagent molar ratio (from 2:1 to 1:1) [185], in the case of LiBH_4_-NaAlH_4_ RHC, can yield different products—some of which with proven catalytic activity in hydrogenation cycling (AlB_2_ for instance [147]). It seems feasible that tuning the molar ratio of reagents and the consideration of possible chemical reactions occurring during de/rehydrogenation can shape the future of RHC, including avenues poorly explored thus far.

## Data Availability

Not applicable.
